# Supplementary material for: Idiosyncratic, Retinotopic Bias in Face Identification Modulated by Familiarity
Source: eNeuro. 2018 Oct 4;5(5):ENEURO.0054-18.2018. doi: 10.1523/ENEURO.0054-18.2018 (PMC6171739; doi:10.1523/ENEURO.0054-18.2018)
Supplement: Extended Data — The archive contains data from both experiments, as well as the analysis scripts. Download Extended Data 1, ZIP file. [file sup_enu-eN-NWR-0054-18-s02.zip › famretino2-3.0.0/exp1/code/analysis_exp1.nb.html]

Experiment 1


Code 

- Show All Code
- Hide All Code
- Download Rmd

# Experiment 1

- Procedure and equations
- Model fitting
  - Predict psychometric curves
  - Stability of population level estimates across sessions
  - Stability of subject-level estimates across sessions
  - Within- vs. between-subjects correlation of PSE estimates
  - Additional plots

Start by loading functions and data


```
require(latex2exp)
require(bootES)
require(plyr)
require(tidyverse)
require(assertthat)
df <- read_csv('../data/data.csv')
df$pos <- as.factor(df$pos)
df$session <- as.factor(df$session)
```


# Procedure and equations

We are going to fit a linear mixed effect model to the data. We will model the data as follows

\[
y^k = \text{logit}\left(g(x)\right)\\
g(x) = \beta\_0 x + \sum\_{i=1}^8\left(\beta\_i + z\_i^k\right)I\_i
\] Where \(y^k\) is the response for subject \(k\), \(x\) is the (scaled) percentage of morphing, \(\beta\_i, i=1...4\) are the fixed-effects for each angular location (0 to 315 in 45 deg steps), and \(z\_i^k\) are the random-effects (random slopes for location) for each subject, and \(I\_i\) is an indicator variable, indicating the angular location for each trial.

In this way for each subject we can find the PSE as the point where \(y^k = 0.5\), that is the point \(\hat{x}\)

\[
\text{logit}(g(\hat{x})) = 0.5 \Longleftrightarrow
g(\hat{x}) = 0 \Longleftrightarrow \\
\beta\_0 \hat{x} + \sum\_{i=1}^4\left(\beta\_i + z\_i^k\right)I\_i = 0 \Longleftrightarrow\\
\hat{x} = -\frac{\sum\_{i=1}^4\left(\beta\_i + z\_i^k\right)I\_i}{\beta\_0}
\]

Thus for every angular location \(i\) we have that

\[
\hat{x}\_i = -\frac{\beta\_i}{\beta\_0} - \frac{z\_i^k}{\beta\_0} = \text{PSE}^p\_i + \Delta\text{PSE}\_i^s\\
\]

with \(\text{PSE}\_i^p\) being the population-level PSE at location \(i\), and \(\Delta\text{PSE}\_i^s\) being the change at location \(i\) for subject \(s\).

We will fit one such model for each of the morph types, and one for each session.

# Model fitting


```
require(lme4)
# define some functions
extract_morph_session <- function(df, ses) {
  # Extract trials from one particular  session
  df_ <-
    df %>%
    filter(session == ses) %>%
    mutate(morph_resc=(morph - 50)/100)
  return(df_)
}
run_model_session <- function(df) {
  # Run the following logit mixed-effect model for one session
  # response_bin ~ morph_resc + pos - 1 + (pos - 1 | subject)
  m <- glmer(response_bin ~ morph_resc + pos - 1 + (pos - 1 | subject), 
           data=df, 
           family=binomial(link='logit'), 
           control=glmerControl(optimizer='bobyqa', optCtrl=list(maxfun=100000)))
  return(m)
}
```


Run the model separately for every morph


```
df_ses1 <- extract_morph_session(df, '1')
```


```
package ‘bindrcpp’ was built under R version 3.2.5
```


```
df_ses2 <- extract_morph_session(df, '2')
assert_that(sum(nrow(df_ses1), nrow(df_ses2)) == nrow(df))
```


```
[1] TRUE
```


```
# now compute models
model_ses1 <- run_model_session(df_ses1)
model_ses2 <- run_model_session(df_ses2)
```


The following functions are used to extract both the population \(\text{PSE}^p\) and the subject \(\Delta\text{PSE}^s\). Remember that \(\text{PSE}^s = \text{PSE}^p + \Delta\text{PSE}^s\).


```
population_pse <- function(model) {
  # Computes population-level PSE (see equations above)
  # Note: they are on the scale of morph_resc
  morph_beta <- fixef(model)[1]
  pos_betas <- fixef(model)[-1]
  return(-pos_betas/morph_beta)
}
subjects_pse <- function(model) {
  # Computes subject-level PSE (see equations above)
  morph_pop <- fixef(model)[1]
  position_pop <- fixef(model)[-1]
  ranef_model <- ranef(model)$subject
  # this is the denominator
  morph_subj <- ranef_model[, 1] + morph_pop
  # these are the numerators
  position_subj <- ranef_model[, -1]  
  # extend position_beta to get the same shape as ranef_pos
  position_pop <- matrix(rep(position_pop, nrow(position_subj)), 
                          byrow=T, nrow=nrow(position_subj))
  position_subj <- position_subj + position_pop
  # extend morph_subj to get the same shape as position_subj
  morph_subj <- matrix(rep(morph_subj, ncol(position_subj)), ncol=ncol(position_subj)) 
  return(-position_subj/morph_subj)
}
delta_pse <- function(model) {
  # Computes subject-level PSE (see equations above)
  # Note: they are on the scale of morph_resc
  # IF 'morph_resc' is entered as a random effect, add that for each individual
  # morph
  morph_beta <- fixef(model)[1]
  ranef_model <- ranef(model)$subject
  if ('morph_resc' %in% names(ranef_model)) {
    pse_pop <- population_pse(model)
    pse_subj <- subjects_pse(model)
    pse_pop <- matrix(rep(pse_pop, nrow(pse_subj)), nrow=nrow(pse_subj), byrow=T)
    return(pse_subj - pse_pop) 
  } else {
    return(-ranef_model/morph_beta)
  }
}
```


Let’s look at the population estimates for the PSEs across the two sessions, as well as the subject-level estimates.


```
psep_ses1 <- population_pse(model_ses1)
psep_ses2 <- population_pse(model_ses2)
dpse_ses1 <- delta_pse(model_ses1)
dpse_ses2 <- delta_pse(model_ses2)
```


## Predict psychometric curves

Let’s plot the population estimates first


```
df_predict <-
  expand.grid(morph_resc=seq(-0.5, 0.5, 0.01), pos=unique(df$pos))
predict_pop_ses1 <- sapply(list(model_ses1), predict, newdata=df_predict, 
                           re.form=NA, type='response', simplify=F, USE.NAMES=T)
predict_pop_ses2 <- sapply(list(model_ses2), predict, newdata=df_predict, 
                           re.form=NA, type='response', simplify=F, USE.NAMES=T)
# add df_predict to each of them
predict_pop_ses1 <- lapply(predict_pop_ses1, function(x) cbind(df_predict, pred=x))
predict_pop_ses2 <- lapply(predict_pop_ses2, function(x) cbind(df_predict, pred=x))
predict_pop_ses1 <- ldply(predict_pop_ses1, data.frame)
predict_pop_ses2 <- ldply(predict_pop_ses2, data.frame)
predict_pop_ses1$session <- '1'
predict_pop_ses2$session <- '2'
predict_pop <- rbind(predict_pop_ses1, predict_pop_ses2)
predict_pop <- 
  predict_pop %>%
  mutate(morph=morph_resc*100  + 50, 
         pos_num=as.numeric(as.character(pos))*45)
```


```
# modified from https://rpubs.com/Koundy/71792
theme_Publication <- function(base_size=12) {
      library(ggthemes)
      (theme_foundation(base_size=base_size)
       + theme(plot.title = element_text(face = "bold",
                                         size = rel(1.2), hjust = 0.5),
               text = element_text(),
               panel.background = element_rect(colour = NA),
               plot.background = element_rect(colour = NA),
               panel.border = element_rect(colour = NA),
               axis.title = element_text(size = rel(1)),
               axis.title.y = element_text(angle=90,vjust =2),
               axis.title.x = element_text(vjust = -0.2),
               axis.text = element_text(), 
               axis.line = element_line(colour="black"),
               axis.ticks = element_line(),
               panel.grid.major = element_blank(),#element_line(colour="#f0f0f0"),
               panel.grid.minor = element_blank(),
               legend.key = element_rect(colour = NA),
               legend.position = "bottom",
               legend.direction = "horizontal",
               #legend.key.size= unit(0.2, "cm"),
               legend.spacing = unit(0, "cm"),
               legend.title = element_text(),
               plot.margin = unit(c(10,5,5,5),"mm"),
               strip.background = element_rect(colour="#f0f0f0",fill="#f0f0f0"),
               strip.text = element_text(face="bold"),
               strip.text.y = element_text(angle = 0)
          ))
      
}
```


```
df <-
  df %>%
  mutate(pos_num=as.numeric(as.character(pos))*45)
predict_pop %>%
  ggplot(aes(morph, pred*100, color=session)) +
  geom_segment(x=-10, xend=110, y=50, yend=50, size=0.1, color='lightgray', alpha=0.5, linetype='dashed') +
  geom_segment(x=50, xend=50, y=-10, yend=110, size=0.1, color='lightgray', alpha=0.5, linetype='dashed') +
  geom_line() +
  # add individual data
  stat_summary(data=df, 
               aes(morph, response_bin), 
               fun.y=function(x) sum(x)/length(x)*100, geom='point') +
  facet_wrap(~ pos_num, ncol=4) +
  labs(x='Percentage morphing', y='Population prediction\npercent responses to second identity', color='Session') +
  scale_color_brewer(palette='Set1') +
  theme_Publication() + 
  coord_equal()
```


```
package ‘ggthemes’ was built under R version 3.2.5
```


```
  #scale_x_continuous(breaks=c(0, 17, 33, 50, 67, 83, 100))
ggsave('../img/pred_pop_gmm.png', width=8, height=6)
```


Now we can predict for each individual subject


```
df_predict <-
  expand.grid(
    morph_resc=seq(-0.5, 0.5, 0.01), 
    pos=unique(df$pos),
    subject=unique(df$subject))
predict_subj_ses1 <- sapply(list(model_ses1), predict, newdata=df_predict, 
                           type='response', simplify=F, USE.NAMES=T)
predict_subj_ses2 <- sapply(list(model_ses2), predict, newdata=df_predict, 
                           type='response', simplify=F, USE.NAMES=T)
# add df_predict to each of them
predict_subj_ses1 <- lapply(predict_subj_ses1, function(x) cbind(df_predict, pred=x))
predict_subj_ses2 <- lapply(predict_subj_ses2, function(x) cbind(df_predict, pred=x))
predict_subj_ses1 <- ldply(predict_subj_ses1, data.frame)
predict_subj_ses2 <- ldply(predict_subj_ses2, data.frame)
predict_subj_ses1$session <- '1'
predict_subj_ses2$session <- '2'
predict_subj <- rbind(predict_subj_ses1, predict_subj_ses2)
predict_subj <- 
  predict_subj %>%
  mutate(morph=morph_resc*100  + 50, 
         pos_num=as.numeric(as.character(pos))*45)
```


Now we can save each individual plot to disk


```
subjects <- unique(df$subject)
for (subj in subjects) {
  out_dir <- file.path('../img', 'pred_gmm')
  fnout <- file.path(out_dir, paste(subj, '_pred_gmm.png', sep=''))
  # setup dataframes for plotting
  this_subject_df <- df %>% 
    filter(subject == subj) %>%
    # add position in angles
    mutate(pos_num=as.numeric(as.character(pos))*45)
  this_predict_subj <- predict_subj %>% 
    filter(subject == subj) %>%
    # add position in angles
    mutate(pos_num=as.numeric(as.character(pos))*45)
  
  plot_curve <-
    this_predict_subj %>%
    # add prediction
    ggplot(aes(morph, pred*100, color=session)) +
    geom_segment(x=-10, xend=110, y=50, yend=50, size=0.1, color='lightgray', alpha=0.5, linetype='dashed') +
    geom_segment(x=50, xend=50, y=-10, yend=110, size=0.1, color='lightgray', alpha=0.5, linetype='dashed') +
    geom_line() +
    # add individual data
    stat_summary(data=this_subject_df, 
                 aes(morph, response_bin), 
                 fun.y=function(x) sum(x)/length(x)*100, geom='point') +
    facet_wrap(~pos_num, ncol=4) +
    labs(x='Percentage morphing', y='Percent responses to second identity', color='Session') +
    scale_color_brewer(palette='Set1') +
    ggtitle(paste("Subject", subj)) +
    theme_Publication() +
    coord_equal() #+
    #scale_x_continuous(breaks=c(0, 17, 33, 50, 67, 83, 100))
  
    # save
    dir.create(out_dir, recursive=T)
    ggsave(filename=fnout, plot=plot_curve, width=8, height=6)
}
```


```
'../img/pred_gmm' already exists
```

## Stability of population level estimates across sessions

These are the population-level estimates


Let’s compute a correlation across sessions


```
psep_wide <- 
psep %>% 
  spread(session, pse) %>% 
  mutate(session1=.$'1', session2=.$'2')
ggplot(psep_wide, aes(session1*100 + 50, session2*100 + 50, group=1)) + 
  geom_smooth(method='lm', color='darkgray', se=F) +
  geom_point() +
  labs(x='First measurement (PSE)', y='Second measurement (PSE)', shape='Morph') +
  theme_Publication()
```


```
  #coord_equal() +
  #scale_x_continuous(breaks=round(psep_wide$session1*100 + 50)) +
  #scale_y_continuous(breaks=round(psep_wide$session2*100 + 50))
ggsave('../img/pse_pop_scatter.png', width=5, height=5)
```


And these are the correlation values


```
set.seed(234)
bootES(psep_wide[c('session1', 'session2')], R=2000)
```


```
95.00% bca Confidence Interval, 2000 replicates
Stat        CI (Low)    CI (High)   bias        SE          
0.893       -0.233      0.996       -0.115      0.317
```


```
cor.test(psep_wide$session1, psep_wide$session2)
```


```
    Pearson's product-moment correlation

data:  psep_wide$session1 and psep_wide$session2
t = 4.8572, df = 6, p-value = 0.002831
alternative hypothesis: true correlation is not equal to 0
95 percent confidence interval:
 0.5075767 0.9805832
sample estimates:
      cor 
0.8928861
```

## Stability of subject-level estimates across sessions

Let’s also look at the \(\Delta\text{PSE}\)


```
# get dpse in long format for plotting
dpse_ses1_long <- 
  dpse_ses1 %>%
  mutate(subject=row.names(.)) %>%
  gather(pos, pse, -subject) %>%
  mutate(session='1')
dpse_ses2_long <- 
  dpse_ses2 %>%
  mutate(subject=row.names(.)) %>%
  gather(pos, pse, -subject) %>%
  mutate(session='2')
dpse <- 
  rbind(dpse_ses1_long, dpse_ses2_long) %>%
  mutate(pos_num=mapvalues(pos, 
                           paste('pos', 0:7, sep=''), 
                           seq(0, 7)*45))
dpse$pos_num <- factor(dpse$pos_num, levels=seq(0, 7)*45)
```


```
dpse_wide <-
dpse %>%
  spread(session, pse) %>%
  mutate(session1=.$'1', session2=.$'2')
ggplot(dpse_wide, aes(session1*100, session2*100, color=subject)) + 
  geom_smooth(method='lm', color='darkgray', se=F) +
  geom_point() +
  labs(x=TeX('First measurement ($\\Delta$PSE)'), 
       y=TeX('Second measurement ($\\Delta$PSE)'), 
       color='Subject') +
  theme_Publication() +
  coord_equal() +
  #guides(color=F) +
  scale_color_brewer(palette='Set1') +
  theme(legend.position=c(0.95, 0.15),
        legend.direction='vertical',
        legend.key.size=unit(.8, 'picas'),
        legend.title=element_text(size=10))
```


```
  #coord_equal(xlim=c(-20, 20), ylim=c(-20, 20))
  #scale_x_continuous(breaks=seq(-60, 40, 20)) +
  #scale_y_continuous(breaks=seq(-60, 40, 20))
ggsave('../img/pse_subj_scatter.png', width=5, height=5)
```


And correlation values as well


```
set.seed(324)
bootES(dpse_wide[c('session1', 'session2')], R=10000)
```


```
95.00% bca Confidence Interval, 10000 replicates
Stat        CI (Low)    CI (High)   bias        SE          
0.707       0.464       0.842       -0.004      0.092
```


```
cor.test(dpse_wide$session1, dpse_wide$session2)
```


```
    Pearson's product-moment correlation

data:  dpse_wide$session1 and dpse_wide$session2
t = 5.4743, df = 30, p-value = 6.106e-06
alternative hypothesis: true correlation is not equal to 0
95 percent confidence interval:
 0.4754108 0.8468616
sample estimates:
      cor 
0.7069171
```

## Within- vs. between-subjects correlation of PSE estimates

Let’s compute the correlation between the first and the second session to compare within vs. between-subject correlations


```
this_cor <- cor(t(dpse_ses1), t(dpse_ses2))
# make it symmetric
this_cor <- (this_cor + t(this_cor))/2.
cor_ses12 <- this_cor
# make a dataframe in long format
make_cor_long <- function(cor) {
  within <- diag(cor)
  between <- cor[lower.tri(cor)]
  
  df_within_between <- data.frame(corr=c(within, between),
                                  type=c(rep('within', length(within)),
                                         rep('between', length(between)))
                                  )
  return(df_within_between)
}
cor_ses12_long <- make_cor_long(cor_ses12)
```


We can check whether the estimates are consistent across sessions, and also subject-specific, by comparing the within-subject correlations with the between-subject correlations. We will compute the bootstrapped difference \(\text{Within} - \text{Between}\).


```
ggplot(data=boot_dist_df, aes(1, t)) +
  geom_violin(adjust=2) +
  geom_errorbar(data=boot_cis_df, aes(ymin=lci, ymax=rci, y=t0), width=0.01) + 
  geom_point(data=boot_cis_df, aes(y=t0)) +
  geom_hline(yintercept=0, linetype='dashed') +
  labs(x='Morph', y='Within - Between subject correlations') +
  theme_Publication() +
  coord_flip() + 
  theme(aspect.ratio=3/4)
```


```
Ignoring unknown aesthetics: y
```


And finally plot them


```
ggplot(data=boot_dist_df, aes(1, t)) +
  geom_violin(adjust=2) +
  geom_errorbar(data=boot_cis_df, aes(ymin=lci, ymax=rci, y=t0), width=0.01) + 
  geom_point(data=boot_cis_df, aes(y=t0)) +
  geom_hline(yintercept=0, linetype='dashed') +
  labs(x='Morph', y='Within - Between subject correlations') +
  theme_Publication() +
  coord_flip() + 
  theme(aspect.ratio=3/4)
```


```
Ignoring unknown aesthetics: y
```


And these are the values


```
round(boot_cis_df, 2)
```


Compute also for within and between


```
set.seed(4254)
cor_ses12_long %>% filter(type == 'within') %>% bootES(data.col='corr', R=10000)
```


```
95.00% bca Confidence Interval, 10000 replicates
Stat        CI (Low)    CI (High)   bias        SE          
0.651       0.565       0.795       0.000       0.060
```


```
set.seed(4254)
cor_ses12_long %>% filter(type == 'between') %>% bootES(data.col='corr', R=10000)
```


```
95.00% bca Confidence Interval, 10000 replicates
Stat        CI (Low)    CI (High)   bias        SE          
-0.216      -0.406      -0.011      -0.002      0.101
```

## Additional plots

Let’s make some plots to show the difference in psychometric curves for each subject


```
pse_subj <- list()
pse_subj[[1]] <-  dpse_ses1 + matrix(rep(psep_ses1, nrow(dpse_ses1)), 
                                     byrow=T, nrow=nrow(dpse_ses1)) 
pse_subj[[2]] <-  dpse_ses2 + matrix(rep(psep_ses2, nrow(dpse_ses2)), 
                                     byrow=T, nrow=nrow(dpse_ses2)) 
plot_examplefit <- function(which_subj, ses=2, extreme_curves=NULL) {
  pse_subj_ses  <- pse_subj[[ses]]
  
  pse_subj_ses <-
  pse_subj_ses %>%
    mutate(subject=row.names(.)) %>%
    gather(pos, pse, -subject) %>%
    mutate(pse=pse*100 + 50,
      pos_num=mapvalues(
              pos, 
              paste('pos', seq(0, 7), sep=''), 
              seq(0, 7)*45),
      pos=mapvalues(
              pos, 
              paste('pos', seq(0, 7), sep=''), 
              seq(0, 7))) %>%
    filter(subject == which_subj)
  
  predict_subj$pos_num <-
    factor(predict_subj$pos_num, levels=seq(0, 7)*45)
  pse_subj_ses$pos_num <-
    factor(pse_subj_ses$pos_num, levels=seq(0, 7)*45)
  
  df_plot <- df %>%
    filter(subject == which_subj, session == as.character(ses)) 
  df_plot$pos_num <- 
    factor(df_plot$pos_num, levels=seq(0, 7)*45)
  
  # these are the extreme angular locations for the two subjects we'll plot
  if (!is.null(extreme_curves)) {
    df_plot <-
    df_plot %>%
      filter(pos_num %in% extreme_curves)
  }
  
  # plot
  plot <-
  predict_subj %>%
    filter(subject == which_subj, session == as.character(ses)) %>%
    ggplot(aes((morph_resc*100)+50, pred*100, color=pos_num)) +
    geom_segment(aes(x=pse, xend=pse, y=-10, yend=50), alpha=0.8, linetype='dashed', data=pse_subj_ses) +
    geom_line(size=0.8) +
    theme_Publication() +
    coord_equal(ylim=c(-0.4, 101)) +
    labs(x='Percentage morphing', y='Percentage responses\nto second identity', color='Angular location')
  
  if (!is.null(extreme_curves)) {
      plot <- plot +
      stat_summary(fun.y=function(x) sum(x)/length(x)*100, 
                   aes(morph, response_bin), data=df_plot, geom='point',
                   size=1.2, show.legend=F) 
  }
  return(plot)
  }
```


```
extreme_values <- list(
  s01=c('90', '270'),
  s02=c('90', '315'),
  s03=c('90', '225'),
  s04=c('0', '270')
)
```


```
for (ses in 1:2) {
  for (s in subjects) {
    plot <- plot_examplefit(s, ses, extreme_values[[s]])
    ggsave(paste('../img/example_fit_', s, '_ses', ses, '.png', sep=''),
           width=5, height=5)
  }
}
```

LS0tCnRpdGxlOiAiRXhwZXJpbWVudCAxIgpvdXRwdXQ6CiAgcGRmX2RvY3VtZW50OgogICAgdG9jOiB5ZXMKICBodG1sX25vdGVib29rOgogICAgdG9jOiB5ZXMKLS0tCgpTdGFydCBieSBsb2FkaW5nIGZ1bmN0aW9ucyBhbmQgZGF0YQpgYGB7ciwgbWVzc2FnZT1GQUxTRSwgd2FybmluZz1GQUxTRX0KcmVxdWlyZShsYXRleDJleHApCnJlcXVpcmUoYm9vdEVTKQpyZXF1aXJlKHBseXIpCnJlcXVpcmUodGlkeXZlcnNlKQpyZXF1aXJlKGFzc2VydHRoYXQpCmRmIDwtIHJlYWRfY3N2KCcuLi9kYXRhL2RhdGEuY3N2JykKZGYkcG9zIDwtIGFzLmZhY3RvcihkZiRwb3MpCmRmJHNlc3Npb24gPC0gYXMuZmFjdG9yKGRmJHNlc3Npb24pCmBgYAoKIyBQcm9jZWR1cmUgYW5kIGVxdWF0aW9ucwoKV2UgYXJlIGdvaW5nIHRvIGZpdCBhIGxpbmVhciBtaXhlZCBlZmZlY3QgbW9kZWwgdG8gdGhlIGRhdGEuIFdlIHdpbGwgbW9kZWwgdGhlIGRhdGEgYXMgZm9sbG93cwoKJCQKeV5rID0gXHRleHR7bG9naXR9XGxlZnQoZyh4KVxyaWdodClcXApnKHgpID0gXGJldGFfMCB4ICsgXHN1bV97aT0xfV44XGxlZnQoXGJldGFfaSArIHpfaV5rXHJpZ2h0KUlfaQokJApXaGVyZSAkeV5rJCBpcyB0aGUgcmVzcG9uc2UgZm9yIHN1YmplY3QgJGskLCAkeCQgaXMgdGhlIChzY2FsZWQpIHBlcmNlbnRhZ2Ugb2YgbW9ycGhpbmcsCiRcYmV0YV9pLCBpPTEuLi40JCBhcmUgdGhlIGZpeGVkLWVmZmVjdHMgZm9yIGVhY2ggYW5ndWxhciBsb2NhdGlvbiAoMCB0byAzMTUgaW4gNDUgZGVnIHN0ZXBzKSwgCmFuZCAkel9pXmskIGFyZSB0aGUgcmFuZG9tLWVmZmVjdHMgKHJhbmRvbSBzbG9wZXMgZm9yIGxvY2F0aW9uKSAKZm9yIGVhY2ggc3ViamVjdCwgYW5kICRJX2kkIGlzIGFuIGluZGljYXRvciB2YXJpYWJsZSwgaW5kaWNhdGluZyB0aGUgYW5ndWxhciBsb2NhdGlvbiBmb3IgZWFjaCB0cmlhbC4KCkluIHRoaXMgd2F5IGZvciBlYWNoIHN1YmplY3Qgd2UgY2FuIGZpbmQgdGhlIFBTRSBhcyB0aGUgcG9pbnQgd2hlcmUgJHleayA9IDAuNSQsIHRoYXQgaXMgdGhlIHBvaW50ICRcaGF0e3h9JAoKJCQKXHRleHR7bG9naXR9KGcoXGhhdHt4fSkpID0gMC41IFxMb25nbGVmdHJpZ2h0YXJyb3cKZyhcaGF0e3h9KSA9IDAgXExvbmdsZWZ0cmlnaHRhcnJvdyBcXApcYmV0YV8wIFxoYXR7eH0gKyBcc3VtX3tpPTF9XjRcbGVmdChcYmV0YV9pICsgel9pXmtccmlnaHQpSV9pID0gMCBcTG9uZ2xlZnRyaWdodGFycm93XFwKXGhhdHt4fSA9IC1cZnJhY3tcc3VtX3tpPTF9XjRcbGVmdChcYmV0YV9pICsgel9pXmtccmlnaHQpSV9pfXtcYmV0YV8wfQokJAoKVGh1cyBmb3IgZXZlcnkgYW5ndWxhciBsb2NhdGlvbiAkaSQgd2UgaGF2ZSB0aGF0IAoKJCQKXGhhdHt4fV9pID0gLVxmcmFje1xiZXRhX2l9e1xiZXRhXzB9IC0gXGZyYWN7el9pXmt9e1xiZXRhXzB9ID0gXHRleHR7UFNFfV5wX2kgKyBcRGVsdGFcdGV4dHtQU0V9X2lec1xcCiQkCgp3aXRoICRcdGV4dHtQU0V9X2lecCQgYmVpbmcgdGhlIHBvcHVsYXRpb24tbGV2ZWwgUFNFCmF0IGxvY2F0aW9uICRpJCwgYW5kICRcRGVsdGFcdGV4dHtQU0V9X2lecyQgYmVpbmcgdGhlIGNoYW5nZSBhdCBsb2NhdGlvbiAkaSQKZm9yIHN1YmplY3QgJHMkLiAKCldlIHdpbGwgZml0IG9uZSBzdWNoIG1vZGVsIGZvciBlYWNoIG9mIHRoZSBtb3JwaCB0eXBlcywgYW5kIG9uZQpmb3IgZWFjaCBzZXNzaW9uLgoKIyBNb2RlbCBmaXR0aW5nCmBgYHtyLCBtZXNzYWdlPUZBTFNFLCB3YXJuaW5nPUZBTFNFfQpyZXF1aXJlKGxtZTQpCgojIGRlZmluZSBzb21lIGZ1bmN0aW9ucwpleHRyYWN0X21vcnBoX3Nlc3Npb24gPC0gZnVuY3Rpb24oZGYsIHNlcykgewogICMgRXh0cmFjdCB0cmlhbHMgZnJvbSBvbmUgcGFydGljdWxhciAgc2Vzc2lvbgogIGRmXyA8LQogICAgZGYgJT4lCiAgICBmaWx0ZXIoc2Vzc2lvbiA9PSBzZXMpICU+JQogICAgbXV0YXRlKG1vcnBoX3Jlc2M9KG1vcnBoIC0gNTApLzEwMCkKICByZXR1cm4oZGZfKQp9CnJ1bl9tb2RlbF9zZXNzaW9uIDwtIGZ1bmN0aW9uKGRmKSB7CiAgIyBSdW4gdGhlIGZvbGxvd2luZyBsb2dpdCBtaXhlZC1lZmZlY3QgbW9kZWwgZm9yIG9uZSBzZXNzaW9uCiAgIyByZXNwb25zZV9iaW4gfiBtb3JwaF9yZXNjICsgcG9zIC0gMSArIChwb3MgLSAxIHwgc3ViamVjdCkKICBtIDwtIGdsbWVyKHJlc3BvbnNlX2JpbiB+IG1vcnBoX3Jlc2MgKyBwb3MgLSAxICsgKHBvcyAtIDEgfCBzdWJqZWN0KSwgCiAgICAgICAgICAgZGF0YT1kZiwgCiAgICAgICAgICAgZmFtaWx5PWJpbm9taWFsKGxpbms9J2xvZ2l0JyksIAogICAgICAgICAgIGNvbnRyb2w9Z2xtZXJDb250cm9sKG9wdGltaXplcj0nYm9ieXFhJywgb3B0Q3RybD1saXN0KG1heGZ1bj0xMDAwMDApKSkKICByZXR1cm4obSkKfQpgYGAKClJ1biB0aGUgbW9kZWwgc2VwYXJhdGVseSBmb3IgZXZlcnkgbW9ycGgKYGBge3J9CmRmX3NlczEgPC0gZXh0cmFjdF9tb3JwaF9zZXNzaW9uKGRmLCAnMScpCmRmX3NlczIgPC0gZXh0cmFjdF9tb3JwaF9zZXNzaW9uKGRmLCAnMicpCmFzc2VydF90aGF0KHN1bShucm93KGRmX3NlczEpLCBucm93KGRmX3NlczIpKSA9PSBucm93KGRmKSkKIyBub3cgY29tcHV0ZSBtb2RlbHMKbW9kZWxfc2VzMSA8LSBydW5fbW9kZWxfc2Vzc2lvbihkZl9zZXMxKQptb2RlbF9zZXMyIDwtIHJ1bl9tb2RlbF9zZXNzaW9uKGRmX3NlczIpCmBgYAoKVGhlIGZvbGxvd2luZyBmdW5jdGlvbnMgYXJlIHVzZWQgdG8gZXh0cmFjdCBib3RoIHRoZSBwb3B1bGF0aW9uICRcdGV4dHtQU0V9XnAkIGFuZCB0aGUgc3ViamVjdCAkXERlbHRhXHRleHR7UFNFfV5zJC4gClJlbWVtYmVyIHRoYXQgJFx0ZXh0e1BTRX1ecyA9IFx0ZXh0e1BTRX1ecCArIFxEZWx0YVx0ZXh0e1BTRX1ecyQuCmBgYHtyfQpwb3B1bGF0aW9uX3BzZSA8LSBmdW5jdGlvbihtb2RlbCkgewogICMgQ29tcHV0ZXMgcG9wdWxhdGlvbi1sZXZlbCBQU0UgKHNlZSBlcXVhdGlvbnMgYWJvdmUpCiAgIyBOb3RlOiB0aGV5IGFyZSBvbiB0aGUgc2NhbGUgb2YgbW9ycGhfcmVzYwogIG1vcnBoX2JldGEgPC0gZml4ZWYobW9kZWwpWzFdCiAgcG9zX2JldGFzIDwtIGZpeGVmKG1vZGVsKVstMV0KICByZXR1cm4oLXBvc19iZXRhcy9tb3JwaF9iZXRhKQp9CgpzdWJqZWN0c19wc2UgPC0gZnVuY3Rpb24obW9kZWwpIHsKICAjIENvbXB1dGVzIHN1YmplY3QtbGV2ZWwgUFNFIChzZWUgZXF1YXRpb25zIGFib3ZlKQogIG1vcnBoX3BvcCA8LSBmaXhlZihtb2RlbClbMV0KICBwb3NpdGlvbl9wb3AgPC0gZml4ZWYobW9kZWwpWy0xXQogIHJhbmVmX21vZGVsIDwtIHJhbmVmKG1vZGVsKSRzdWJqZWN0CiAgIyB0aGlzIGlzIHRoZSBkZW5vbWluYXRvcgogIG1vcnBoX3N1YmogPC0gcmFuZWZfbW9kZWxbLCAxXSArIG1vcnBoX3BvcAogICMgdGhlc2UgYXJlIHRoZSBudW1lcmF0b3JzCiAgcG9zaXRpb25fc3ViaiA8LSByYW5lZl9tb2RlbFssIC0xXSAgCiAgIyBleHRlbmQgcG9zaXRpb25fYmV0YSB0byBnZXQgdGhlIHNhbWUgc2hhcGUgYXMgcmFuZWZfcG9zCiAgcG9zaXRpb25fcG9wIDwtIG1hdHJpeChyZXAocG9zaXRpb25fcG9wLCBucm93KHBvc2l0aW9uX3N1YmopKSwgCiAgICAgICAgICAgICAgICAgICAgICAgICAgYnlyb3c9VCwgbnJvdz1ucm93KHBvc2l0aW9uX3N1YmopKQogIHBvc2l0aW9uX3N1YmogPC0gcG9zaXRpb25fc3ViaiArIHBvc2l0aW9uX3BvcAogICMgZXh0ZW5kIG1vcnBoX3N1YmogdG8gZ2V0IHRoZSBzYW1lIHNoYXBlIGFzIHBvc2l0aW9uX3N1YmoKICBtb3JwaF9zdWJqIDwtIG1hdHJpeChyZXAobW9ycGhfc3ViaiwgbmNvbChwb3NpdGlvbl9zdWJqKSksIG5jb2w9bmNvbChwb3NpdGlvbl9zdWJqKSkgCiAgcmV0dXJuKC1wb3NpdGlvbl9zdWJqL21vcnBoX3N1YmopCn0KCmRlbHRhX3BzZSA8LSBmdW5jdGlvbihtb2RlbCkgewogICMgQ29tcHV0ZXMgc3ViamVjdC1sZXZlbCBQU0UgKHNlZSBlcXVhdGlvbnMgYWJvdmUpCiAgIyBOb3RlOiB0aGV5IGFyZSBvbiB0aGUgc2NhbGUgb2YgbW9ycGhfcmVzYwogICMgSUYgJ21vcnBoX3Jlc2MnIGlzIGVudGVyZWQgYXMgYSByYW5kb20gZWZmZWN0LCBhZGQgdGhhdCBmb3IgZWFjaCBpbmRpdmlkdWFsCiAgIyBtb3JwaAogIG1vcnBoX2JldGEgPC0gZml4ZWYobW9kZWwpWzFdCiAgcmFuZWZfbW9kZWwgPC0gcmFuZWYobW9kZWwpJHN1YmplY3QKICBpZiAoJ21vcnBoX3Jlc2MnICVpbiUgbmFtZXMocmFuZWZfbW9kZWwpKSB7CiAgICBwc2VfcG9wIDwtIHBvcHVsYXRpb25fcHNlKG1vZGVsKQogICAgcHNlX3N1YmogPC0gc3ViamVjdHNfcHNlKG1vZGVsKQogICAgcHNlX3BvcCA8LSBtYXRyaXgocmVwKHBzZV9wb3AsIG5yb3cocHNlX3N1YmopKSwgbnJvdz1ucm93KHBzZV9zdWJqKSwgYnlyb3c9VCkKICAgIHJldHVybihwc2Vfc3ViaiAtIHBzZV9wb3ApIAogIH0gZWxzZSB7CiAgICByZXR1cm4oLXJhbmVmX21vZGVsL21vcnBoX2JldGEpCiAgfQp9CmBgYAoKTGV0J3MgbG9vayBhdCB0aGUgcG9wdWxhdGlvbiBlc3RpbWF0ZXMgZm9yIHRoZSBQU0VzIGFjcm9zcyB0aGUgdHdvCnNlc3Npb25zLCBhcyB3ZWxsIGFzIHRoZSBzdWJqZWN0LWxldmVsIGVzdGltYXRlcy4KCmBgYHtyfQpwc2VwX3NlczEgPC0gcG9wdWxhdGlvbl9wc2UobW9kZWxfc2VzMSkKcHNlcF9zZXMyIDwtIHBvcHVsYXRpb25fcHNlKG1vZGVsX3NlczIpCmRwc2Vfc2VzMSA8LSBkZWx0YV9wc2UobW9kZWxfc2VzMSkKZHBzZV9zZXMyIDwtIGRlbHRhX3BzZShtb2RlbF9zZXMyKQpgYGAKCiMjIFByZWRpY3QgcHN5Y2hvbWV0cmljIGN1cnZlcwpMZXQncyBwbG90IHRoZSBwb3B1bGF0aW9uIGVzdGltYXRlcyBmaXJzdApgYGB7cn0KZGZfcHJlZGljdCA8LQogIGV4cGFuZC5ncmlkKG1vcnBoX3Jlc2M9c2VxKC0wLjUsIDAuNSwgMC4wMSksIHBvcz11bmlxdWUoZGYkcG9zKSkKCnByZWRpY3RfcG9wX3NlczEgPC0gc2FwcGx5KGxpc3QobW9kZWxfc2VzMSksIHByZWRpY3QsIG5ld2RhdGE9ZGZfcHJlZGljdCwgCiAgICAgICAgICAgICAgICAgICAgICAgICAgIHJlLmZvcm09TkEsIHR5cGU9J3Jlc3BvbnNlJywgc2ltcGxpZnk9RiwgVVNFLk5BTUVTPVQpCnByZWRpY3RfcG9wX3NlczIgPC0gc2FwcGx5KGxpc3QobW9kZWxfc2VzMiksIHByZWRpY3QsIG5ld2RhdGE9ZGZfcHJlZGljdCwgCiAgICAgICAgICAgICAgICAgICAgICAgICAgIHJlLmZvcm09TkEsIHR5cGU9J3Jlc3BvbnNlJywgc2ltcGxpZnk9RiwgVVNFLk5BTUVTPVQpCgojIGFkZCBkZl9wcmVkaWN0IHRvIGVhY2ggb2YgdGhlbQpwcmVkaWN0X3BvcF9zZXMxIDwtIGxhcHBseShwcmVkaWN0X3BvcF9zZXMxLCBmdW5jdGlvbih4KSBjYmluZChkZl9wcmVkaWN0LCBwcmVkPXgpKQpwcmVkaWN0X3BvcF9zZXMyIDwtIGxhcHBseShwcmVkaWN0X3BvcF9zZXMyLCBmdW5jdGlvbih4KSBjYmluZChkZl9wcmVkaWN0LCBwcmVkPXgpKQoKcHJlZGljdF9wb3Bfc2VzMSA8LSBsZHBseShwcmVkaWN0X3BvcF9zZXMxLCBkYXRhLmZyYW1lKQpwcmVkaWN0X3BvcF9zZXMyIDwtIGxkcGx5KHByZWRpY3RfcG9wX3NlczIsIGRhdGEuZnJhbWUpCnByZWRpY3RfcG9wX3NlczEkc2Vzc2lvbiA8LSAnMScKcHJlZGljdF9wb3Bfc2VzMiRzZXNzaW9uIDwtICcyJwoKcHJlZGljdF9wb3AgPC0gcmJpbmQocHJlZGljdF9wb3Bfc2VzMSwgcHJlZGljdF9wb3Bfc2VzMikKcHJlZGljdF9wb3AgPC0gCiAgcHJlZGljdF9wb3AgJT4lCiAgbXV0YXRlKG1vcnBoPW1vcnBoX3Jlc2MqMTAwICArIDUwLCAKICAgICAgICAgcG9zX251bT1hcy5udW1lcmljKGFzLmNoYXJhY3Rlcihwb3MpKSo0NSkKYGBgCgpgYGB7cn0KIyBtb2RpZmllZCBmcm9tIGh0dHBzOi8vcnB1YnMuY29tL0tvdW5keS83MTc5Mgp0aGVtZV9QdWJsaWNhdGlvbiA8LSBmdW5jdGlvbihiYXNlX3NpemU9MTIpIHsKICAgICAgbGlicmFyeShnZ3RoZW1lcykKICAgICAgKHRoZW1lX2ZvdW5kYXRpb24oYmFzZV9zaXplPWJhc2Vfc2l6ZSkKICAgICAgICsgdGhlbWUocGxvdC50aXRsZSA9IGVsZW1lbnRfdGV4dChmYWNlID0gImJvbGQiLAogICAgICAgICAgICAgICAgICAgICAgICAgICAgICAgICAgICAgICAgIHNpemUgPSByZWwoMS4yKSwgaGp1c3QgPSAwLjUpLAogICAgICAgICAgICAgICB0ZXh0ID0gZWxlbWVudF90ZXh0KCksCiAgICAgICAgICAgICAgIHBhbmVsLmJhY2tncm91bmQgPSBlbGVtZW50X3JlY3QoY29sb3VyID0gTkEpLAogICAgICAgICAgICAgICBwbG90LmJhY2tncm91bmQgPSBlbGVtZW50X3JlY3QoY29sb3VyID0gTkEpLAogICAgICAgICAgICAgICBwYW5lbC5ib3JkZXIgPSBlbGVtZW50X3JlY3QoY29sb3VyID0gTkEpLAogICAgICAgICAgICAgICBheGlzLnRpdGxlID0gZWxlbWVudF90ZXh0KHNpemUgPSByZWwoMSkpLAogICAgICAgICAgICAgICBheGlzLnRpdGxlLnkgPSBlbGVtZW50X3RleHQoYW5nbGU9OTAsdmp1c3QgPTIpLAogICAgICAgICAgICAgICBheGlzLnRpdGxlLnggPSBlbGVtZW50X3RleHQodmp1c3QgPSAtMC4yKSwKICAgICAgICAgICAgICAgYXhpcy50ZXh0ID0gZWxlbWVudF90ZXh0KCksIAogICAgICAgICAgICAgICBheGlzLmxpbmUgPSBlbGVtZW50X2xpbmUoY29sb3VyPSJibGFjayIpLAogICAgICAgICAgICAgICBheGlzLnRpY2tzID0gZWxlbWVudF9saW5lKCksCiAgICAgICAgICAgICAgIHBhbmVsLmdyaWQubWFqb3IgPSBlbGVtZW50X2JsYW5rKCksI2VsZW1lbnRfbGluZShjb2xvdXI9IiNmMGYwZjAiKSwKICAgICAgICAgICAgICAgcGFuZWwuZ3JpZC5taW5vciA9IGVsZW1lbnRfYmxhbmsoKSwKICAgICAgICAgICAgICAgbGVnZW5kLmtleSA9IGVsZW1lbnRfcmVjdChjb2xvdXIgPSBOQSksCiAgICAgICAgICAgICAgIGxlZ2VuZC5wb3NpdGlvbiA9ICJib3R0b20iLAogICAgICAgICAgICAgICBsZWdlbmQuZGlyZWN0aW9uID0gImhvcml6b250YWwiLAogICAgICAgICAgICAgICAjbGVnZW5kLmtleS5zaXplPSB1bml0KDAuMiwgImNtIiksCiAgICAgICAgICAgICAgIGxlZ2VuZC5zcGFjaW5nID0gdW5pdCgwLCAiY20iKSwKICAgICAgICAgICAgICAgbGVnZW5kLnRpdGxlID0gZWxlbWVudF90ZXh0KCksCiAgICAgICAgICAgICAgIHBsb3QubWFyZ2luID0gdW5pdChjKDEwLDUsNSw1KSwibW0iKSwKICAgICAgICAgICAgICAgc3RyaXAuYmFja2dyb3VuZCA9IGVsZW1lbnRfcmVjdChjb2xvdXI9IiNmMGYwZjAiLGZpbGw9IiNmMGYwZjAiKSwKICAgICAgICAgICAgICAgc3RyaXAudGV4dCA9IGVsZW1lbnRfdGV4dChmYWNlPSJib2xkIiksCiAgICAgICAgICAgICAgIHN0cmlwLnRleHQueSA9IGVsZW1lbnRfdGV4dChhbmdsZSA9IDApCiAgICAgICAgICApKQogICAgICAKfQpgYGAKCmBgYHtyLCBmaWcuaGVpZ2h0PTYsIGZpZy53aWR0aD04fQpkZiA8LQogIGRmICU+JQogIG11dGF0ZShwb3NfbnVtPWFzLm51bWVyaWMoYXMuY2hhcmFjdGVyKHBvcykpKjQ1KQoKcHJlZGljdF9wb3AgJT4lCiAgZ2dwbG90KGFlcyhtb3JwaCwgcHJlZCoxMDAsIGNvbG9yPXNlc3Npb24pKSArCiAgZ2VvbV9zZWdtZW50KHg9LTEwLCB4ZW5kPTExMCwgeT01MCwgeWVuZD01MCwgc2l6ZT0wLjEsIGNvbG9yPSdsaWdodGdyYXknLCBhbHBoYT0wLjUsIGxpbmV0eXBlPSdkYXNoZWQnKSArCiAgZ2VvbV9zZWdtZW50KHg9NTAsIHhlbmQ9NTAsIHk9LTEwLCB5ZW5kPTExMCwgc2l6ZT0wLjEsIGNvbG9yPSdsaWdodGdyYXknLCBhbHBoYT0wLjUsIGxpbmV0eXBlPSdkYXNoZWQnKSArCiAgZ2VvbV9saW5lKCkgKwogICMgYWRkIGluZGl2aWR1YWwgZGF0YQogIHN0YXRfc3VtbWFyeShkYXRhPWRmLCAKICAgICAgICAgICAgICAgYWVzKG1vcnBoLCByZXNwb25zZV9iaW4pLCAKICAgICAgICAgICAgICAgZnVuLnk9ZnVuY3Rpb24oeCkgc3VtKHgpL2xlbmd0aCh4KSoxMDAsIGdlb209J3BvaW50JykgKwogIGZhY2V0X3dyYXAofiBwb3NfbnVtLCBuY29sPTQpICsKICBsYWJzKHg9J1BlcmNlbnRhZ2UgbW9ycGhpbmcnLCB5PSdQb3B1bGF0aW9uIHByZWRpY3Rpb25cbnBlcmNlbnQgcmVzcG9uc2VzIHRvIHNlY29uZCBpZGVudGl0eScsIGNvbG9yPSdTZXNzaW9uJykgKwogIHNjYWxlX2NvbG9yX2JyZXdlcihwYWxldHRlPSdTZXQxJykgKwogIHRoZW1lX1B1YmxpY2F0aW9uKCkgKyAKICBjb29yZF9lcXVhbCgpCiAgI3NjYWxlX3hfY29udGludW91cyhicmVha3M9YygwLCAxNywgMzMsIDUwLCA2NywgODMsIDEwMCkpCgpnZ3NhdmUoJy4uL2ltZy9wcmVkX3BvcF9nbW0ucG5nJywgd2lkdGg9OCwgaGVpZ2h0PTYpCmBgYAoKTm93IHdlIGNhbiBwcmVkaWN0IGZvciBlYWNoIGluZGl2aWR1YWwgc3ViamVjdApgYGB7cn0KZGZfcHJlZGljdCA8LQogIGV4cGFuZC5ncmlkKAogICAgbW9ycGhfcmVzYz1zZXEoLTAuNSwgMC41LCAwLjAxKSwgCiAgICBwb3M9dW5pcXVlKGRmJHBvcyksCiAgICBzdWJqZWN0PXVuaXF1ZShkZiRzdWJqZWN0KSkKCnByZWRpY3Rfc3Vial9zZXMxIDwtIHNhcHBseShsaXN0KG1vZGVsX3NlczEpLCBwcmVkaWN0LCBuZXdkYXRhPWRmX3ByZWRpY3QsIAogICAgICAgICAgICAgICAgICAgICAgICAgICB0eXBlPSdyZXNwb25zZScsIHNpbXBsaWZ5PUYsIFVTRS5OQU1FUz1UKQpwcmVkaWN0X3N1Ympfc2VzMiA8LSBzYXBwbHkobGlzdChtb2RlbF9zZXMyKSwgcHJlZGljdCwgbmV3ZGF0YT1kZl9wcmVkaWN0LCAKICAgICAgICAgICAgICAgICAgICAgICAgICAgdHlwZT0ncmVzcG9uc2UnLCBzaW1wbGlmeT1GLCBVU0UuTkFNRVM9VCkKCiMgYWRkIGRmX3ByZWRpY3QgdG8gZWFjaCBvZiB0aGVtCnByZWRpY3Rfc3Vial9zZXMxIDwtIGxhcHBseShwcmVkaWN0X3N1Ympfc2VzMSwgZnVuY3Rpb24oeCkgY2JpbmQoZGZfcHJlZGljdCwgcHJlZD14KSkKcHJlZGljdF9zdWJqX3NlczIgPC0gbGFwcGx5KHByZWRpY3Rfc3Vial9zZXMyLCBmdW5jdGlvbih4KSBjYmluZChkZl9wcmVkaWN0LCBwcmVkPXgpKQoKcHJlZGljdF9zdWJqX3NlczEgPC0gbGRwbHkocHJlZGljdF9zdWJqX3NlczEsIGRhdGEuZnJhbWUpCnByZWRpY3Rfc3Vial9zZXMyIDwtIGxkcGx5KHByZWRpY3Rfc3Vial9zZXMyLCBkYXRhLmZyYW1lKQpwcmVkaWN0X3N1Ympfc2VzMSRzZXNzaW9uIDwtICcxJwpwcmVkaWN0X3N1Ympfc2VzMiRzZXNzaW9uIDwtICcyJwoKcHJlZGljdF9zdWJqIDwtIHJiaW5kKHByZWRpY3Rfc3Vial9zZXMxLCBwcmVkaWN0X3N1Ympfc2VzMikKcHJlZGljdF9zdWJqIDwtIAogIHByZWRpY3Rfc3ViaiAlPiUKICBtdXRhdGUobW9ycGg9bW9ycGhfcmVzYyoxMDAgICsgNTAsIAogICAgICAgICBwb3NfbnVtPWFzLm51bWVyaWMoYXMuY2hhcmFjdGVyKHBvcykpKjQ1KQpgYGAKCk5vdyB3ZSBjYW4gc2F2ZSBlYWNoIGluZGl2aWR1YWwgcGxvdCB0byBkaXNrCgpgYGB7ciwgZmlnLmhlaWdodD02LCBmaWcud2lkdGg9OH0Kc3ViamVjdHMgPC0gdW5pcXVlKGRmJHN1YmplY3QpCmZvciAoc3ViaiBpbiBzdWJqZWN0cykgewogIG91dF9kaXIgPC0gZmlsZS5wYXRoKCcuLi9pbWcnLCAncHJlZF9nbW0nKQogIGZub3V0IDwtIGZpbGUucGF0aChvdXRfZGlyLCBwYXN0ZShzdWJqLCAnX3ByZWRfZ21tLnBuZycsIHNlcD0nJykpCiAgIyBzZXR1cCBkYXRhZnJhbWVzIGZvciBwbG90dGluZwogIHRoaXNfc3ViamVjdF9kZiA8LSBkZiAlPiUgCiAgICBmaWx0ZXIoc3ViamVjdCA9PSBzdWJqKSAlPiUKICAgICMgYWRkIHBvc2l0aW9uIGluIGFuZ2xlcwogICAgbXV0YXRlKHBvc19udW09YXMubnVtZXJpYyhhcy5jaGFyYWN0ZXIocG9zKSkqNDUpCiAgdGhpc19wcmVkaWN0X3N1YmogPC0gcHJlZGljdF9zdWJqICU+JSAKICAgIGZpbHRlcihzdWJqZWN0ID09IHN1YmopICU+JQogICAgIyBhZGQgcG9zaXRpb24gaW4gYW5nbGVzCiAgICBtdXRhdGUocG9zX251bT1hcy5udW1lcmljKGFzLmNoYXJhY3Rlcihwb3MpKSo0NSkKICAKICBwbG90X2N1cnZlIDwtCiAgICB0aGlzX3ByZWRpY3Rfc3ViaiAlPiUKICAgICMgYWRkIHByZWRpY3Rpb24KICAgIGdncGxvdChhZXMobW9ycGgsIHByZWQqMTAwLCBjb2xvcj1zZXNzaW9uKSkgKwogICAgZ2VvbV9zZWdtZW50KHg9LTEwLCB4ZW5kPTExMCwgeT01MCwgeWVuZD01MCwgc2l6ZT0wLjEsIGNvbG9yPSdsaWdodGdyYXknLCBhbHBoYT0wLjUsIGxpbmV0eXBlPSdkYXNoZWQnKSArCiAgICBnZW9tX3NlZ21lbnQoeD01MCwgeGVuZD01MCwgeT0tMTAsIHllbmQ9MTEwLCBzaXplPTAuMSwgY29sb3I9J2xpZ2h0Z3JheScsIGFscGhhPTAuNSwgbGluZXR5cGU9J2Rhc2hlZCcpICsKICAgIGdlb21fbGluZSgpICsKICAgICMgYWRkIGluZGl2aWR1YWwgZGF0YQogICAgc3RhdF9zdW1tYXJ5KGRhdGE9dGhpc19zdWJqZWN0X2RmLCAKICAgICAgICAgICAgICAgICBhZXMobW9ycGgsIHJlc3BvbnNlX2JpbiksIAogICAgICAgICAgICAgICAgIGZ1bi55PWZ1bmN0aW9uKHgpIHN1bSh4KS9sZW5ndGgoeCkqMTAwLCBnZW9tPSdwb2ludCcpICsKICAgIGZhY2V0X3dyYXAofnBvc19udW0sIG5jb2w9NCkgKwogICAgbGFicyh4PSdQZXJjZW50YWdlIG1vcnBoaW5nJywgeT0nUGVyY2VudCByZXNwb25zZXMgdG8gc2Vjb25kIGlkZW50aXR5JywgY29sb3I9J1Nlc3Npb24nKSArCiAgICBzY2FsZV9jb2xvcl9icmV3ZXIocGFsZXR0ZT0nU2V0MScpICsKICAgIGdndGl0bGUocGFzdGUoIlN1YmplY3QiLCBzdWJqKSkgKwogICAgdGhlbWVfUHVibGljYXRpb24oKSArCiAgICBjb29yZF9lcXVhbCgpICMrCiAgICAjc2NhbGVfeF9jb250aW51b3VzKGJyZWFrcz1jKDAsIDE3LCAzMywgNTAsIDY3LCA4MywgMTAwKSkKICAKICAgICMgc2F2ZQogICAgZGlyLmNyZWF0ZShvdXRfZGlyLCByZWN1cnNpdmU9VCkKICAgIGdnc2F2ZShmaWxlbmFtZT1mbm91dCwgcGxvdD1wbG90X2N1cnZlLCB3aWR0aD04LCBoZWlnaHQ9NikKfQpgYGAKCiMjIFN0YWJpbGl0eSBvZiBwb3B1bGF0aW9uIGxldmVsIGVzdGltYXRlcyBhY3Jvc3Mgc2Vzc2lvbnMKVGhlc2UgYXJlIHRoZSBwb3B1bGF0aW9uLWxldmVsIGVzdGltYXRlcwpgYGB7ciwgZWNobz1GQUxTRX0KIyBsZXQncyBtYWtlIGEgZGF0YWZyYW1lIGZvciBwbG90dGluZwptYWtlX3BzZXBfZGYgPC0gZnVuY3Rpb24obGlzdF9kZikgewogIGRmXyA8LSBkYXRhLmZyYW1lKHQoZGF0YS5mcmFtZShsaXN0X2RmKSkpCiAgIyBtYWtlIGl0IGxvbmcKICBkZl8gPC0gZGZfICU+JQogICAgZ2F0aGVyKHBvcywgcHNlKSAlPiUKICAgIG11dGF0ZShwb3NfbnVtPW1hcHZhbHVlcygKICAgICAgcG9zLCAKICAgICAgcGFzdGUoJ3BvcycsIHNlcSgwLCA3KSwgc2VwPScnKSwgCiAgICAgIHNlcSgwLCA3KSo0NSkpCiAgIyBhZGQgZmFjdG9yIGZvciBwb3NfbnVtCiAgZGZfJHBvc19udW0gPC0gZmFjdG9yKGRmXyRwb3NfbnVtLCBsZXZlbHM9c2VxKDAsIDcpKjQ1KQogIHJldHVybihkZl8pCn0KCmRmX3BzZXBfc2VzMSA8LSBtYWtlX3BzZXBfZGYocHNlcF9zZXMxKQpkZl9wc2VwX3NlczEkc2Vzc2lvbiA8LSAnMScKZGZfcHNlcF9zZXMyIDwtIG1ha2VfcHNlcF9kZihwc2VwX3NlczIpCmRmX3BzZXBfc2VzMiRzZXNzaW9uIDwtICcyJwoKcHNlcCA8LSByYmluZChkZl9wc2VwX3NlczEsIGRmX3BzZXBfc2VzMikKYGBgCgpMZXQncyBjb21wdXRlIGEgY29ycmVsYXRpb24gYWNyb3NzIHNlc3Npb25zCgpgYGB7ciwgZmlnLmhlaWdodD01LCBmaWcud2lkdGg9NX0KcHNlcF93aWRlIDwtIApwc2VwICU+JSAKICBzcHJlYWQoc2Vzc2lvbiwgcHNlKSAlPiUgCiAgbXV0YXRlKHNlc3Npb24xPS4kJzEnLCBzZXNzaW9uMj0uJCcyJykKCmdncGxvdChwc2VwX3dpZGUsIGFlcyhzZXNzaW9uMSoxMDAgKyA1MCwgc2Vzc2lvbjIqMTAwICsgNTAsIGdyb3VwPTEpKSArIAogIGdlb21fc21vb3RoKG1ldGhvZD0nbG0nLCBjb2xvcj0nZGFya2dyYXknLCBzZT1GKSArCiAgZ2VvbV9wb2ludCgpICsKICBsYWJzKHg9J0ZpcnN0IG1lYXN1cmVtZW50IChQU0UpJywgeT0nU2Vjb25kIG1lYXN1cmVtZW50IChQU0UpJywgc2hhcGU9J01vcnBoJykgKwogIHRoZW1lX1B1YmxpY2F0aW9uKCkgCiAgI2Nvb3JkX2VxdWFsKCkgKwogICNzY2FsZV94X2NvbnRpbnVvdXMoYnJlYWtzPXJvdW5kKHBzZXBfd2lkZSRzZXNzaW9uMSoxMDAgKyA1MCkpICsKICAjc2NhbGVfeV9jb250aW51b3VzKGJyZWFrcz1yb3VuZChwc2VwX3dpZGUkc2Vzc2lvbjIqMTAwICsgNTApKQoKZ2dzYXZlKCcuLi9pbWcvcHNlX3BvcF9zY2F0dGVyLnBuZycsIHdpZHRoPTUsIGhlaWdodD01KQpgYGAKQW5kIHRoZXNlIGFyZSB0aGUgY29ycmVsYXRpb24gdmFsdWVzCmBgYHtyfQpzZXQuc2VlZCgyMzQpCmJvb3RFUyhwc2VwX3dpZGVbYygnc2Vzc2lvbjEnLCAnc2Vzc2lvbjInKV0sIFI9MjAwMCkKYGBgCmBgYHtyfQpjb3IudGVzdChwc2VwX3dpZGUkc2Vzc2lvbjEsIHBzZXBfd2lkZSRzZXNzaW9uMikKYGBgCgojIyBTdGFiaWxpdHkgb2Ygc3ViamVjdC1sZXZlbCBlc3RpbWF0ZXMgYWNyb3NzIHNlc3Npb25zCkxldCdzIGFsc28gbG9vayBhdCB0aGUgJFxEZWx0YVx0ZXh0e1BTRX0kCmBgYHtyfQojIGdldCBkcHNlIGluIGxvbmcgZm9ybWF0IGZvciBwbG90dGluZwpkcHNlX3NlczFfbG9uZyA8LSAKICBkcHNlX3NlczEgJT4lCiAgbXV0YXRlKHN1YmplY3Q9cm93Lm5hbWVzKC4pKSAlPiUKICBnYXRoZXIocG9zLCBwc2UsIC1zdWJqZWN0KSAlPiUKICBtdXRhdGUoc2Vzc2lvbj0nMScpCmRwc2Vfc2VzMl9sb25nIDwtIAogIGRwc2Vfc2VzMiAlPiUKICBtdXRhdGUoc3ViamVjdD1yb3cubmFtZXMoLikpICU+JQogIGdhdGhlcihwb3MsIHBzZSwgLXN1YmplY3QpICU+JQogIG11dGF0ZShzZXNzaW9uPScyJykKCmRwc2UgPC0gCiAgcmJpbmQoZHBzZV9zZXMxX2xvbmcsIGRwc2Vfc2VzMl9sb25nKSAlPiUKICBtdXRhdGUocG9zX251bT1tYXB2YWx1ZXMocG9zLCAKICAgICAgICAgICAgICAgICAgICAgICAgICAgcGFzdGUoJ3BvcycsIDA6Nywgc2VwPScnKSwgCiAgICAgICAgICAgICAgICAgICAgICAgICAgIHNlcSgwLCA3KSo0NSkpCmRwc2UkcG9zX251bSA8LSBmYWN0b3IoZHBzZSRwb3NfbnVtLCBsZXZlbHM9c2VxKDAsIDcpKjQ1KQpgYGAKYGBge3IsIGZpZy5oZWlnaHQ9NSwgZmlnLndpZHRoPTV9CmRwc2Vfd2lkZSA8LQpkcHNlICU+JQogIHNwcmVhZChzZXNzaW9uLCBwc2UpICU+JQogIG11dGF0ZShzZXNzaW9uMT0uJCcxJywgc2Vzc2lvbjI9LiQnMicpCgpnZ3Bsb3QoZHBzZV93aWRlLCBhZXMoc2Vzc2lvbjEqMTAwLCBzZXNzaW9uMioxMDAsIGNvbG9yPXN1YmplY3QpKSArIAogIGdlb21fc21vb3RoKG1ldGhvZD0nbG0nLCBjb2xvcj0nZGFya2dyYXknLCBzZT1GKSArCiAgZ2VvbV9wb2ludCgpICsKICBsYWJzKHg9VGVYKCdGaXJzdCBtZWFzdXJlbWVudCAoJFxcRGVsdGEkUFNFKScpLCAKICAgICAgIHk9VGVYKCdTZWNvbmQgbWVhc3VyZW1lbnQgKCRcXERlbHRhJFBTRSknKSwgCiAgICAgICBjb2xvcj0nU3ViamVjdCcpICsKICB0aGVtZV9QdWJsaWNhdGlvbigpICsKICBjb29yZF9lcXVhbCgpICsKICAjZ3VpZGVzKGNvbG9yPUYpICsKICBzY2FsZV9jb2xvcl9icmV3ZXIocGFsZXR0ZT0nU2V0MScpICsKICB0aGVtZShsZWdlbmQucG9zaXRpb249YygwLjk1LCAwLjE1KSwKICAgICAgICBsZWdlbmQuZGlyZWN0aW9uPSd2ZXJ0aWNhbCcsCiAgICAgICAgbGVnZW5kLmtleS5zaXplPXVuaXQoLjgsICdwaWNhcycpLAogICAgICAgIGxlZ2VuZC50aXRsZT1lbGVtZW50X3RleHQoc2l6ZT0xMCkpCiAgI2Nvb3JkX2VxdWFsKHhsaW09YygtMjAsIDIwKSwgeWxpbT1jKC0yMCwgMjApKQogICNzY2FsZV94X2NvbnRpbnVvdXMoYnJlYWtzPXNlcSgtNjAsIDQwLCAyMCkpICsKICAjc2NhbGVfeV9jb250aW51b3VzKGJyZWFrcz1zZXEoLTYwLCA0MCwgMjApKQoKZ2dzYXZlKCcuLi9pbWcvcHNlX3N1Ympfc2NhdHRlci5wbmcnLCB3aWR0aD01LCBoZWlnaHQ9NSkKYGBgCkFuZCBjb3JyZWxhdGlvbiB2YWx1ZXMgYXMgd2VsbApgYGB7cn0Kc2V0LnNlZWQoMzI0KQpib290RVMoZHBzZV93aWRlW2MoJ3Nlc3Npb24xJywgJ3Nlc3Npb24yJyldLCBSPTEwMDAwKQpgYGAKYGBge3J9CmNvci50ZXN0KGRwc2Vfd2lkZSRzZXNzaW9uMSwgZHBzZV93aWRlJHNlc3Npb24yKQpgYGAKCgojIyBXaXRoaW4tIHZzLiBiZXR3ZWVuLXN1YmplY3RzIGNvcnJlbGF0aW9uIG9mIFBTRSBlc3RpbWF0ZXMKTGV0J3MgY29tcHV0ZSB0aGUgY29ycmVsYXRpb24gYmV0d2VlbiB0aGUgZmlyc3QgYW5kIHRoZSBzZWNvbmQgc2Vzc2lvbiB0byBjb21wYXJlCndpdGhpbiB2cy4gYmV0d2Vlbi1zdWJqZWN0IGNvcnJlbGF0aW9ucwpgYGB7cn0KdGhpc19jb3IgPC0gY29yKHQoZHBzZV9zZXMxKSwgdChkcHNlX3NlczIpKQojIG1ha2UgaXQgc3ltbWV0cmljCnRoaXNfY29yIDwtICh0aGlzX2NvciArIHQodGhpc19jb3IpKS8yLgpjb3Jfc2VzMTIgPC0gdGhpc19jb3IKCiMgbWFrZSBhIGRhdGFmcmFtZSBpbiBsb25nIGZvcm1hdAptYWtlX2Nvcl9sb25nIDwtIGZ1bmN0aW9uKGNvcikgewogIHdpdGhpbiA8LSBkaWFnKGNvcikKICBiZXR3ZWVuIDwtIGNvcltsb3dlci50cmkoY29yKV0KICAKICBkZl93aXRoaW5fYmV0d2VlbiA8LSBkYXRhLmZyYW1lKGNvcnI9Yyh3aXRoaW4sIGJldHdlZW4pLAogICAgICAgICAgICAgICAgICAgICAgICAgICAgICAgICAgdHlwZT1jKHJlcCgnd2l0aGluJywgbGVuZ3RoKHdpdGhpbikpLAogICAgICAgICAgICAgICAgICAgICAgICAgICAgICAgICAgICAgICAgIHJlcCgnYmV0d2VlbicsIGxlbmd0aChiZXR3ZWVuKSkpCiAgICAgICAgICAgICAgICAgICAgICAgICAgICAgICAgICApCiAgcmV0dXJuKGRmX3dpdGhpbl9iZXR3ZWVuKQp9Cgpjb3Jfc2VzMTJfbG9uZyA8LSBtYWtlX2Nvcl9sb25nKGNvcl9zZXMxMikKYGBgCgpXZSBjYW4gY2hlY2sgd2hldGhlciB0aGUgZXN0aW1hdGVzIGFyZSBjb25zaXN0ZW50IGFjcm9zcyBzZXNzaW9ucywgYW5kIGFsc28gCnN1YmplY3Qtc3BlY2lmaWMsIGJ5IGNvbXBhcmluZyB0aGUgd2l0aGluLXN1YmplY3QgY29ycmVsYXRpb25zIHdpdGggdGhlIGJldHdlZW4tc3ViamVjdApjb3JyZWxhdGlvbnMuIFdlIHdpbGwgY29tcHV0ZSB0aGUgYm9vdHN0cmFwcGVkIGRpZmZlcmVuY2UgJFx0ZXh0e1dpdGhpbn0gLSBcdGV4dHtCZXR3ZWVufSQuCmBgYHtyfQpyZXF1aXJlKGJvb3RFUykKcmVxdWlyZShicm9vbSkKYm9vdHN0cmFwX3dpdGhpbmJldHdlZW4gPC0gZnVuY3Rpb24oY29ycl9kZikgewogIGIgPC0gYm9vdEVTKGNvcnJfZGYsIAogICAgICAgICAgICAgIGRhdGEuY29sPSdjb3JyJywgZ3JvdXAuY29sPSd0eXBlJywgCiAgICAgICAgICAgICAgY29udHJhc3Q9Yyh3aXRoaW49MSwgYmV0d2Vlbj0tMSksIFI9MTAwMDApICAKICByZXR1cm4oYikKfQoKc2V0LnNlZWQoMTI0KQpib290X2NpcyA8LSBzYXBwbHkobGlzdChjb3Jfc2VzMTJfbG9uZyksCiAgICAgICAgICAgICAgICAgICBib290c3RyYXBfd2l0aGluYmV0d2VlbiwKICAgICAgICAgICAgICAgICAgIHNpbXBsaWZ5PUYsCiAgICAgICAgICAgICAgICAgICBVU0UuTkFNRVM9VCkKCmV4dHJhY3RfY2lzIDwtIGZ1bmN0aW9uKGJvb3Rlc19vdXQpIHsKICB0MCA8LSBib290ZXNfb3V0JHQwCiAgYm91bmRzIDwtIGJvb3Rlc19vdXQkYm91bmRzCiAgZGYgPC0gZGF0YS5mcmFtZSh0MD10MCwgbGNpPWJvdW5kc1sxXSwgcmNpPWJvdW5kc1syXSkKICByZXR1cm4oZGYpCn0KCmV4dHJhY3RfZGlzdHJpYnV0aW9uIDwtIGZ1bmN0aW9uKGJvb3Rlc19vdXQpIHsKICBkZiA8LSBkYXRhLmZyYW1lKHQ9Ym9vdGVzX291dCR0KQogIHJldHVybihkZikKfQpib290X2Npc19kZiA8LSBsZHBseShib290X2NpcywgZXh0cmFjdF9jaXMpCmJvb3RfZGlzdF9kZiA8LSBsZHBseShib290X2NpcywgZXh0cmFjdF9kaXN0cmlidXRpb24pCmBgYAoKQW5kIGZpbmFsbHkgcGxvdCB0aGVtCmBgYHtyLCBmaWcuaGVpZ2h0PTQsIGZpZy53aWR0aD02fQpnZ3Bsb3QoZGF0YT1ib290X2Rpc3RfZGYsIGFlcygxLCB0KSkgKwogIGdlb21fdmlvbGluKGFkanVzdD0yKSArCiAgZ2VvbV9lcnJvcmJhcihkYXRhPWJvb3RfY2lzX2RmLCBhZXMoeW1pbj1sY2ksIHltYXg9cmNpLCB5PXQwKSwgd2lkdGg9MC4wMSkgKyAKICBnZW9tX3BvaW50KGRhdGE9Ym9vdF9jaXNfZGYsIGFlcyh5PXQwKSkgKwogIGdlb21faGxpbmUoeWludGVyY2VwdD0wLCBsaW5ldHlwZT0nZGFzaGVkJykgKwogIGxhYnMoeD0nTW9ycGgnLCB5PSdXaXRoaW4gLSBCZXR3ZWVuIHN1YmplY3QgY29ycmVsYXRpb25zJykgKwogIHRoZW1lX1B1YmxpY2F0aW9uKCkgKwogIGNvb3JkX2ZsaXAoKSArIAogIHRoZW1lKGFzcGVjdC5yYXRpbz0zLzQpIApgYGAKQW5kIHRoZXNlIGFyZSB0aGUgdmFsdWVzCmBgYHtyfQpyb3VuZChib290X2Npc19kZiwgMikKYGBgCkNvbXB1dGUgYWxzbyBmb3Igd2l0aGluIGFuZCBiZXR3ZWVuCmBgYHtyfQpzZXQuc2VlZCg0MjU0KQpjb3Jfc2VzMTJfbG9uZyAlPiUgZmlsdGVyKHR5cGUgPT0gJ3dpdGhpbicpICU+JSBib290RVMoZGF0YS5jb2w9J2NvcnInLCBSPTEwMDAwKQpgYGAKYGBge3J9CnNldC5zZWVkKDQyNTQpCmNvcl9zZXMxMl9sb25nICU+JSBmaWx0ZXIodHlwZSA9PSAnYmV0d2VlbicpICU+JSBib290RVMoZGF0YS5jb2w9J2NvcnInLCBSPTEwMDAwKQpgYGAKCiMjIEFkZGl0aW9uYWwgcGxvdHMKCkxldCdzIG1ha2Ugc29tZSBwbG90cyB0byBzaG93IHRoZSBkaWZmZXJlbmNlIGluIHBzeWNob21ldHJpYyBjdXJ2ZXMgZm9yIGVhY2ggc3ViamVjdAoKYGBge3IsIGZpZy5oZWlnaHQ9NSwgZmlnLndpZHRoPTV9CnBzZV9zdWJqIDwtIGxpc3QoKQpwc2Vfc3VialtbMV1dIDwtICBkcHNlX3NlczEgKyBtYXRyaXgocmVwKHBzZXBfc2VzMSwgbnJvdyhkcHNlX3NlczEpKSwgCiAgICAgICAgICAgICAgICAgICAgICAgICAgICAgICAgICAgICBieXJvdz1ULCBucm93PW5yb3coZHBzZV9zZXMxKSkgCnBzZV9zdWJqW1syXV0gPC0gIGRwc2Vfc2VzMiArIG1hdHJpeChyZXAocHNlcF9zZXMyLCBucm93KGRwc2Vfc2VzMikpLCAKICAgICAgICAgICAgICAgICAgICAgICAgICAgICAgICAgICAgIGJ5cm93PVQsIG5yb3c9bnJvdyhkcHNlX3NlczIpKSAKCnBsb3RfZXhhbXBsZWZpdCA8LSBmdW5jdGlvbih3aGljaF9zdWJqLCBzZXM9MiwgZXh0cmVtZV9jdXJ2ZXM9TlVMTCkgewogIHBzZV9zdWJqX3NlcyAgPC0gcHNlX3N1YmpbW3Nlc11dCiAgCiAgcHNlX3N1Ympfc2VzIDwtCiAgcHNlX3N1Ympfc2VzICU+JQogICAgbXV0YXRlKHN1YmplY3Q9cm93Lm5hbWVzKC4pKSAlPiUKICAgIGdhdGhlcihwb3MsIHBzZSwgLXN1YmplY3QpICU+JQogICAgbXV0YXRlKHBzZT1wc2UqMTAwICsgNTAsCiAgICAgIHBvc19udW09bWFwdmFsdWVzKAogICAgICAgICAgICAgIHBvcywgCiAgICAgICAgICAgICAgcGFzdGUoJ3BvcycsIHNlcSgwLCA3KSwgc2VwPScnKSwgCiAgICAgICAgICAgICAgc2VxKDAsIDcpKjQ1KSwKICAgICAgcG9zPW1hcHZhbHVlcygKICAgICAgICAgICAgICBwb3MsIAogICAgICAgICAgICAgIHBhc3RlKCdwb3MnLCBzZXEoMCwgNyksIHNlcD0nJyksIAogICAgICAgICAgICAgIHNlcSgwLCA3KSkpICU+JQogICAgZmlsdGVyKHN1YmplY3QgPT0gd2hpY2hfc3ViaikKICAKICBwcmVkaWN0X3N1YmokcG9zX251bSA8LQogICAgZmFjdG9yKHByZWRpY3Rfc3ViaiRwb3NfbnVtLCBsZXZlbHM9c2VxKDAsIDcpKjQ1KQogIHBzZV9zdWJqX3NlcyRwb3NfbnVtIDwtCiAgICBmYWN0b3IocHNlX3N1Ympfc2VzJHBvc19udW0sIGxldmVscz1zZXEoMCwgNykqNDUpCiAgCiAgZGZfcGxvdCA8LSBkZiAlPiUKICAgIGZpbHRlcihzdWJqZWN0ID09IHdoaWNoX3N1YmosIHNlc3Npb24gPT0gYXMuY2hhcmFjdGVyKHNlcykpIAogIGRmX3Bsb3QkcG9zX251bSA8LSAKICAgIGZhY3RvcihkZl9wbG90JHBvc19udW0sIGxldmVscz1zZXEoMCwgNykqNDUpCiAgCiAgIyB0aGVzZSBhcmUgdGhlIGV4dHJlbWUgYW5ndWxhciBsb2NhdGlvbnMgZm9yIHRoZSB0d28gc3ViamVjdHMgd2UnbGwgcGxvdAogIGlmICghaXMubnVsbChleHRyZW1lX2N1cnZlcykpIHsKICAgIGRmX3Bsb3QgPC0KICAgIGRmX3Bsb3QgJT4lCiAgICAgIGZpbHRlcihwb3NfbnVtICVpbiUgZXh0cmVtZV9jdXJ2ZXMpCiAgfQogIAogICMgcGxvdAogIHBsb3QgPC0KICBwcmVkaWN0X3N1YmogJT4lCiAgICBmaWx0ZXIoc3ViamVjdCA9PSB3aGljaF9zdWJqLCBzZXNzaW9uID09IGFzLmNoYXJhY3RlcihzZXMpKSAlPiUKICAgIGdncGxvdChhZXMoKG1vcnBoX3Jlc2MqMTAwKSs1MCwgcHJlZCoxMDAsIGNvbG9yPXBvc19udW0pKSArCiAgICBnZW9tX3NlZ21lbnQoYWVzKHg9cHNlLCB4ZW5kPXBzZSwgeT0tMTAsIHllbmQ9NTApLCBhbHBoYT0wLjgsIGxpbmV0eXBlPSdkYXNoZWQnLCBkYXRhPXBzZV9zdWJqX3NlcykgKwogICAgZ2VvbV9saW5lKHNpemU9MC44KSArCiAgICB0aGVtZV9QdWJsaWNhdGlvbigpICsKICAgIGNvb3JkX2VxdWFsKHlsaW09YygtMC40LCAxMDEpKSArCiAgICBsYWJzKHg9J1BlcmNlbnRhZ2UgbW9ycGhpbmcnLCB5PSdQZXJjZW50YWdlIHJlc3BvbnNlc1xudG8gc2Vjb25kIGlkZW50aXR5JywgY29sb3I9J0FuZ3VsYXIgbG9jYXRpb24nKQogIAogIGlmICghaXMubnVsbChleHRyZW1lX2N1cnZlcykpIHsKICAgICAgcGxvdCA8LSBwbG90ICsKICAgICAgc3RhdF9zdW1tYXJ5KGZ1bi55PWZ1bmN0aW9uKHgpIHN1bSh4KS9sZW5ndGgoeCkqMTAwLCAKICAgICAgICAgICAgICAgICAgIGFlcyhtb3JwaCwgcmVzcG9uc2VfYmluKSwgZGF0YT1kZl9wbG90LCBnZW9tPSdwb2ludCcsCiAgICAgICAgICAgICAgICAgICBzaXplPTEuMiwgc2hvdy5sZWdlbmQ9RikgCiAgfQogIHJldHVybihwbG90KQogIH0KYGBgCmBgYHtyfQpleHRyZW1lX3ZhbHVlcyA8LSBsaXN0KAogIHMwMT1jKCc5MCcsICcyNzAnKSwKICBzMDI9YygnOTAnLCAnMzE1JyksCiAgczAzPWMoJzkwJywgJzIyNScpLAogIHMwND1jKCcwJywgJzI3MCcpCikKYGBgCgoKYGBge3IsIGZpZy5oZWlnaHQ9NSwgZmlnLndpZHRoPTV9CmZvciAoc2VzIGluIDE6MikgewogIGZvciAocyBpbiBzdWJqZWN0cykgewogICAgcGxvdCA8LSBwbG90X2V4YW1wbGVmaXQocywgc2VzLCBleHRyZW1lX3ZhbHVlc1tbc11dKQogICAgZ2dzYXZlKHBhc3RlKCcuLi9pbWcvZXhhbXBsZV9maXRfJywgcywgJ19zZXMnLCBzZXMsICcucG5nJywgc2VwPScnKSwKICAgICAgICAgICB3aWR0aD01LCBoZWlnaHQ9NSkKICB9Cn0KYGBgCg==
