# Supplementary material for: Idiosyncratic, Retinotopic Bias in Face Identification Modulated by Familiarity
Source: eNeuro. 2018 Oct 4;5(5):ENEURO.0054-18.2018. doi: 10.1523/ENEURO.0054-18.2018 (PMC6171739; doi:10.1523/ENEURO.0054-18.2018)
Supplement: Extended Data — The archive contains data from both experiments, as well as the analysis scripts. Download Extended Data 1, ZIP file. [file sup_enu-eN-NWR-0054-18-s02.zip › famretino2-3.0.0/exp1/code/analysis_exp1.pdf]

Experiment 1

Contents

Procedure and equations **1**

Model fitting **2**

Predict psychometric curves . . . 3
Stability of population level estimates across sessions . . . 7
Stability of subject-level estimates across sessions . . . 8
Within- vs. between-subjects correlation of PSE estimates . . . 10
Additional plots . . . 12

Start by loading functions and data

```
require(latex2exp)
require(bootES)
require(plyr)
require(tidyverse)
require(assertthat)
df <- read_csv('../data/data.csv')
df$pos <- as.factor(df$pos)
df$session <- as.factor(df$session)
```

Procedure and equations

We are going to fit a linear mixed effect model to the data. We will model the data as follows

$$y^k = \text{logit}\left(g(x)\right) g(x) = \beta_0 x + \sum_{i=1}^{8} \left(\beta_i + z_i^k\right) I_i$$

Where y^k is the response for subject k, x is the (scaled) percentage of morphing, $\beta_i, i = 1...4$ are the fixed-effects for each angular location (0 to 315 in 45 deg steps), and z_i^k are the random-effects (random slopes for location) for each subject, and I_i is an indicator variable, indicating the angular location for each trial.

In this way for each subject we can find the PSE as the point where $y^k = 0.5$, that is the point $\hat{x}$

$$\text{logit}(g(\hat{x})) = 0.5 \iff g(\hat{x}) = 0 \iff \beta_0 \hat{x} + \sum_{i=1}^{4} \left(\beta_i + z_i^k\right) I_i = 0 \iff \hat{x} = -\frac{\sum_{i=1}^{4} \left(\beta_i + z_i^k\right) I_i}{\beta_0}$$

Thus for every angular location i we have that

$$\hat{x}_i = -\frac{\beta_i}{\beta_0} - \frac{z_i^k}{\beta_0} = \text{PSE}_i^p + \Delta\text{PSE}_i^s$$

with PSE_i^p being the population-level PSE at location i, and ΔPSE_i^s being the change at location i for subject s.

We will fit one such model for each of the morph types, and one for each session.

Model fitting

```r
require(lme4)

# define some functions
extract_morph_session <- function(df, ses) {
  # Extract trials from one particular  session
  df_ <-
    df %>%
    filter(session == ses) %>%
    mutate(morph_resc=(morph - 50)/100)
  return(df_)
}
run_model_session <- function(df) {
  # Run the following logit mixed-effect model for one session
  # response_bin ~ morph_resc + pos - 1 + (pos - 1 | subject)
  m <- glmer(response_bin ~ morph_resc + pos - 1 + (pos - 1 | subject),
           data=df,
           family=binomial(link='logit'),
           control=glmerControl(optimizer='bobyqa', optCtrl=list(maxfun=100000)))
  return(m)
}
```

Run the model separately for every morph

```r
df_ses1 <- extract_morph_session(df, '1')
```

```
## Warning: package 'bindrcpp' was built under R version 3.2.5
```

```r
df_ses2 <- extract_morph_session(df, '2')
assert_that(sum(nrow(df_ses1), nrow(df_ses2)) == nrow(df))
```

```
## [1] TRUE
```

```r
# now compute models
model_ses1 <- run_model_session(df_ses1)
model_ses2 <- run_model_session(df_ses2)
```

The following functions are used to extract both the population PSE^p and the subject ΔPSE^s. Remember that $\text{PSE}^s = \text{PSE}^p + \Delta\text{PSE}^s$.

```r
population_pse <- function(model) {
  # Computes population-level PSE (see equations above)
  # Note: they are on the scale of morph_resc
  morph_beta <- fixef(model)[1]
  pos_betas <- fixef(model)[-1]
  return(-pos_betas/morph_beta)
}

subjects_pse <- function(model) {
  # Computes subject-level PSE (see equations above)
  morph_pop <- fixef(model)[1]
  position_pop <- fixef(model)[-1]
  ranef_model <- ranef(model)$subject
  # this is the denominator
  morph_subj <- ranef_model[, 1] + morph_pop
```

```
  # these are the numerators
  position_subj <- ranef_model[, -1]
  # extend position_beta to get the same shape as ranef_pos
  position_pop <- matrix(rep(position_pop, nrow(position_subj)),
                        byrow=T, nrow=nrow(position_subj))
  position_subj <- position_subj + position_pop
  # extend morph_subj to get the same shape as position_subj
  morph_subj <- matrix(rep(morph_subj, ncol(position_subj)), ncol=ncol(position_subj))
  return(-position_subj/morph_subj)
}

delta_pse <- function(model) {
  # Computes subject-level PSE (see equations above)
  # Note: they are on the scale of morph_resc
  # IF 'morph_resc' is entered as a random effect, add that for each individual
  # morph
  morph_beta <- fixef(model)[1]
  ranef_model <- ranef(model)$subject
  if ('morph_resc' %in% names(ranef_model)) {
    pse_pop <- population_pse(model)
    pse_subj <- subjects_pse(model)
    pse_pop <- matrix(rep(pse_pop, nrow(pse_subj)), nrow=nrow(pse_subj), byrow=T)
    return(pse_subj - pse_pop)
  } else {
    return(-ranef_model/morph_beta)
  }
}
```

Let's look at the population estimates for the PSEs across the two sessions, as well as the subject-level estimates.

```
psep_ses1 <- population_pse(model_ses1)
psep_ses2 <- population_pse(model_ses2)
dpse_ses1 <- delta_pse(model_ses1)
dpse_ses2 <- delta_pse(model_ses2)
```

Predict psychometric curves

Let's plot the population estimates first

```
df_predict <-
  expand.grid(morph_resc=seq(-0.5, 0.5, 0.01), pos=unique(df$pos))

predict_pop_ses1 <- sapply(list(model_ses1), predict, newdata=df_predict,
                           re.form=NA, type='response', simplify=F, USE.NAMES=T)
predict_pop_ses2 <- sapply(list(model_ses2), predict, newdata=df_predict,
                           re.form=NA, type='response', simplify=F, USE.NAMES=T)

# add df_predict to each of them
predict_pop_ses1 <- lapply(predict_pop_ses1, function(x) cbind(df_predict, pred=x))
predict_pop_ses2 <- lapply(predict_pop_ses2, function(x) cbind(df_predict, pred=x))

predict_pop_ses1 <- ldply(predict_pop_ses1, data.frame)
predict_pop_ses2 <- ldply(predict_pop_ses2, data.frame)
```

```r
predict_pop_ses1$session <- '1'
predict_pop_ses2$session <- '2'

predict_pop <- rbind(predict_pop_ses1, predict_pop_ses2)
predict_pop <-
  predict_pop %>%
  mutate(morph=morph_resc*100  + 50,
         pos_num=as.numeric(as.character(pos))*45)
```

```r
# modified from https://rpubs.com/Koundy/71792
theme_Publication <- function(base_size=12) {
      library(ggthemes)
      (theme_foundation(base_size=base_size)
       + theme(plot.title = element_text(face = "bold",
                                         size = rel(1.2), hjust = 0.5),
               text = element_text(),
               panel.background = element_rect(colour = NA),
               plot.background = element_rect(colour = NA),
               panel.border = element_rect(colour = NA),
               axis.title = element_text(size = rel(1)),
               axis.title.y = element_text(angle=90,vjust =2),
               axis.title.x = element_text(vjust = -0.2),
               axis.text = element_text(),
               axis.line = element_line(colour="black"),
               axis.ticks = element_line(),
               panel.grid.major = element_blank(),#element_line(colour="#f0f0f0"),
               panel.grid.minor = element_blank(),
               legend.key = element_rect(colour = NA),
               legend.position = "bottom",
               legend.direction = "horizontal",
               #legend.key.size= unit(0.2, "cm"),
               legend.spacing = unit(0, "cm"),
               legend.title = element_text(),
               plot.margin = unit(c(10,5,5,5),"mm"),
               strip.background = element_rect(colour="#f0f0f0",fill="#f0f0f0"),
               strip.text = element_text(face="bold"),
               strip.text.y = element_text(angle = 0)
          ))

}
```

```r
df <-
  df %>%
  mutate(pos_num=as.numeric(as.character(pos))*45)

predict_pop %>%
  ggplot(aes(morph, pred*100, color=session)) +
  geom_segment(x=-10, xend=110, y=50, yend=50, size=0.1, color='lightgray', alpha=0.5, linetype='dashed
  geom_segment(x=50, xend=50, y=-10, yend=110, size=0.1, color='lightgray', alpha=0.5, linetype='dashed
  geom_line() +
  # add individual data
  stat_summary(data=df,
               aes(morph, response_bin),
               fun.y=function(x) sum(x)/length(x)*100, geom='point') +
```

```
  facet_wrap(~ pos_num, ncol=4) +
  labs(x='Percentage morphing', y='Population prediction\npercent responses to second identity', color=
  scale_color_brewer(palette='Set1') +
  theme_Publication() +
  coord_equal()
```

```
## Warning: package 'ggthemes' was built under R version 3.2.5
```

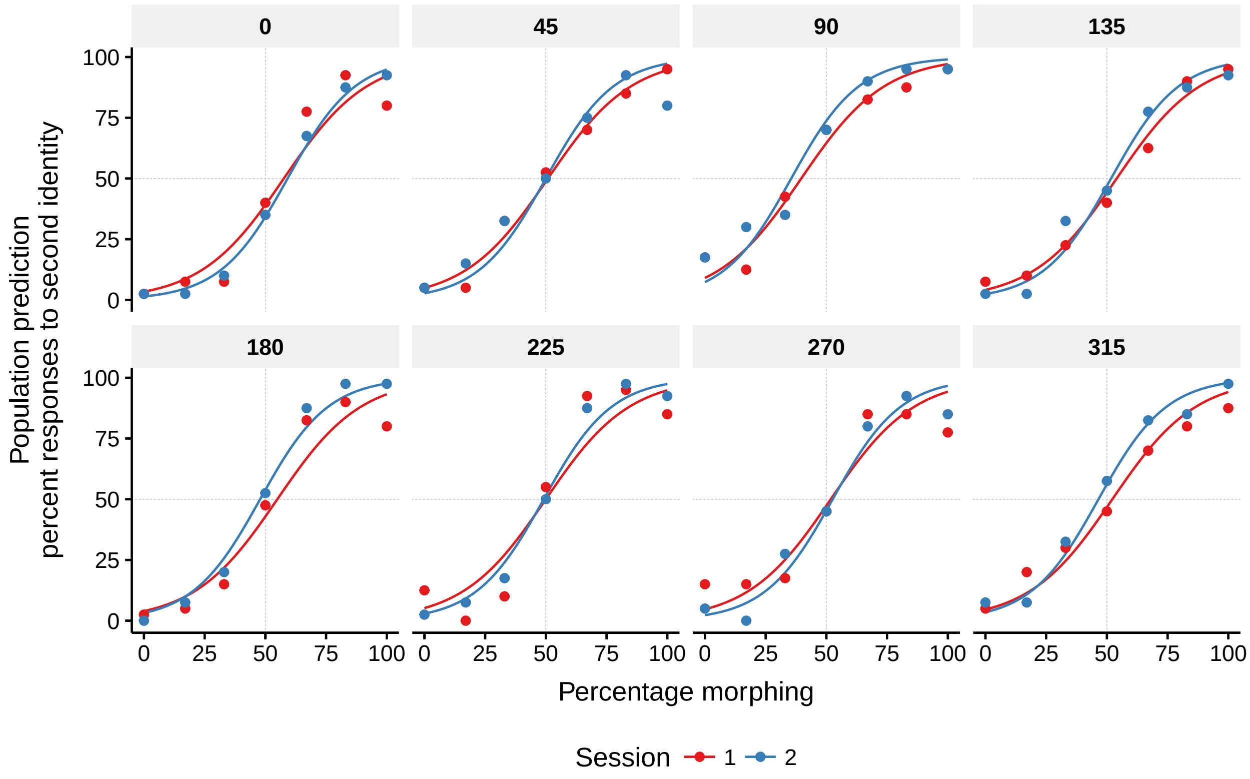


```
  #scale_x_continuous(breaks=c(0, 17, 33, 50, 67, 83, 100))

ggsave('../img/pred_pop_gmm.png', width=8, height=6)
```

Now we can predict for each individual subject

```
df_predict <-
  expand.grid(
    morph_resc=seq(-0.5, 0.5, 0.01),
    pos=unique(df$pos),
    subject=unique(df$subject))

predict_subj_ses1 <- sapply(list(model_ses1), predict, newdata=df_predict,
                            type='response', simplify=F, USE.NAMES=T)
predict_subj_ses2 <- sapply(list(model_ses2), predict, newdata=df_predict,
                            type='response', simplify=F, USE.NAMES=T)

# add df_predict to each of them
predict_subj_ses1 <- lapply(predict_subj_ses1, function(x) cbind(df_predict, pred=x))
predict_subj_ses2 <- lapply(predict_subj_ses2, function(x) cbind(df_predict, pred=x))

predict_subj_ses1 <- ldply(predict_subj_ses1, data.frame)
```

```
predict_subj_ses2 <- ldply(predict_subj_ses2, data.frame)
predict_subj_ses1$session <- '1'
predict_subj_ses2$session <- '2'

predict_subj <- rbind(predict_subj_ses1, predict_subj_ses2)
predict_subj <-
  predict_subj %>%
  mutate(morph=morph_resc*100  + 50,
         pos_num=as.numeric(as.character(pos))*45)
```

Now we can save each individual plot to disk

```
subjects <- unique(df$subject)
for (subj in subjects) {
  out_dir <- file.path('../img', 'pred_gmm')
  fnout <- file.path(out_dir, paste(subj, '_pred_gmm.png', sep=''))
  # setup dataframes for plotting
  this_subject_df <- df %>%
    filter(subject == subj) %>%
    # add position in angles
    mutate(pos_num=as.numeric(as.character(pos))*45)
  this_predict_subj <- predict_subj %>%
    filter(subject == subj) %>%
    # add position in angles
    mutate(pos_num=as.numeric(as.character(pos))*45)

  plot_curve <-
    this_predict_subj %>%
    # add prediction
    ggplot(aes(morph, pred*100, color=session)) +
    geom_segment(x=-10, xend=110, y=50, yend=50, size=0.1, color='lightgray', alpha=0.5, linetype='dash
    geom_segment(x=50, xend=50, y=-10, yend=110, size=0.1, color='lightgray', alpha=0.5, linetype='dash
    geom_line() +
    # add individual data
    stat_summary(data=this_subject_df,
                 aes(morph, response_bin),
                 fun.y=function(x) sum(x)/length(x)*100, geom='point') +
    facet_wrap(~pos_num, ncol=4) +
    labs(x='Percentage morphing', y='Percent responses to second identity', color='Session') +
    scale_color_brewer(palette='Set1') +
    ggtitle(paste("Subject", subj)) +
    theme_Publication() +
    coord_equal() #+
    #scale_x_continuous(breaks=c(0, 17, 33, 50, 67, 83, 100))

    # save
    dir.create(out_dir, recursive=T)
    ggsave(filename=fnout, plot=plot_curve, width=8, height=6)
}
```

```
## Warning in dir.create(out_dir, recursive = T): '../img/pred_gmm' already
## exists

## Warning in dir.create(out_dir, recursive = T): '../img/pred_gmm' already
```

```
## exists

## Warning in dir.create(out_dir, recursive = T): '../img/pred_gmm' already
## exists

## Warning in dir.create(out_dir, recursive = T): '../img/pred_gmm' already
## exists
```

Stability of population level estimates across sessions

These are the population-level estimates

Let's compute a correlation across sessions

```
psep_wide <-
psep %>%
  spread(session, pse) %>%
  mutate(session1=.$'1', session2=.$'2')

ggplot(psep_wide, aes(session1*100 + 50, session2*100 + 50, group=1)) +
  geom_smooth(method='lm', color='darkgray', se=F) +
  geom_point() +
  labs(x='First measurement (PSE)', y='Second measurement (PSE)', shape='Morph') +
  theme_Publication()
```

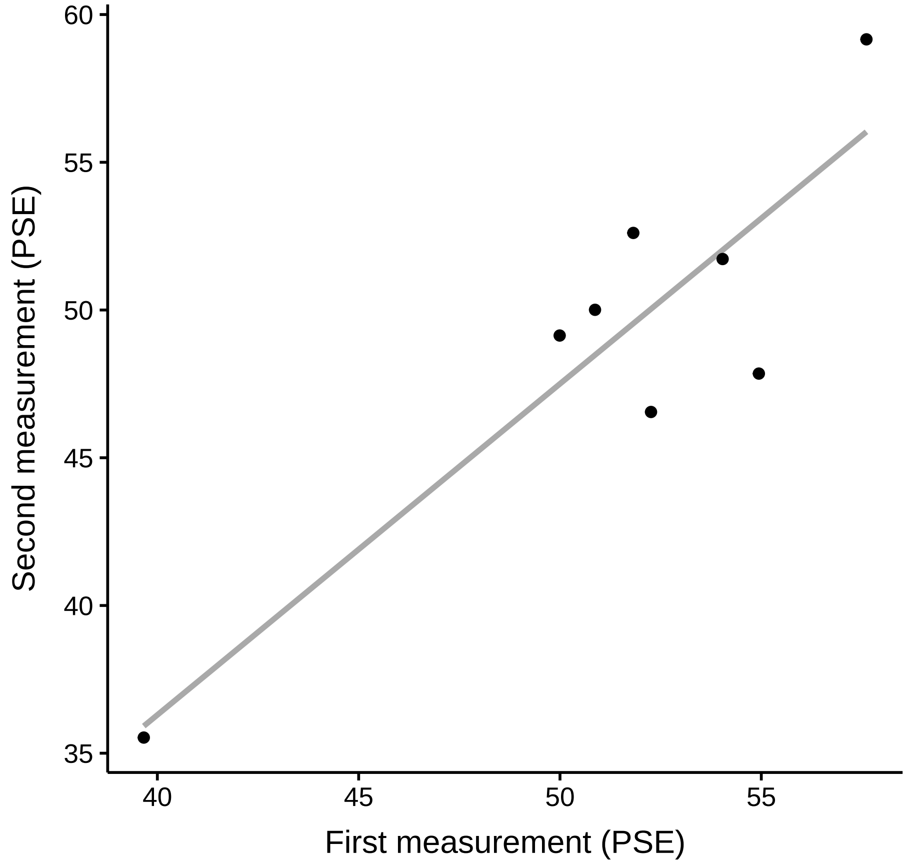


```
  #coord_equal() +
  #scale_x_continuous(breaks=round(psep_wide$session1*100 + 50)) +
```

```r
  #scale_y_continuous(breaks=round(psep_wide$session2*100 + 50))

ggsave('../img/pse_pop_scatter.png', width=5, height=5)
```

And these are the correlation values

```r
set.seed(234)
bootES(psep_wide[c('session1', 'session2')], R=2000)
```

```
##
## 95.00% bca Confidence Interval, 2000 replicates
## Stat        CI (Low)    CI (High)   bias        SE
## 0.893       -0.233      0.996       -0.115      0.317
```

```r
cor.test(psep_wide$session1, psep_wide$session2)
```

```
##
##  Pearson's product-moment correlation
##
## data:  psep_wide$session1 and psep_wide$session2
## t = 4.8572, df = 6, p-value = 0.002831
## alternative hypothesis: true correlation is not equal to 0
## 95 percent confidence interval:
##  0.5075767 0.9805832
## sample estimates:
##       cor
## 0.8928861
```

Stability of subject-level estimates across sessions

Let's also look at the ΔPSE

```r
# get dpse in long format for plotting
dpse_ses1_long <-
  dpse_ses1 %>%
  mutate(subject=row.names(.)) %>%
  gather(pos, pse, -subject) %>%
  mutate(session='1')
dpse_ses2_long <-
  dpse_ses2 %>%
  mutate(subject=row.names(.)) %>%
  gather(pos, pse, -subject) %>%
  mutate(session='2')

dpse <-
  rbind(dpse_ses1_long, dpse_ses2_long) %>%
  mutate(pos_num=mapvalues(pos,
                         paste('pos', 0:7, sep=''),
                         seq(0, 7)*45))
dpse$pos_num <- factor(dpse$pos_num, levels=seq(0, 7)*45)
```

```r
dpse_wide <-
dpse %>%
  spread(session, pse) %>%
  mutate(session1=.$'1', session2=.$'2')
```

```r
ggplot(dpse_wide, aes(session1*100, session2*100, color=subject)) +
  geom_smooth(method='lm', color='darkgray', se=F) +
  geom_point() +
  labs(x=TeX('First measurement ($\\Delta$PSE)'),
       y=TeX('Second measurement ($\\Delta$PSE)'),
       color='Subject') +
  theme_Publication() +
  coord_equal() +
  #guides(color=F) +
  scale_color_brewer(palette='Set1') +
  theme(legend.position=c(0.95, 0.15),
        legend.direction='vertical',
        legend.key.size=unit(.8, 'picas'),
        legend.title=element_text(size=10))
```

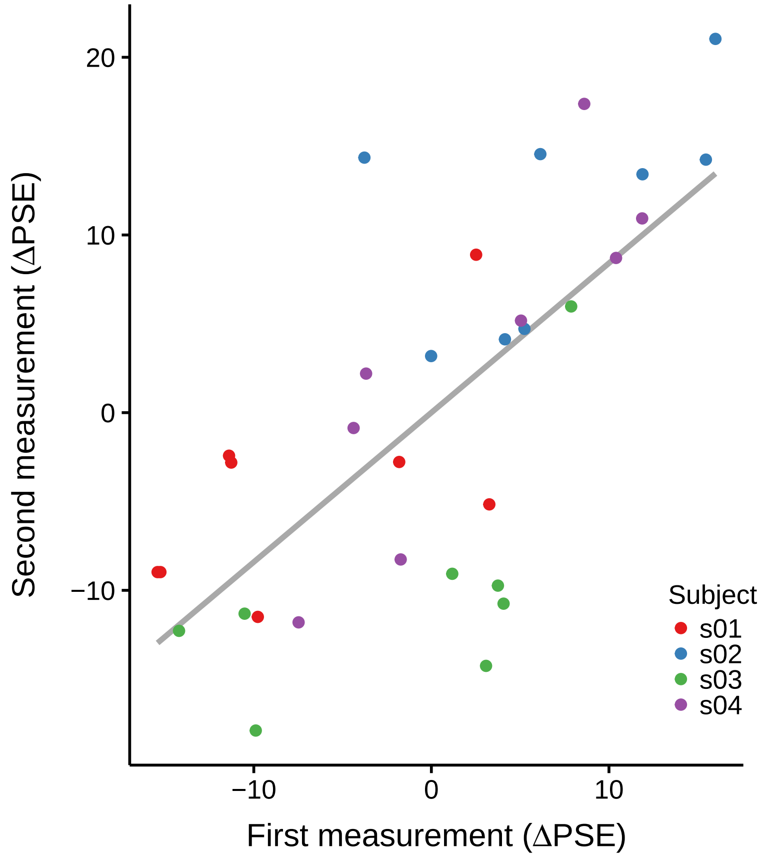


```r
  #coord_equal(xlim=c(-20, 20), ylim=c(-20, 20))
  #scale_x_continuous(breaks=seq(-60, 40, 20)) +
  #scale_y_continuous(breaks=seq(-60, 40, 20))

ggsave('../img/pse_subj_scatter.png', width=5, height=5)
```

And correlation values as well

```r
set.seed(324)
bootES(dpse_wide[c('session1', 'session2')], R=10000)
```

```
##
```

```
## 95.00% bca Confidence Interval, 10000 replicates
## Stat        CI (Low)    CI (High)   bias        SE
## 0.707       0.464       0.842       -0.004      0.092
```

```
cor.test(dpse_wide$session1, dpse_wide$session2)
```

```
##
##  Pearson's product-moment correlation
##
## data:  dpse_wide$session1 and dpse_wide$session2
## t = 5.4743, df = 30, p-value = 6.106e-06
## alternative hypothesis: true correlation is not equal to 0
## 95 percent confidence interval:
##  0.4754108 0.8468616
## sample estimates:
##       cor
## 0.7069171
```

Within- vs. between-subjects correlation of PSE estimates

Let's compute the correlation between the first and the second session to compare within vs. between-subject correlations

```
this_cor <- cor(t(dpse_ses1), t(dpse_ses2))
# make it symmetric
this_cor <- (this_cor + t(this_cor))/2.
cor_ses12 <- this_cor

# make a dataframe in long format
make_cor_long <- function(cor) {
  within <- diag(cor)
  between <- cor[lower.tri(cor)]

  df_within_between <- data.frame(corr=c(within, between),
                                  type=c(rep('within', length(within)),
                                         rep('between', length(between)))
                                  )
  return(df_within_between)
}

cor_ses12_long <- make_cor_long(cor_ses12)
```

We can check whether the estimates are consistent across sessions, and also subject-specific, by comparing the within-subject correlations with the between-subject correlations. We will compute the bootstrapped difference Within – Between.

```
require(bootES)
require(broom)
```

```
## Loading required package: broom
```

```
## Warning: package 'broom' was built under R version 3.2.5
```

```
bootstrap_withinbetween <- function(corr_df) {
  b <- bootES(corr_df,
              data.col='corr', group.col='type',
```

```r
               contrast=c(within=1, between=-1), R=10000)
  return(b)
}

set.seed(124)
boot_cis <- sapply(list(cor_ses12_long),
                   bootstrap_withinbetween,
                   simplify=F,
                   USE.NAMES=T)

extract_cis <- function(bootes_out) {
  t0 <- bootes_out$t0
  bounds <- bootes_out$bounds
  df <- data.frame(t0=t0, lci=bounds[1], rci=bounds[2])
  return(df)
}

extract_distribution <- function(bootes_out) {
  df <- data.frame(t=bootes_out$t)
  return(df)
}
boot_cis_df <- ldply(boot_cis, extract_cis)
boot_dist_df <- ldply(boot_cis, extract_distribution)
```

And finally plot them

```r
ggplot(data=boot_dist_df, aes(1, t)) +
  geom_violin(adjust=2) +
  geom_errorbar(data=boot_cis_df, aes(ymin=lci, ymax=rci, y=t0), width=0.01) +
  geom_point(data=boot_cis_df, aes(y=t0)) +
  geom_hline(yintercept=0, linetype='dashed') +
  labs(x='Morph', y='Within - Between subject correlations') +
  theme_Publication() +
  coord_flip() +
  theme(aspect.ratio=3/4)
```

```
## Warning: Ignoring unknown aesthetics: y
```

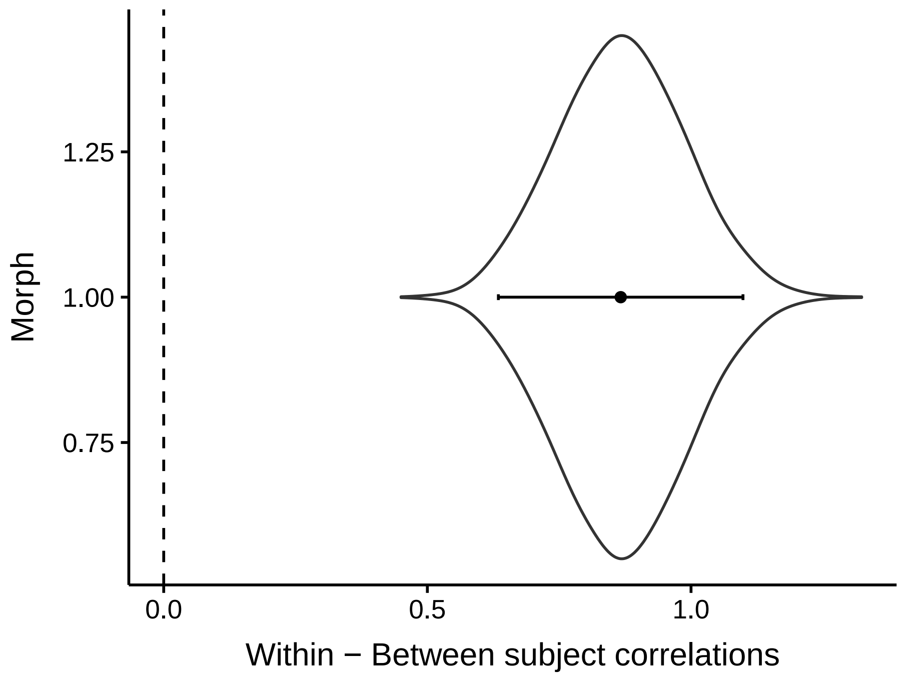

And these are the values

```
round(boot_cis_df, 2)
```

```
##     t0  lci rci
## 1 0.87 0.63 1.1
```

Compute also for within and between

```
set.seed(4254)
cor_ses12_long %>% filter(type == 'within') %>% bootES(data.col='corr', R=10000)
```

```
##
## 95.00% bca Confidence Interval, 10000 replicates
## Stat        CI (Low)    CI (High)   bias        SE
## 0.651       0.565       0.795       0.000       0.060
```

```
set.seed(4254)
cor_ses12_long %>% filter(type == 'between') %>% bootES(data.col='corr', R=10000)
```

```
##
## 95.00% bca Confidence Interval, 10000 replicates
## Stat        CI (Low)    CI (High)   bias        SE
## -0.216      -0.406      -0.011      -0.002      0.101
```

Additional plots

Let's make some plots to show the difference in psychometric curves for each subject

```
plot_examplefit_ses2 <- function(which_subj, extreme_curves=NULL) {
  pse_subj_ses2 <-
    dpse_ses2 + matrix(rep(psep_ses2, nrow(dpse_ses2)), byrow=T, nrow=nrow(dpse_ses2))

  pse_subj_ses2 <-
  pse_subj_ses2 %>%
    mutate(subject=row.names(.)) %>%
```

```
    gather(pos, pse, -subject) %>%
    mutate(pse=pse*100 + 50,
      pos_num=mapvalues(
              pos,
              paste('pos', seq(0, 7), sep=''),
              seq(0, 7)*45),
      pos=mapvalues(
              pos,
              paste('pos', seq(0, 7), sep=''),
              seq(0, 7))) %>%
    filter(subject == which_subj)

  predict_subj$pos_num <-
    factor(predict_subj$pos_num, levels=seq(0, 7)*45)
  pse_subj_ses2$pos_num <-
    factor(pse_subj_ses2$pos_num, levels=seq(0, 7)*45)

  df_plot <- df %>%
    filter(subject == which_subj, session == '2')
  df_plot$pos_num <-
    factor(df_plot$pos_num, levels=seq(0, 7)*45)

  # these are the extreme angular locations for the two subjects we'll plot
  if (!is.null(extreme_curves)) {
    df_plot <-
    df_plot %>%
      filter(pos_num %in% extreme_curves)
  }

  # plot
  plot <-
  predict_subj %>%
    filter(subject == which_subj, session == '2') %>%
    ggplot(aes((morph_resc*100)+50, pred*100, color=pos_num)) +
    geom_segment(aes(x=pse, xend=pse, y=-10, yend=50), alpha=0.8, linetype='dashed', data=pse_subj_ses2
    geom_line(size=0.8) +
    theme_Publication() +
    coord_equal(ylim=c(-0.4, 101)) +
    labs(x='Percentage morphing', y='Percentage responses\nto second identity', color='Angular location

  if (!is.null(extreme_curves)) {
      plot <- plot +
      stat_summary(fun.y=function(x) sum(x)/length(x)*100,
                   aes(morph, response_bin), data=df_plot, geom='point',
                   size=1.2, show.legend=F)
  }
  return(plot)
  }
```

```
extreme_values <- list(
  s01=c('90', '270'),
  s02=c('90', '315'),
  s03=c('90', '225'),
  s04=c('0', '270')
```

```
)
```

```
for (s in subjects) {
  plot <- plot_examplefit_ses2(s, extreme_values[[s]])
  ggsave(paste('../img/example_fit_', s, '.png', sep=''),
         width=5, height=5)
}
```
