# Supplementary material for: Idiosyncratic, Retinotopic Bias in Face Identification Modulated by Familiarity
Source: eNeuro. 2018 Oct 4;5(5):ENEURO.0054-18.2018. doi: 10.1523/ENEURO.0054-18.2018 (PMC6171739; doi:10.1523/ENEURO.0054-18.2018)
Supplement: Extended Data — The archive contains data from both experiments, as well as the analysis scripts. Download Extended Data 1, ZIP file. [file sup_enu-eN-NWR-0054-18-s02.zip › famretino2-3.0.0/exp2/code/analysis_exp2.nb.html]

Experiment 2


Code 

- Show All Code
- Hide All Code
- Download Rmd

# Experiment 2

- Procedure and equations
- Model fitting
  - Predict psychometric curves
  - Stability of population level estimates across sessions
  - Stability of subject-level estimates across sessions
  - Within- vs. between-subjects correlation
  - Correlation with familiarity ratings
  - Is it personal familiarity or is it contact?
- PSE for individual identities
  - Use the model to predict the biases

Start by loading functions and data


```
require(latex2exp)
require(bootES)
# load plyr *BEFORE* dplyr (i.e., tidyverse)
require(plyr)
require(tidyverse)
require(assertthat)
require(broom)
df <- read_csv('../data/data.csv')
# create output img directory if not existant
dir.create('../img', showWarning=F)
```


Preprocess the data, that is do the following

- remove trials where participants responded with a third option; that is, if they were presented with morph \(ab\) and they responded with \(c\), that trial will be removed
- binarize the responses to run the model


```
df <- df %>%
  filter(third_option != 1)
# now we need to add a binarized response to compute the psychometric curves
# but this changes depending on morph_type, so we need to make a function that
# checks the conditions
binarize_response <- function(morph_type, response_identity) {
  if (morph_type == 'ab') {
    return(ifelse(response_identity == 'a', 0, 1))
  } else if (morph_type == 'bc') {
    return(ifelse(response_identity == 'b', 0, 1))
  } else if (morph_type == 'ac') {
    return(ifelse(response_identity == 'a', 0, 1))
  }
}
df <-
df %>%
  rowwise() %>%
  mutate(response_bin=binarize_response(morph_type, response_identity))
df$pos <- as.factor(df$pos)
df$session <- as.factor(df$session)
# make sure that binarize_response worked, i.e. we should get 1s and 0s for each
# morph_type
check_ <-
df %>% 
  group_by(morph_type) %>% 
  summarise(check=length(unique(response_bin)))
assert_that(length(check_$check) == 3)
```


```
[1] TRUE
```


```
assert_that(all(check_$check == c(2, 2, 2)))
```


```
[1] TRUE
```


# Procedure and equations

We are going to fit a linear mixed effect model to the data. We will model the data as follows

\[
y^k = \text{logit}\left(g(x)\right)\\
g(x) = \beta\_0 x + \sum\_{i=1}^8\left(\beta\_i + z\_i^k\right)I\_i
\] Where \(y^k\) is the response for subject \(k\), \(x\) is the (scaled) percentage of morphing, \(\beta\_i, i=1...4\) are the fixed-effects for each angular location (0 to 315 in 45 deg steps), and \(z\_i^k\) are the random-effects (random slopes for location) for each subject, and \(I\_i\) is an indicator variable, indicating the angular location for each trial.

In this way for each subject we can find the PSE as the point where \(y^k = 0.5\), that is the point \(\hat{x}\)

\[
\text{logit}(g(\hat{x})) = 0.5 \Longleftrightarrow
g(\hat{x}) = 0 \Longleftrightarrow \\
\beta\_0 \hat{x} + \sum\_{i=1}^4\left(\beta\_i + z\_i^k\right)I\_i = 0 \Longleftrightarrow\\
\hat{x} = -\frac{\sum\_{i=1}^4\left(\beta\_i + z\_i^k\right)I\_i}{\beta\_0}
\]

Thus for every angular location \(i\) we have that

\[
\hat{x}\_i = -\frac{\beta\_i}{\beta\_0} - \frac{z\_i^k}{\beta\_0} = \text{PSE}^p\_i + \Delta\text{PSE}\_i^s\\
\]

with \(\text{PSE}\_i^p\) being the population-level PSE at location \(i\), and \(\Delta\text{PSE}\_i^s\) being the change at location \(i\) for subject \(s\).

We will fit one such model for each of the morph types, and one for each session.

# Model fitting


```
require(lme4)
# define some functions
extract_morph_session <- function(df, mt, ses) {
  # Extract trials from one particular morph_type and session
  df_ <-
    df %>%
    filter(morph_type == mt, session == ses) %>%
    mutate(morph_resc=(morph - 50)/100)
  return(df_)
}
run_model_session <- function(df) {
  # Run the following logit mixed-effect model for one session
  # response_bin ~ morph_resc + pos - 1 + (pos - 1 | subject)
  m <- glmer(response_bin ~ morph_resc + pos - 1 + (pos - 1 | subject), 
           data=df, 
           family=binomial(link='logit'), 
           control=glmerControl(optimizer='bobyqa', optCtrl=list(maxfun=20000)))
  return(m)
}
```


Run the model separately for every morph


```
morphs <- unique(df$morph_type)
df_ses1 <- sapply(morphs, function(x) extract_morph_session(df, x, '1'), 
                  simplify=F, USE.NAMES=T) 
df_ses2 <- sapply(morphs, function(x) extract_morph_session(df, x, '2'), 
                  simplify=F, USE.NAMES=T) 
# check that we have all the trials
assert_that(sum(sapply(df_ses1, nrow)) + sum(sapply(df_ses2, nrow)) == nrow(df))
```


```
[1] TRUE
```


```
# now compute models
model_ses1 <- sapply(df_ses1, run_model_session, simplify=F, USE.NAMES=T)
model_ses2 <- sapply(df_ses2, run_model_session, simplify=F, USE.NAMES=T)
```


The following functions are used to extract both the population \(\text{PSE}^p\) and the subject \(\Delta\text{PSE}^s\). Remember that \(\text{PSE}^s = \text{PSE}^p + \Delta\text{PSE}^s\).


```
population_pse <- function(model) {
  # Computes population-level PSE (see equations above)
  # Note: they are on the scale of morph_resc
  morph_beta <- fixef(model)[1]
  pos_betas <- fixef(model)[-1]
  return(-pos_betas/morph_beta)
}
subjects_pse <- function(model) {
  # Computes subject-level PSE (see equations above)
  morph_pop <- fixef(model)[1]
  position_pop <- fixef(model)[-1]
  ranef_model <- ranef(model)$subject
  # this is the denominator
  morph_subj <- ranef_model[, 1] + morph_pop
  # these are the numerators
  position_subj <- ranef_model[, -1]  
  # extend position_beta to get the same shape as ranef_pos
  position_pop <- matrix(rep(position_beta, nrow(ranef_pos)), 
                          byrow=T, nrow=nrow(ranef_pos))
  position_subj <- position_subj + position_pop
  # extend morph_subj to get the same shape as position_subj
  morph_subj <- matrix(rep(morph_subj, ncol(position_subj)), ncol=ncol(position_subj)) 
  return(-position_subj/morph_subj)
}
delta_pse <- function(model) {
  # Computes subject-level PSE (see equations above)
  # Note: they are on the scale of morph_resc
  # IF 'morph_resc' is entered as a random effect, add that for each individual
  # morph
  morph_beta <- fixef(model)[1]
  ranef_model <- ranef(model)$subject
  if ('morph_resc' %in% names(ranef_model)) {
    pse_pop <- population_pse(model)
    pse_subj <- subjects_pse(model)
    pse_pop <- matrix(rep(pse_pop, nrow(pse_subj)), nrow=nrow(pse_subj), byrow=T)
    return(pse_subj - pse_pop) 
  } else {
    return(-ranef_model/morph_beta)
  }
}
```


Let’s look at the population estimates for the PSEs across the two sessions, as well as the subject-level estimates.


```
psep_ses1 <- sapply(model_ses1, population_pse, simplify=F, USE.NAMES=T)
psep_ses2 <- sapply(model_ses2, population_pse, simplify=F, USE.NAMES=T)
dpse_ses1 <- sapply(model_ses1, delta_pse, simplify=F, USE.NAMES=T)
dpse_ses2 <- sapply(model_ses2, delta_pse, simplify=F, USE.NAMES=T)
```


## Predict psychometric curves

Let’s plot the population estimates first


```
df_predict <-
  expand.grid(morph_resc=seq(-0.5, 0.5, 0.01), pos=c('1', '3', '5', '7'))
predict_pop_ses1 <- sapply(model_ses1, predict, newdata=df_predict, 
                           re.form=NA, type='response', simplify=F, USE.NAMES=T)
predict_pop_ses2 <- sapply(model_ses2, predict, newdata=df_predict, 
                           re.form=NA, type='response', simplify=F, USE.NAMES=T)
# add df_predict to each of them
predict_pop_ses1 <- lapply(predict_pop_ses1, function(x) cbind(df_predict, pred=x))
predict_pop_ses2 <- lapply(predict_pop_ses2, function(x) cbind(df_predict, pred=x))
predict_pop_ses1 <- ldply(predict_pop_ses1, data.frame)
predict_pop_ses2 <- ldply(predict_pop_ses2, data.frame)
predict_pop_ses1$session <- '1'
predict_pop_ses2$session <- '2'
predict_pop <- rbind(predict_pop_ses1, predict_pop_ses2)
predict_pop <- 
  predict_pop %>%
  mutate(morph=morph_resc*100  + 50, 
         pos_num=as.numeric(as.character(pos))*45,
         morph_type=.id)
```


```
# modified from https://rpubs.com/Koundy/71792
theme_Publication <- function(base_size=12) {
      library(ggthemes)
      (theme_foundation(base_size=base_size)
       + theme(plot.title = element_text(face = "bold",
                                         size = rel(1.2), hjust = 0.5),
               text = element_text(),
               panel.background = element_rect(colour = NA),
               plot.background = element_rect(colour = NA),
               panel.border = element_rect(colour = NA),
               axis.title = element_text(size = rel(1)),
               axis.title.y = element_text(angle=90,vjust =2),
               axis.title.x = element_text(vjust = -0.2),
               axis.text = element_text(), 
               axis.line = element_line(colour="black"),
               axis.ticks = element_line(),
               panel.grid.major = element_blank(), #element_line(colour="#f0f0f0"),
               panel.grid.minor = element_blank(),
               legend.key = element_rect(colour = NA),
               legend.position = "bottom",
               legend.direction = "horizontal",
               #legend.key.size= unit(0.2, "cm"),
               legend.spacing = unit(0, "cm"),
               legend.title = element_text(),
               plot.margin = unit(c(10,5,5,5),"mm"),
               strip.background = element_rect(colour="#f0f0f0",fill="#f0f0f0"),
               strip.text = element_text(face="bold"),
               strip.text.y = element_text(angle = 0)
          ))
      
}
```


```
df <-
  df %>%
  mutate(pos_num=as.numeric(as.character(pos))*45)
predict_pop %>%
  ggplot(aes(morph, pred*100, color=session)) +
  geom_line() +
  geom_hline(yintercept=50, size=0.5, color='darkgray', alpha=1, linetype='dashed') +
  geom_vline(xintercept=50, size=0.5, color='darkgray', alpha=1, linetype='dashed') +
  # add individual data
  stat_summary(data=df, 
               aes(morph, response_bin), 
               fun.y=function(x) sum(x)/length(x)*100, geom='point') +
  facet_grid(morph_type ~ pos_num) +
  labs(x='Percentage morphing', y='Population prediction\npercent responses to second identity', color='Session') +
  scale_color_brewer(palette='Set1') +
  theme_Publication() + 
  coord_equal()
```


```
package ‘ggthemes’ was built under R version 3.2.5
```


```
ggsave('../img/pred_pop_gmm.png', width=8, height=6)
```


Now we can predict for each individual subject


```
df_predict <-
  expand.grid(
    morph_resc=seq(-0.5, 0.5, 0.01), 
    pos=c('1', '3', '5', '7'),
    subject=unique(df$subject))
predict_subj_ses1 <- sapply(model_ses1, predict, newdata=df_predict, 
                           type='response', simplify=F, USE.NAMES=T)
predict_subj_ses2 <- sapply(model_ses2, predict, newdata=df_predict, 
                           type='response', simplify=F, USE.NAMES=T)
# add df_predict to each of them
predict_subj_ses1 <- lapply(predict_subj_ses1, function(x) cbind(df_predict, pred=x))
predict_subj_ses2 <- lapply(predict_subj_ses2, function(x) cbind(df_predict, pred=x))
predict_subj_ses1 <- ldply(predict_subj_ses1, data.frame)
predict_subj_ses2 <- ldply(predict_subj_ses2, data.frame)
predict_subj_ses1$session <- '1'
predict_subj_ses2$session <- '2'
predict_subj <- rbind(predict_subj_ses1, predict_subj_ses2)
predict_subj <- 
  predict_subj %>%
  mutate(morph=morph_resc*100  + 50, 
         pos_num=as.numeric(as.character(pos))*45,
         morph_type=.id)
```


Now we can save each individual plot to disk


```
subjects <- unique(df$subject)
for (subj in subjects) {
  out_dir <- file.path('../img', 'pred_gmm')
  fnout <- file.path(out_dir, paste(subj, '_pred_gmm.png', sep=''))
  # setup dataframes for plotting
  this_subject_df <- df %>% 
    filter(subject == subj) %>%
    # add position in angles
    mutate(pos_num=as.numeric(as.character(pos))*45)
  this_predict_subj <- predict_subj %>% 
    filter(subject == subj) %>%
    # add position in angles
    mutate(pos_num=as.numeric(as.character(pos))*45)
  
  plot_curve <-
    this_predict_subj %>%
    # add prediction
    ggplot(aes(morph, pred*100, color=session)) +
    geom_hline(yintercept=50, size=0.5, color='darkgray', alpha=1, linetype='dashed') +
    geom_vline(xintercept=50, size=0.5, color='darkgray', alpha=1, linetype='dashed') +
    geom_line() +
    # add individual data
    stat_summary(data=this_subject_df, 
                 aes(morph, response_bin), 
                 fun.y=function(x) sum(x)/length(x)*100, geom='point') +
    facet_grid(morph_type ~ pos_num) +
    labs(x='Percentage morphing', y='Percent responses to second identity', color='Session') +
    scale_color_brewer(palette='Set1') +
    ggtitle(paste("Subject", subj)) +
    theme_Publication() +
    coord_equal() 
  
    # save
    dir.create(out_dir, recursive=T)
    ggsave(filename=fnout, plot=plot_curve, width=8, height=6)
}
```


```
'../img/pred_gmm' already exists
```


These are the population-level estimates


```
psep %>%
  ggplot(aes(pos_num, pse*100 + 50, color=session, group=session)) +
  geom_hline(yintercept=50, linetype='dashed', alpha=0.8) +
  geom_point() +
  geom_line() +
  labs(x='Angular location', y='PSE (population)', color='Session') +
  #ggtitle('Population-level PSE') +
  scale_color_brewer(palette='Set1') +
  facet_grid(~morph_type) +
  scale_y_continuous(breaks=c(30, 50, 70)) +
  theme_Publication()
```


```
NA
```

## Stability of population level estimates across sessions

Let’s compute a correlation across sessions


```
psep_wide <- 
psep %>% 
  spread(session, pse) %>% 
  mutate(session1=.$'1', session2=.$'2')
ggplot(psep_wide, aes(session1*100 + 50, session2*100 + 50, shape=morph_type, group=1)) + 
  geom_smooth(method='lm', color='darkgray', se=F) +
  geom_point() +
  labs(x='First measurement (PSE)', y='Second measurement (PSE)', shape='Morph') +
  theme_Publication() + 
  coord_equal() +
  #guides(shape=F)
  theme(legend.position=c(0.95, 0.15),
        legend.direction='vertical',
        legend.key.size=unit(.8, 'picas'),
        legend.title=element_text(size=10))
```


```
  #coord_equal(xlim=c(20, 95), ylim=c(20, 95)) +
  #scale_x_continuous(breaks=seq(20, 90, 10)) +
  #scale_y_continuous(breaks=seq(20, 90, 10))
ggsave('../img/pse_pop_scatter.png', width=5, height=5)
```


And these are the correlations


```
set.seed(3432)
bootES(psep_wide[c('session1', 'session2')], R=10000)
```


```
95.00% bca Confidence Interval, 10000 replicates
Stat        CI (Low)    CI (High)   bias        SE          
0.979       0.929       0.992       0.000       0.011
```


```
cor.test(psep_wide$session1, psep_wide$session2)
```


```
    Pearson's product-moment correlation

data:  psep_wide$session1 and psep_wide$session2
t = 15.219, df = 10, p-value = 3.042e-08
alternative hypothesis: true correlation is not equal to 0
95 percent confidence interval:
 0.9248693 0.9942948
sample estimates:
      cor 
0.9790871
```


They are very consistent across sessions.

## Stability of subject-level estimates across sessions


```
# add morph type
for (morph in morphs) {
  dpse_ses1[[morph]]$subject <- row.names(dpse_ses1[[morph]])
  dpse_ses2[[morph]]$subject <- row.names(dpse_ses2[[morph]])
}
# get dpse in long format for plotting
dpse_ses1_long <-
  ldply(
    lapply(dpse_ses1, function(x) gather(x, pos, pse, -subject)),
    data.frame) %>%
  mutate(morph_type=.id, session='1')
dpse_ses2_long <-
  ldply(
    lapply(dpse_ses2, function(x) gather(x, pos, pse, -subject)),
    data.frame) %>%
  mutate(morph_type=.id, session='2')
dpse <- 
  rbind(dpse_ses1_long, dpse_ses2_long) %>%
  mutate(pos_num=mapvalues(pos, 
                           paste('pos', c(1, 3, 5, 7), sep=''), 
                           c(1, 3, 5, 7)*45))
dpse$pos_num <- factor(dpse$pos_num, levels=c(1, 3, 5, 7)*45)
```


Average the estimates across sessions and plot them


```
dpse %>% 
  group_by(subject, morph_type, pos_num) %>% 
  summarise(pse=mean(pse)) %>%
  ggplot(aes(pos_num, pse*100, color=subject, group=subject)) +
  geom_point(alpha=0.8) +
  geom_line(alpha=0.8) +
  facet_wrap(~morph_type, nrow=1) +
  theme_Publication() +
  labs(x='Angular location', y=TeX('$\\Delta$PSE'), color='Subject') +
  scale_y_continuous(limits=c(-60, 50)) +
  theme(#legend.position=c(0.95, 0.15),
        legend.direction='vertical',
        legend.position='right',
        legend.key.size=unit(.8, 'picas'),
        legend.title=element_text(size=10))
```


```
ggsave('../img/dpse_subjects.png', width=8, height=3)
```


Let’s also plot an example fit for subject s10


```
# get the data to plot individual points
df_s10_plot_ab <-
  df %>%
  filter(subject == 's10', session == '1') %>%
  filter(morph_type == 'ab', pos_num %in% c('225', '315'))
df_s10_plot_ac <-
  df %>%
  filter(subject == 's10', session == '1') %>%
  filter(morph_type == 'ac', pos_num %in% c('135', '315'))
df_s10_plot_bc <-
  df %>%
  filter(subject == 's10', session == '1') %>%
  filter(morph_type == 'bc', pos_num %in% c('135', '315'))
df_s10_plot <-
  rbind(df_s10_plot_ab, df_s10_plot_ac, df_s10_plot_bc)
# now get the pse values to plot
psep1_plot <-
psep %>% 
  filter(session == '1') %>%
  arrange(pos, morph_type)
dpse1_plot <-
dpse %>% 
  filter(subject == 's10', session == '1') %>% 
  arrange(pos, morph_type) %>%
  mutate(dpse=pse) %>%
  select(morph_type, pos, pos_num, dpse)
pse_s10_plot <- merge(psep1_plot, dpse1_plot)
pse_s10_plot <-
pse_s10_plot %>%
  mutate(tpse=(pse+dpse)*100+50)
```


```
predict_subj %>%
  filter(subject == 's10', session == '1') %>%
  ggplot(aes(morph_resc*100 + 50, pred*100, color=as.factor(pos_num))) +
  geom_segment(aes(x=tpse, xend=tpse, y=-10, yend=50), alpha=0.8, linetype='dashed', data=pse_s10_plot) +
  geom_line() +
  facet_wrap(~morph_type, ncol=1) +
  coord_equal(ylim=c(-0.4, 101)) +
  theme_Publication() +
  theme(#legend.position=c(0.95, 0.15),
        legend.direction='vertical',
        legend.position='right',
        legend.key.size=unit(.8, 'picas'),
        legend.title=element_text(size=10)) +
  labs(x='Percentage morphing', y='Percentage responses to\nsecond identity', color='Angular\nlocation') +
  stat_summary(aes(morph, response_bin), fun.y=function(x) sum(x)/length(x)*100, geom='point', 
               data=df_s10_plot, size=0.9, show.legend=F)
```


```
ggsave('../img/examplefit_s10.png', width=8, height=6)
```


Correlation of subject-level estimates across sessions


```
dpse_wide <-
dpse %>%
  spread(session, pse) %>%
  mutate(session1=.$'1', session2=.$'2')
ggplot(dpse_wide, aes(session1*100, session2*100, color=subject)) + 
  geom_smooth(method='lm', color='darkgray', se=F) +
  geom_point() +
  labs(x=TeX('First measurement ($\\Delta$PSE)'), 
       y=TeX('Second measurement ($\\Delta$PSE)'), 
       color='Subject') +
  theme_Publication() +
  coord_equal() +
  theme(legend.position=c(0.95, 0.25),
        legend.direction='vertical',
        legend.key.size=unit(.8, 'picas'),
        legend.title=element_text(size=10))
```


```
  #coord_equal(xlim=c(-60, 45), ylim=c(-60, 45)) +
  #scale_x_continuous(breaks=seq(-60, 40, 20)) +
  #scale_y_continuous(breaks=seq(-60, 40, 20))
ggsave('../img/pse_subj_scatter.png', width=5, height=5)
```


And these are the correlations.


```
set.seed(23448)
bootES(dpse_wide[c('session1', 'session2')], R=10000)
```


```
95.00% bca Confidence Interval, 10000 replicates
Stat        CI (Low)    CI (High)   bias        SE          
0.639       0.499       0.750       -0.003      0.064
```


```
cor.test(dpse_wide$session1, dpse_wide$session2)
```


```
    Pearson's product-moment correlation

data:  dpse_wide$session1 and dpse_wide$session2
t = 9.0205, df = 118, p-value = 3.997e-15
alternative hypothesis: true correlation is not equal to 0
95 percent confidence interval:
 0.5190456 0.7340393
sample estimates:
      cor 
0.6388518
```

## Within- vs. between-subjects correlation

Let’s compute the correlation between the first and the second session


```
cor_ses12 <- list()
for (morph in morphs) {
  this_cor <- cor(t(dpse_ses1[[morph]][, 1:4]), t(dpse_ses2[[morph]][, 1:4]))
  # make it symmetric
  this_cor <- (this_cor + t(this_cor))/2.
  cor_ses12[[morph]] = this_cor
}
# make a dataframe in long format
make_cor_long <- function(cor) {
  within <- diag(cor)
  between <- cor[lower.tri(cor)]
  
  df_within_between <- data.frame(corr=c(within, between),
                                  type=c(rep('within', length(within)),
                                         rep('between', length(between)))
                                  )
  return(df_within_between)
}
cor_ses12_long <- sapply(cor_ses12, make_cor_long, simplify=F, USE.NAMES=T)
# add everything together for plotting
cor_ses12_long_plot <- ldply(cor_ses12_long, data.frame)
```


We can check whether the estimates are consistent across sessions, and also subject-specific, by comparing the within-subject correlations with the between-subject correlations. We will compute the bootstrapped difference \(\text{Within} - \text{Between}\).


```
require(bootES)
require(broom)
```


```
Loading required package: broom
package ‘broom’ was built under R version 3.2.5
```


```
bootstrap_withinbetween <- function(corr_df) {
  b <- bootES(corr_df, 
              data.col='corr', group.col='type', 
              contrast=c(within=1, between=-1), R=10000)  
  return(b)
}
set.seed(124)
boot_cis <- sapply(cor_ses12_long,
                   bootstrap_withinbetween,
                   simplify=F,
                   USE.NAMES=T)
extract_cis <- function(bootes_out) {
  t0 <- bootes_out$t0
  bounds <- bootes_out$bounds
  df <- data.frame(t0=t0, lci=bounds[1], rci=bounds[2])
  return(df)
}
extract_distribution <- function(bootes_out) {
  df <- data.frame(t=bootes_out$t)
  return(df)
}
boot_cis_df <- ldply(boot_cis, extract_cis)
boot_dist_df <- ldply(boot_cis, extract_distribution)
```


And finally plot them


```
ggplot(data=boot_dist_df, aes(.id, t)) +
  geom_violin(adjust=2) +
  geom_errorbar(data=boot_cis_df, aes(ymin=lci, ymax=rci, y=t0), width=0.01) + 
  geom_point(data=boot_cis_df, aes(y=t0)) +
  geom_hline(yintercept=0, linetype='dashed') +
  labs(x='Morph', y='Within - Between subject correlations') +
  theme_Publication() +
  coord_flip() + 
  theme(aspect.ratio=3/4)
```


```
Ignoring unknown aesthetics: y
```


And these are the values


```
boot_cis_df %>% arrange(.id)
```


Let’s also do it separately for within and between


```
bootstrap <- function(corr_df) {
  b <- bootES(corr_df, 
              data.col='corr', R=10000)  
  return(b)
}
cor_ses12_long_within <- sapply(cor_ses12_long, 
                                function(x) filter(x, type=='within'),
                                simplify=F,
                                USE.NAMES=T)
cor_ses12_long_between <- sapply(cor_ses12_long, 
                                function(x) filter(x, type=='between'),
                                simplify=F,
                                USE.NAMES=T)
set.seed(3243)
bs_within <- ldply(
  lapply(cor_ses12_long_within, bootstrap),
  extract_cis)
set.seed(23423)
bs_between <- ldply(
  lapply(cor_ses12_long_between, bootstrap),
  extract_cis)
```


```
bs_within %>% arrange(.id)
```


```
bs_between %>% arrange(.id)
```

## Correlation with familiarity ratings


```
df_quest <- read_csv('../data/questionnaire.csv',
                     col_types='ccddddddddddddd')
```


We are going to create a composite score by simply averaging the questions related to closeness.


```
# add composite score
df_quest <-
df_quest %>%
  # everybody got the name right
  select(-name) %>%
  rowwise() %>%
  # Let's make a composite score by averaging ios, wescale, sci1, sci2
  mutate(compscore=mean(c(ios, wescale, sci1, sci2)))
```


Let’s start simply by looking at the average values for each id


```
df_quest_long <-
df_quest %>%
  gather(scale, value, -subject, -stim)
```


```
df_quest_long %>%
  ggplot(aes(scale, value, color=stim)) +
  stat_summary(fun.data=mean_cl_boot, geom='errorbar', width=0.1, position=position_dodge(w=0.3)) +
  stat_summary(fun.y=mean, geom='point', position=position_dodge(w=0.3)) +
  theme_Publication() +
  theme(axis.text.x=element_text(angle=45, hjust=1))
ggsave('../img/scores.png', width=8, height=6)
```


```
# compute a score of deviance from 0 for each subject and morph type
# basically the variance
dpse_deviance <-
dpse %>%
  group_by(subject, morph_type, pos_num) %>% 
  summarise(pse=mean(pse)) %>%
  group_by(subject, morph_type) %>%
  summarise(psescore=sum(pse^2))
# now for each subject compute the average score for the morphs
df_quest_avg <-
df_quest %>%
  select(subject, stim, compscore) %>%
  spread(stim, compscore) %>%
  rowwise() %>%
  mutate(score_ab=mean(c(a, b)), 
         score_bc=mean(c(b, c)), 
         score_ac=mean(c(a, c))) %>%
  select(-a, -b, -c) %>%
  gather(morph_type, score, -subject)
df_quest_avg$morph_type <-
  mapvalues(df_quest_avg$morph_type,
            c('score_ab', 'score_ac', 'score_bc'),
            c('ab', 'ac', 'bc'))
df_quest_deviance <-
df_quest_avg %>%
  arrange(subject, morph_type) %>%
  merge(dpse_deviance)
```


```
ggplot(aes(score, psescore, color=morph_type), data=df_quest_deviance) +
  geom_smooth(method='lm', aes(group=1), se=F, color='darkgray') + 
  geom_point() +
  theme_Publication() +
  coord_fixed(ratio=10) +
  labs(x='Familiarity score', y=TeX('$\\Delta$PSE variance'), color='Morph type') + 
  theme(legend.position=c(0.95, 0.6),
        legend.direction='vertical',
        legend.key.size=unit(.8, 'picas'),
        legend.title=element_text(size=10)) +
  scale_color_brewer(palette='Set2')
```


```
ggsave('../img/psequest_scatter.png', width=5, height=5)
```


```
cor.test(df_quest_deviance$score, df_quest_deviance$psescore)
```


```
    Pearson's product-moment correlation

data:  df_quest_deviance$score and df_quest_deviance$psescore
t = -3.5895, df = 28, p-value = 0.001248
alternative hypothesis: true correlation is not equal to 0
95 percent confidence interval:
 -0.7666035 -0.2520935
sample estimates:
       cor 
-0.5613776
```


```
set.seed(324)
bootES(df_quest_deviance[c('score', 'psescore')], R=10000)
```


```
95.00% bca Confidence Interval, 10000 replicates
Stat        CI (Low)    CI (High)   bias        SE          
-0.561      -0.708      -0.298      0.006       0.100
```

## Is it personal familiarity or is it contact?


```
df_quest_contact_avg <-
df_quest %>%
  select(subject, stim, contact) %>%
  spread(stim, contact) %>%
  rowwise() %>%
  mutate(ab=mean(c(a, b)), 
         bc=mean(c(b, c)), 
         ac=mean(c(a, c))) %>%
  select(-a, -b, -c) %>%
  gather(morph_type, contact, -subject)
df_contact_deviance <-
df_quest_deviance %>%
  arrange(subject, morph_type) %>%
  merge(df_quest_contact_avg)
```


Are familiarity score and contact correlated?


```
cor.test(df_contact_deviance$score, df_contact_deviance$contact)
```


```
    Pearson's product-moment correlation

data:  df_contact_deviance$score and df_contact_deviance$contact
t = 2.6517, df = 28, p-value = 0.01304
alternative hypothesis: true correlation is not equal to 0
95 percent confidence interval:
 0.1046298 0.6959508
sample estimates:
      cor 
0.4480113
```


```
set.seed(324)
bootES(df_contact_deviance[c('score', 'contact')], R=10000)
```


```
95.00% bca Confidence Interval, 10000 replicates
Stat        CI (Low)    CI (High)   bias        SE          
0.448       0.168       0.675       -0.002      0.130
```


Run a model that predicts the variance of \(\Delta\text{PSE}\) using only the familiarity score and then also the contact score.


```
print(paste("Log-likelihood ratio:", lr, "pvalue:", plr))
```


```
[1] "Log-likelihood ratio: 6.3024453723185 pvalue: 0.0120571535553395"
```


```
# from http://www.statmethods.net/advstats/bootstrapping.html
bs <- function(formula, data, indices) {
  d <- data[indices,] # allows boot to select sample 
  fit <- lm(formula, data=d)
  return(coef(fit)) 
} 
set.seed(234)
results <- boot(data=df_contact_deviance, statistic=bs,
                R=10000, formula=psescore ~ score + contact)
t0 <- results$t0
conf <- c()
for (i in 1:length(t0)) {
  b <- boot.ci(results, type='bca', index=i)
  conf <- rbind(conf, b$bca[4:5])
}
df_ci <- data.frame(t0=t0, lci=conf[,1], rci=conf[,2])
```


```
df_ci
```


Now compute partial correlations


```
partialout_variable <- function(df, x, y, z) {
  mx <- lm(df[[x]] ~ df[[z]])
  my <- lm(df[[y]] ~ df[[z]])
  df_out <- data.frame(mx$residuals, my$residuals)
  names(df_out) <- c(x, y)
  return(df_out)
}
# Make plots of partial correlation with residuals
partial_contact <- partialout_variable(df_contact_deviance, "psescore", "score", "contact")
partial_score <- partialout_variable(df_contact_deviance, "psescore", "contact", "score")
cor.test(partial_contact$psescore, partial_contact$score)
```


```
    Pearson's product-moment correlation

data:  partial_contact$psescore and partial_contact$score
t = -2.4183, df = 28, p-value = 0.02235
alternative hypothesis: true correlation is not equal to 0
95 percent confidence interval:
 -0.67487275 -0.06515499
sample estimates:
       cor 
-0.4156671
```


```
cor.test(partial_score$psescore, partial_score$contact)
```


```
    Pearson's product-moment correlation

data:  partial_score$psescore and partial_score$contact
t = -2.5585, df = 28, p-value = 0.01621
alternative hypothesis: true correlation is not equal to 0
95 percent confidence interval:
 -0.68771457 -0.08898003
sample estimates:
       cor 
-0.4352951
```


```
set.seed(3242)
bootES(partial_contact[c('psescore', 'score')], R=10000)
```


```
95.00% bca Confidence Interval, 10000 replicates
Stat        CI (Low)    CI (High)   bias        SE          
-0.416      -0.610      -0.158      0.008       0.116
```


```
bootES(partial_score[c('psescore', 'contact')], R=10000)
```


```
95.00% bca Confidence Interval, 10000 replicates
Stat        CI (Low)    CI (High)   bias        SE          
-0.435      -0.619      -0.166      -0.001      0.113
```


```
# add the morph types to partial_contact and partial_score
partial_contact$morph_type <- df_contact_deviance$morph_type
partial_score$morph_type <- df_contact_deviance$morph_type
```


```
# just a theme to uniform scatters
theme_Publication_scatter <- function(base_size=12) {
  (theme_Publication(base_size=base_size) + 
  theme(legend.position=c(0.95, 0.6),
        legend.direction='vertical',
        legend.key.size=unit(.8, 'picas'),
        legend.title=element_text(size=10),
        axis.title=element_text(size=10)))
}
```


```
ggplot(aes(score, psescore, color=morph_type), data=df_quest_deviance) +
  geom_smooth(method='lm', aes(group=1), se=F, color='darkgray') + 
  geom_point() +
  coord_fixed(ratio=10) +
  labs(x='Familiarity score', y=TeX('$\\Delta$PSE variance'), color='Morph type') + 
  theme_Publication_scatter() +
  scale_color_brewer(palette='Set2')
```


```
ggsave('../img/psequest_scatter_psefam.png', width=3, height=3)
```


```
ggplot(aes(score, psescore, color=morph_type), data=partial_contact) +
  geom_smooth(method='lm', aes(group=1), se=F, color='darkgray') + 
  geom_point() +
  coord_fixed(ratio=10) +
  labs(x='Familiarity score | Contact', y=TeX('$\\Delta$PSE variance | Contact'), color='Morph type') + 
  theme_Publication_scatter() +
  scale_color_brewer(palette='Set2')
```


```
ggsave('../img/psequest_scatter_psefam_contact.png', width=3, height=3)
```


```
ggplot(aes(contact, psescore, color=morph_type), data=partial_score) +
  geom_smooth(method='lm', aes(group=1), se=F, color='darkgray') + 
  geom_point() +
  coord_fixed(ratio=10, expand=T) +
  labs(x='Contact score | Familiarity', y=TeX('$\\Delta$PSE variance | Familiarity'), color='Morph type') + 
  theme_Publication_scatter() +
  scale_color_brewer(palette='Set2')
```


```
ggsave('../img/psequest_scatter_psecontact_fam.png', width=3, height=3)
```

# PSE for individual identities

We are going to evaluate the bias towards specific identities, instead of pairs of morphs. To do so, we’ll fit a linear model predicting the PSE, taking the data of each pair of morphs containing the same identity. For example, to evaluate the bias for identity `a`, we’ll consider the PSE estimates for morphs `ab` and `ac` across the two sessions, and fit a linear model with participants, angular location, and their interaction as predictors.


```
require(tidyverse)
require(lme4)
require(car)
require(broom)
```


First we compute the PSE for each participant by adding PSEp and DPSE.


```
cols <- c('pos1', 'pos3', 'pos5', 'pos7')
pse_ses1 = list()
pse_ses2 = list()
# compute the PSE by adding PSEp to DPSE for each subject
for (morph in c('ac', 'ab', 'bc')) {
  psep <- psep_ses1[[morph]]
  dpse <- dpse_ses1[[morph]]
  pse <- (matrix(rep(psep[cols], 10), nrow=10, byrow=T) + 
                       dpse[cols])*100 + 50
  pse$subject <- row.names(pse)
  pse_ses1[[morph]] <- pse
  
  psep <- psep_ses2[[morph]]
  dpse <- dpse_ses2[[morph]]
  pse <- (matrix(rep(psep[cols], 10), nrow=10, byrow=T) + 
                       dpse[cols])*100 + 50
  pse$subject <- row.names(pse)
  pse_ses2[[morph]] <- pse
}
# now store it in long format
pse_ses1_long <-
  ldply(
    lapply(pse_ses1, function(x) mutate(x, subject=rownames(x)) %>% gather(pos, pse, -subject)),
    data.frame) %>%
  mutate(morph_type=.id, session='1')
pse_ses2_long <-
  ldply(
    lapply(pse_ses2, function(x) mutate(x, subject=rownames(x)) %>% gather(pos, pse, -subject)),
    data.frame) %>%
  mutate(morph_type=.id, session='2')
pse <- 
  rbind(pse_ses1_long, pse_ses2_long) %>%
  mutate(pos_num=mapvalues(pos, 
                           paste('pos', c(1, 3, 5, 7), sep=''), 
                           c(1, 3, 5, 7)*45))
pse$pos_num <- factor(pse$pos_num, levels=c(1, 3, 5, 7)*45)
```


Let’s save it for later use.


```
# save pse
pse_fn <- '../derivatives/pse.csv'
if (!file.exists(pse_fn)) {
  pse_ <- pse %>%
    select(subject, session, morph_type, pos_num, pse)
  write_csv(pse_, pse_fn)
}
```


Here we load the pre-estimated data


```
pse <- read_csv('../derivatives/pse.csv', col_types='ciccd')
pse$morph_type <- as.factor(pse$morph_type)
pse$pos_num <- factor(pse$pos_num, 
                      levels=c('45', '135', '225', '315'))
```


We set up a function to filter the data for each individual identity. Moreover, we make sure that the PSE value indicates consistently the bias with respect to the same identity.


```
filter_dataset <- function(id, df) {
  identity2morphs <- list(a=c('ab', 'ac'),
                          b=c('ab', 'bc'),
                          c=c('bc', 'ac'))
  df_ <-
    df %>%
    filter(morph_type %in% identity2morphs[[id]]) %>%
    droplevels(.$morph_type)
  
  # put the PSE in the right order: > 50 indicates 
  # more towards the identity of interest
  if (id == 'b') {
    df_ <-
    df_ %>%
      mutate(pse=ifelse(morph_type == 'ab', 100 - pse, pse))
  } else if (id == 'c') {
    df_ <-
    df_ %>%
      mutate(pse=100 - pse)
  }
  
  return(df_)
}
```


Finally we can fit the models


```
ids <- c('a', 'b', 'c')
# split the datasets
pse_ids <- map(ids, filter_dataset, pse)
fit_model <- function(df) {
  df$subject <- as.factor(df$subject)
  df$pos_num <- as.factor(df$pos_num)
  contrasts(df$subject) <- contr.sum(length(unique(df$subject)))
  contrasts(df$pos_num) <- contr.sum(length(unique(df$pos_num)))
  
  return(lm(pse ~ subject * pos_num, data=df))
}
model_ids <- 
  map(pse_ids, fit_model) %>%
  set_names(ids)
anova_model <-
  model_ids %>%
  map(Anova, type=3)
tidy_anova <-
  anova_model %>%
  map_df(tidy, .id='id')
```


And here are the results


```
anova_model
```


```
$a
Anova Table (Type III tests)

Response: pse
                Sum Sq  Df   F value    Pr(>F)    
(Intercept)     438129   1 1501.3371 < 2.2e-16 ***
subject           1395   9    0.5311    0.8495    
pos_num          18777   3   21.4481  3.43e-11 ***
subject:pos_num   9191  27    1.1664    0.2807    
Residuals        35019 120                        
---
Signif. codes:  0 ‘***’ 0.001 ‘**’ 0.01 ‘*’ 0.05 ‘.’ 0.1 ‘ ’ 1

$b
Anova Table (Type III tests)

Response: pse
                Sum Sq  Df   F value    Pr(>F)    
(Intercept)     469993   1 1184.2655 < 2.2e-16 ***
subject           5970   9    1.6713   0.10322    
pos_num          16226   3   13.6287 1.039e-07 ***
subject:pos_num  18981  27    1.7714   0.01947 *  
Residuals        47624 120                        
---
Signif. codes:  0 ‘***’ 0.001 ‘**’ 0.01 ‘*’ 0.05 ‘.’ 0.1 ‘ ’ 1

$c
Anova Table (Type III tests)

Response: pse
                Sum Sq  Df   F value    Pr(>F)    
(Intercept)     302383   1 1093.0426 < 2.2e-16 ***
subject          10701   9    4.2981 7.363e-05 ***
pos_num          62758   3   75.6183 < 2.2e-16 ***
subject:pos_num  24919  27    3.3361 3.229e-06 ***
Residuals        33197 120                        
---
Signif. codes:  0 ‘***’ 0.001 ‘**’ 0.01 ‘*’ 0.05 ‘.’ 0.1 ‘ ’ 1
```


We find a significant interaction between participants and angular location for identities `b` and `c`, but not for identity `a`, suggesting that for identity `a` the bias is consistently more homogeneous across participants.

## Use the model to predict the biases

We can also use these models now to predict the biases at each angular location for each individual participant.


```
df_pred <- expand.grid(
  subject=unique(pse$subject),
  pos_num=unique(pse$pos_num)
)
df_pred <- 
map_df(model_ids, predict, df_pred) %>%
  gather(identity, pse) %>%
  cbind(df_pred, .)
```


```
ggplot(aes(pos_num, pse-50, color=subject, group=subject), data=df_pred) +
  geom_point() +
  geom_line() +
  facet_wrap(~identity, nrow=1)
```

LS0tCnRpdGxlOiAiRXhwZXJpbWVudCAyIgpvdXRwdXQ6CiAgcGRmX2RvY3VtZW50OgogICAgdG9jOiB5ZXMKICBodG1sX25vdGVib29rOgogICAgdG9jOiB5ZXMKLS0tCgpTdGFydCBieSBsb2FkaW5nIGZ1bmN0aW9ucyBhbmQgZGF0YQpgYGB7ciwgbWVzc2FnZT1GQUxTRSwgd2FybmluZz1GQUxTRX0KcmVxdWlyZShsYXRleDJleHApCnJlcXVpcmUoYm9vdEVTKQojIGxvYWQgcGx5ciAqQkVGT1JFKiBkcGx5ciAoaS5lLiwgdGlkeXZlcnNlKQpyZXF1aXJlKHBseXIpCnJlcXVpcmUodGlkeXZlcnNlKQpyZXF1aXJlKGFzc2VydHRoYXQpCnJlcXVpcmUoYnJvb20pCmRmIDwtIHJlYWRfY3N2KCcuLi9kYXRhL2RhdGEuY3N2JykKCiMgY3JlYXRlIG91dHB1dCBpbWcgZGlyZWN0b3J5IGlmIG5vdCBleGlzdGFudApkaXIuY3JlYXRlKCcuLi9pbWcnLCBzaG93V2FybmluZz1GKQpgYGAKClByZXByb2Nlc3MgdGhlIGRhdGEsIHRoYXQgaXMgZG8gdGhlIGZvbGxvd2luZwoKLSByZW1vdmUgdHJpYWxzIHdoZXJlIHBhcnRpY2lwYW50cyByZXNwb25kZWQgd2l0aCBhIHRoaXJkIG9wdGlvbjsgdGhhdCBpcywgaWYgCnRoZXkgd2VyZSBwcmVzZW50ZWQgd2l0aCBtb3JwaCAkYWIkIGFuZCB0aGV5IHJlc3BvbmRlZCB3aXRoICRjJCwgdGhhdCB0cmlhbAp3aWxsIGJlIHJlbW92ZWQKLSBiaW5hcml6ZSB0aGUgcmVzcG9uc2VzIHRvIHJ1biB0aGUgbW9kZWwKCmBgYHtyLCBtZXNzYWdlPUZBTFNFLCB3YXJuaW5nPUZBTFNFfQpkZiA8LSBkZiAlPiUKICBmaWx0ZXIodGhpcmRfb3B0aW9uICE9IDEpCgojIG5vdyB3ZSBuZWVkIHRvIGFkZCBhIGJpbmFyaXplZCByZXNwb25zZSB0byBjb21wdXRlIHRoZSBwc3ljaG9tZXRyaWMgY3VydmVzCiMgYnV0IHRoaXMgY2hhbmdlcyBkZXBlbmRpbmcgb24gbW9ycGhfdHlwZSwgc28gd2UgbmVlZCB0byBtYWtlIGEgZnVuY3Rpb24gdGhhdAojIGNoZWNrcyB0aGUgY29uZGl0aW9ucwpiaW5hcml6ZV9yZXNwb25zZSA8LSBmdW5jdGlvbihtb3JwaF90eXBlLCByZXNwb25zZV9pZGVudGl0eSkgewogIGlmIChtb3JwaF90eXBlID09ICdhYicpIHsKICAgIHJldHVybihpZmVsc2UocmVzcG9uc2VfaWRlbnRpdHkgPT0gJ2EnLCAwLCAxKSkKICB9IGVsc2UgaWYgKG1vcnBoX3R5cGUgPT0gJ2JjJykgewogICAgcmV0dXJuKGlmZWxzZShyZXNwb25zZV9pZGVudGl0eSA9PSAnYicsIDAsIDEpKQogIH0gZWxzZSBpZiAobW9ycGhfdHlwZSA9PSAnYWMnKSB7CiAgICByZXR1cm4oaWZlbHNlKHJlc3BvbnNlX2lkZW50aXR5ID09ICdhJywgMCwgMSkpCiAgfQp9CgpkZiA8LQpkZiAlPiUKICByb3d3aXNlKCkgJT4lCiAgbXV0YXRlKHJlc3BvbnNlX2Jpbj1iaW5hcml6ZV9yZXNwb25zZShtb3JwaF90eXBlLCByZXNwb25zZV9pZGVudGl0eSkpCgpkZiRwb3MgPC0gYXMuZmFjdG9yKGRmJHBvcykKZGYkc2Vzc2lvbiA8LSBhcy5mYWN0b3IoZGYkc2Vzc2lvbikKCiMgbWFrZSBzdXJlIHRoYXQgYmluYXJpemVfcmVzcG9uc2Ugd29ya2VkLCBpLmUuIHdlIHNob3VsZCBnZXQgMXMgYW5kIDBzIGZvciBlYWNoCiMgbW9ycGhfdHlwZQpjaGVja18gPC0KZGYgJT4lIAogIGdyb3VwX2J5KG1vcnBoX3R5cGUpICU+JSAKICBzdW1tYXJpc2UoY2hlY2s9bGVuZ3RoKHVuaXF1ZShyZXNwb25zZV9iaW4pKSkKYXNzZXJ0X3RoYXQobGVuZ3RoKGNoZWNrXyRjaGVjaykgPT0gMykKYXNzZXJ0X3RoYXQoYWxsKGNoZWNrXyRjaGVjayA9PSBjKDIsIDIsIDIpKSkKYGBgCiMgUHJvY2VkdXJlIGFuZCBlcXVhdGlvbnMKV2UgYXJlIGdvaW5nIHRvIGZpdCBhIGxpbmVhciBtaXhlZCBlZmZlY3QgbW9kZWwgdG8gdGhlIGRhdGEuIFdlIHdpbGwgbW9kZWwgdGhlIGRhdGEgYXMgZm9sbG93cwoKJCQKeV5rID0gXHRleHR7bG9naXR9XGxlZnQoZyh4KVxyaWdodClcXApnKHgpID0gXGJldGFfMCB4ICsgXHN1bV97aT0xfV44XGxlZnQoXGJldGFfaSArIHpfaV5rXHJpZ2h0KUlfaQokJApXaGVyZSAkeV5rJCBpcyB0aGUgcmVzcG9uc2UgZm9yIHN1YmplY3QgJGskLCAkeCQgaXMgdGhlIChzY2FsZWQpIHBlcmNlbnRhZ2Ugb2YgbW9ycGhpbmcsCiRcYmV0YV9pLCBpPTEuLi40JCBhcmUgdGhlIGZpeGVkLWVmZmVjdHMgZm9yIGVhY2ggYW5ndWxhciBsb2NhdGlvbiAoMCB0byAzMTUgaW4gNDUgZGVnIHN0ZXBzKSwgCmFuZCAkel9pXmskIGFyZSB0aGUgcmFuZG9tLWVmZmVjdHMgKHJhbmRvbSBzbG9wZXMgZm9yIGxvY2F0aW9uKSAKZm9yIGVhY2ggc3ViamVjdCwgYW5kICRJX2kkIGlzIGFuIGluZGljYXRvciB2YXJpYWJsZSwgaW5kaWNhdGluZyB0aGUgYW5ndWxhciBsb2NhdGlvbiBmb3IgZWFjaCB0cmlhbC4KCkluIHRoaXMgd2F5IGZvciBlYWNoIHN1YmplY3Qgd2UgY2FuIGZpbmQgdGhlIFBTRSBhcyB0aGUgcG9pbnQgd2hlcmUgJHleayA9IDAuNSQsIHRoYXQgaXMgdGhlIHBvaW50ICRcaGF0e3h9JAoKJCQKXHRleHR7bG9naXR9KGcoXGhhdHt4fSkpID0gMC41IFxMb25nbGVmdHJpZ2h0YXJyb3cKZyhcaGF0e3h9KSA9IDAgXExvbmdsZWZ0cmlnaHRhcnJvdyBcXApcYmV0YV8wIFxoYXR7eH0gKyBcc3VtX3tpPTF9XjRcbGVmdChcYmV0YV9pICsgel9pXmtccmlnaHQpSV9pID0gMCBcTG9uZ2xlZnRyaWdodGFycm93XFwKXGhhdHt4fSA9IC1cZnJhY3tcc3VtX3tpPTF9XjRcbGVmdChcYmV0YV9pICsgel9pXmtccmlnaHQpSV9pfXtcYmV0YV8wfQokJAoKVGh1cyBmb3IgZXZlcnkgYW5ndWxhciBsb2NhdGlvbiAkaSQgd2UgaGF2ZSB0aGF0IAoKJCQKXGhhdHt4fV9pID0gLVxmcmFje1xiZXRhX2l9e1xiZXRhXzB9IC0gXGZyYWN7el9pXmt9e1xiZXRhXzB9ID0gXHRleHR7UFNFfV5wX2kgKyBcRGVsdGFcdGV4dHtQU0V9X2lec1xcCiQkCgp3aXRoICRcdGV4dHtQU0V9X2lecCQgYmVpbmcgdGhlIHBvcHVsYXRpb24tbGV2ZWwgUFNFCmF0IGxvY2F0aW9uICRpJCwgYW5kICRcRGVsdGFcdGV4dHtQU0V9X2lecyQgYmVpbmcgdGhlIGNoYW5nZSBhdCBsb2NhdGlvbiAkaSQKZm9yIHN1YmplY3QgJHMkLiAKCldlIHdpbGwgZml0IG9uZSBzdWNoIG1vZGVsIGZvciBlYWNoIG9mIHRoZSBtb3JwaCB0eXBlcywgYW5kIG9uZQpmb3IgZWFjaCBzZXNzaW9uLgoKIyBNb2RlbCBmaXR0aW5nCmBgYHtyLCBtZXNzYWdlPUZBTFNFLCB3YXJuaW5nPUZBTFNFfQpyZXF1aXJlKGxtZTQpCgojIGRlZmluZSBzb21lIGZ1bmN0aW9ucwpleHRyYWN0X21vcnBoX3Nlc3Npb24gPC0gZnVuY3Rpb24oZGYsIG10LCBzZXMpIHsKICAjIEV4dHJhY3QgdHJpYWxzIGZyb20gb25lIHBhcnRpY3VsYXIgbW9ycGhfdHlwZSBhbmQgc2Vzc2lvbgogIGRmXyA8LQogICAgZGYgJT4lCiAgICBmaWx0ZXIobW9ycGhfdHlwZSA9PSBtdCwgc2Vzc2lvbiA9PSBzZXMpICU+JQogICAgbXV0YXRlKG1vcnBoX3Jlc2M9KG1vcnBoIC0gNTApLzEwMCkKICByZXR1cm4oZGZfKQp9CnJ1bl9tb2RlbF9zZXNzaW9uIDwtIGZ1bmN0aW9uKGRmKSB7CiAgIyBSdW4gdGhlIGZvbGxvd2luZyBsb2dpdCBtaXhlZC1lZmZlY3QgbW9kZWwgZm9yIG9uZSBzZXNzaW9uCiAgIyByZXNwb25zZV9iaW4gfiBtb3JwaF9yZXNjICsgcG9zIC0gMSArIChwb3MgLSAxIHwgc3ViamVjdCkKICBtIDwtIGdsbWVyKHJlc3BvbnNlX2JpbiB+IG1vcnBoX3Jlc2MgKyBwb3MgLSAxICsgKHBvcyAtIDEgfCBzdWJqZWN0KSwgCiAgICAgICAgICAgZGF0YT1kZiwgCiAgICAgICAgICAgZmFtaWx5PWJpbm9taWFsKGxpbms9J2xvZ2l0JyksIAogICAgICAgICAgIGNvbnRyb2w9Z2xtZXJDb250cm9sKG9wdGltaXplcj0nYm9ieXFhJywgb3B0Q3RybD1saXN0KG1heGZ1bj0yMDAwMCkpKQogIHJldHVybihtKQp9CmBgYAoKUnVuIHRoZSBtb2RlbCBzZXBhcmF0ZWx5IGZvciBldmVyeSBtb3JwaApgYGB7cn0KbW9ycGhzIDwtIHVuaXF1ZShkZiRtb3JwaF90eXBlKQpkZl9zZXMxIDwtIHNhcHBseShtb3JwaHMsIGZ1bmN0aW9uKHgpIGV4dHJhY3RfbW9ycGhfc2Vzc2lvbihkZiwgeCwgJzEnKSwgCiAgICAgICAgICAgICAgICAgIHNpbXBsaWZ5PUYsIFVTRS5OQU1FUz1UKSAKZGZfc2VzMiA8LSBzYXBwbHkobW9ycGhzLCBmdW5jdGlvbih4KSBleHRyYWN0X21vcnBoX3Nlc3Npb24oZGYsIHgsICcyJyksIAogICAgICAgICAgICAgICAgICBzaW1wbGlmeT1GLCBVU0UuTkFNRVM9VCkgCiMgY2hlY2sgdGhhdCB3ZSBoYXZlIGFsbCB0aGUgdHJpYWxzCmFzc2VydF90aGF0KHN1bShzYXBwbHkoZGZfc2VzMSwgbnJvdykpICsgc3VtKHNhcHBseShkZl9zZXMyLCBucm93KSkgPT0gbnJvdyhkZikpCiMgbm93IGNvbXB1dGUgbW9kZWxzCm1vZGVsX3NlczEgPC0gc2FwcGx5KGRmX3NlczEsIHJ1bl9tb2RlbF9zZXNzaW9uLCBzaW1wbGlmeT1GLCBVU0UuTkFNRVM9VCkKbW9kZWxfc2VzMiA8LSBzYXBwbHkoZGZfc2VzMiwgcnVuX21vZGVsX3Nlc3Npb24sIHNpbXBsaWZ5PUYsIFVTRS5OQU1FUz1UKQpgYGAKClRoZSBmb2xsb3dpbmcgZnVuY3Rpb25zIGFyZSB1c2VkIHRvIGV4dHJhY3QgYm90aCB0aGUgcG9wdWxhdGlvbiAkXHRleHR7UFNFfV5wJCBhbmQgdGhlIHN1YmplY3QgJFxEZWx0YVx0ZXh0e1BTRX1ecyQuIApSZW1lbWJlciB0aGF0ICRcdGV4dHtQU0V9XnMgPSBcdGV4dHtQU0V9XnAgKyBcRGVsdGFcdGV4dHtQU0V9XnMkLgpgYGB7cn0KcG9wdWxhdGlvbl9wc2UgPC0gZnVuY3Rpb24obW9kZWwpIHsKICAjIENvbXB1dGVzIHBvcHVsYXRpb24tbGV2ZWwgUFNFIChzZWUgZXF1YXRpb25zIGFib3ZlKQogICMgTm90ZTogdGhleSBhcmUgb24gdGhlIHNjYWxlIG9mIG1vcnBoX3Jlc2MKICBtb3JwaF9iZXRhIDwtIGZpeGVmKG1vZGVsKVsxXQogIHBvc19iZXRhcyA8LSBmaXhlZihtb2RlbClbLTFdCiAgcmV0dXJuKC1wb3NfYmV0YXMvbW9ycGhfYmV0YSkKfQoKc3ViamVjdHNfcHNlIDwtIGZ1bmN0aW9uKG1vZGVsKSB7CiAgIyBDb21wdXRlcyBzdWJqZWN0LWxldmVsIFBTRSAoc2VlIGVxdWF0aW9ucyBhYm92ZSkKICBtb3JwaF9wb3AgPC0gZml4ZWYobW9kZWwpWzFdCiAgcG9zaXRpb25fcG9wIDwtIGZpeGVmKG1vZGVsKVstMV0KICByYW5lZl9tb2RlbCA8LSByYW5lZihtb2RlbCkkc3ViamVjdAogICMgdGhpcyBpcyB0aGUgZGVub21pbmF0b3IKICBtb3JwaF9zdWJqIDwtIHJhbmVmX21vZGVsWywgMV0gKyBtb3JwaF9wb3AKICAjIHRoZXNlIGFyZSB0aGUgbnVtZXJhdG9ycwogIHBvc2l0aW9uX3N1YmogPC0gcmFuZWZfbW9kZWxbLCAtMV0gIAogICMgZXh0ZW5kIHBvc2l0aW9uX2JldGEgdG8gZ2V0IHRoZSBzYW1lIHNoYXBlIGFzIHJhbmVmX3BvcwogIHBvc2l0aW9uX3BvcCA8LSBtYXRyaXgocmVwKHBvc2l0aW9uX2JldGEsIG5yb3cocmFuZWZfcG9zKSksIAogICAgICAgICAgICAgICAgICAgICAgICAgIGJ5cm93PVQsIG5yb3c9bnJvdyhyYW5lZl9wb3MpKQogIHBvc2l0aW9uX3N1YmogPC0gcG9zaXRpb25fc3ViaiArIHBvc2l0aW9uX3BvcAogICMgZXh0ZW5kIG1vcnBoX3N1YmogdG8gZ2V0IHRoZSBzYW1lIHNoYXBlIGFzIHBvc2l0aW9uX3N1YmoKICBtb3JwaF9zdWJqIDwtIG1hdHJpeChyZXAobW9ycGhfc3ViaiwgbmNvbChwb3NpdGlvbl9zdWJqKSksIG5jb2w9bmNvbChwb3NpdGlvbl9zdWJqKSkgCiAgcmV0dXJuKC1wb3NpdGlvbl9zdWJqL21vcnBoX3N1YmopCn0KCmRlbHRhX3BzZSA8LSBmdW5jdGlvbihtb2RlbCkgewogICMgQ29tcHV0ZXMgc3ViamVjdC1sZXZlbCBQU0UgKHNlZSBlcXVhdGlvbnMgYWJvdmUpCiAgIyBOb3RlOiB0aGV5IGFyZSBvbiB0aGUgc2NhbGUgb2YgbW9ycGhfcmVzYwogICMgSUYgJ21vcnBoX3Jlc2MnIGlzIGVudGVyZWQgYXMgYSByYW5kb20gZWZmZWN0LCBhZGQgdGhhdCBmb3IgZWFjaCBpbmRpdmlkdWFsCiAgIyBtb3JwaAogIG1vcnBoX2JldGEgPC0gZml4ZWYobW9kZWwpWzFdCiAgcmFuZWZfbW9kZWwgPC0gcmFuZWYobW9kZWwpJHN1YmplY3QKICBpZiAoJ21vcnBoX3Jlc2MnICVpbiUgbmFtZXMocmFuZWZfbW9kZWwpKSB7CiAgICBwc2VfcG9wIDwtIHBvcHVsYXRpb25fcHNlKG1vZGVsKQogICAgcHNlX3N1YmogPC0gc3ViamVjdHNfcHNlKG1vZGVsKQogICAgcHNlX3BvcCA8LSBtYXRyaXgocmVwKHBzZV9wb3AsIG5yb3cocHNlX3N1YmopKSwgbnJvdz1ucm93KHBzZV9zdWJqKSwgYnlyb3c9VCkKICAgIHJldHVybihwc2Vfc3ViaiAtIHBzZV9wb3ApIAogIH0gZWxzZSB7CiAgICByZXR1cm4oLXJhbmVmX21vZGVsL21vcnBoX2JldGEpCiAgfQp9CmBgYAoKTGV0J3MgbG9vayBhdCB0aGUgcG9wdWxhdGlvbiBlc3RpbWF0ZXMgZm9yIHRoZSBQU0VzIGFjcm9zcyB0aGUgdHdvCnNlc3Npb25zLCBhcyB3ZWxsIGFzIHRoZSBzdWJqZWN0LWxldmVsIGVzdGltYXRlcy4KCmBgYHtyfQpwc2VwX3NlczEgPC0gc2FwcGx5KG1vZGVsX3NlczEsIHBvcHVsYXRpb25fcHNlLCBzaW1wbGlmeT1GLCBVU0UuTkFNRVM9VCkKcHNlcF9zZXMyIDwtIHNhcHBseShtb2RlbF9zZXMyLCBwb3B1bGF0aW9uX3BzZSwgc2ltcGxpZnk9RiwgVVNFLk5BTUVTPVQpCmRwc2Vfc2VzMSA8LSBzYXBwbHkobW9kZWxfc2VzMSwgZGVsdGFfcHNlLCBzaW1wbGlmeT1GLCBVU0UuTkFNRVM9VCkKZHBzZV9zZXMyIDwtIHNhcHBseShtb2RlbF9zZXMyLCBkZWx0YV9wc2UsIHNpbXBsaWZ5PUYsIFVTRS5OQU1FUz1UKQpgYGAKCiMjIFByZWRpY3QgcHN5Y2hvbWV0cmljIGN1cnZlcwpMZXQncyBwbG90IHRoZSBwb3B1bGF0aW9uIGVzdGltYXRlcyBmaXJzdApgYGB7cn0KZGZfcHJlZGljdCA8LQogIGV4cGFuZC5ncmlkKG1vcnBoX3Jlc2M9c2VxKC0wLjUsIDAuNSwgMC4wMSksIHBvcz1jKCcxJywgJzMnLCAnNScsICc3JykpCgpwcmVkaWN0X3BvcF9zZXMxIDwtIHNhcHBseShtb2RlbF9zZXMxLCBwcmVkaWN0LCBuZXdkYXRhPWRmX3ByZWRpY3QsIAogICAgICAgICAgICAgICAgICAgICAgICAgICByZS5mb3JtPU5BLCB0eXBlPSdyZXNwb25zZScsIHNpbXBsaWZ5PUYsIFVTRS5OQU1FUz1UKQpwcmVkaWN0X3BvcF9zZXMyIDwtIHNhcHBseShtb2RlbF9zZXMyLCBwcmVkaWN0LCBuZXdkYXRhPWRmX3ByZWRpY3QsIAogICAgICAgICAgICAgICAgICAgICAgICAgICByZS5mb3JtPU5BLCB0eXBlPSdyZXNwb25zZScsIHNpbXBsaWZ5PUYsIFVTRS5OQU1FUz1UKQoKIyBhZGQgZGZfcHJlZGljdCB0byBlYWNoIG9mIHRoZW0KcHJlZGljdF9wb3Bfc2VzMSA8LSBsYXBwbHkocHJlZGljdF9wb3Bfc2VzMSwgZnVuY3Rpb24oeCkgY2JpbmQoZGZfcHJlZGljdCwgcHJlZD14KSkKcHJlZGljdF9wb3Bfc2VzMiA8LSBsYXBwbHkocHJlZGljdF9wb3Bfc2VzMiwgZnVuY3Rpb24oeCkgY2JpbmQoZGZfcHJlZGljdCwgcHJlZD14KSkKCnByZWRpY3RfcG9wX3NlczEgPC0gbGRwbHkocHJlZGljdF9wb3Bfc2VzMSwgZGF0YS5mcmFtZSkKcHJlZGljdF9wb3Bfc2VzMiA8LSBsZHBseShwcmVkaWN0X3BvcF9zZXMyLCBkYXRhLmZyYW1lKQpwcmVkaWN0X3BvcF9zZXMxJHNlc3Npb24gPC0gJzEnCnByZWRpY3RfcG9wX3NlczIkc2Vzc2lvbiA8LSAnMicKCnByZWRpY3RfcG9wIDwtIHJiaW5kKHByZWRpY3RfcG9wX3NlczEsIHByZWRpY3RfcG9wX3NlczIpCnByZWRpY3RfcG9wIDwtIAogIHByZWRpY3RfcG9wICU+JQogIG11dGF0ZShtb3JwaD1tb3JwaF9yZXNjKjEwMCAgKyA1MCwgCiAgICAgICAgIHBvc19udW09YXMubnVtZXJpYyhhcy5jaGFyYWN0ZXIocG9zKSkqNDUsCiAgICAgICAgIG1vcnBoX3R5cGU9LmlkKQpgYGAKCmBgYHtyfQojIG1vZGlmaWVkIGZyb20gaHR0cHM6Ly9ycHVicy5jb20vS291bmR5LzcxNzkyCnRoZW1lX1B1YmxpY2F0aW9uIDwtIGZ1bmN0aW9uKGJhc2Vfc2l6ZT0xMikgewogICAgICBsaWJyYXJ5KGdndGhlbWVzKQogICAgICAodGhlbWVfZm91bmRhdGlvbihiYXNlX3NpemU9YmFzZV9zaXplKQogICAgICAgKyB0aGVtZShwbG90LnRpdGxlID0gZWxlbWVudF90ZXh0KGZhY2UgPSAiYm9sZCIsCiAgICAgICAgICAgICAgICAgICAgICAgICAgICAgICAgICAgICAgICAgc2l6ZSA9IHJlbCgxLjIpLCBoanVzdCA9IDAuNSksCiAgICAgICAgICAgICAgIHRleHQgPSBlbGVtZW50X3RleHQoKSwKICAgICAgICAgICAgICAgcGFuZWwuYmFja2dyb3VuZCA9IGVsZW1lbnRfcmVjdChjb2xvdXIgPSBOQSksCiAgICAgICAgICAgICAgIHBsb3QuYmFja2dyb3VuZCA9IGVsZW1lbnRfcmVjdChjb2xvdXIgPSBOQSksCiAgICAgICAgICAgICAgIHBhbmVsLmJvcmRlciA9IGVsZW1lbnRfcmVjdChjb2xvdXIgPSBOQSksCiAgICAgICAgICAgICAgIGF4aXMudGl0bGUgPSBlbGVtZW50X3RleHQoc2l6ZSA9IHJlbCgxKSksCiAgICAgICAgICAgICAgIGF4aXMudGl0bGUueSA9IGVsZW1lbnRfdGV4dChhbmdsZT05MCx2anVzdCA9MiksCiAgICAgICAgICAgICAgIGF4aXMudGl0bGUueCA9IGVsZW1lbnRfdGV4dCh2anVzdCA9IC0wLjIpLAogICAgICAgICAgICAgICBheGlzLnRleHQgPSBlbGVtZW50X3RleHQoKSwgCiAgICAgICAgICAgICAgIGF4aXMubGluZSA9IGVsZW1lbnRfbGluZShjb2xvdXI9ImJsYWNrIiksCiAgICAgICAgICAgICAgIGF4aXMudGlja3MgPSBlbGVtZW50X2xpbmUoKSwKICAgICAgICAgICAgICAgcGFuZWwuZ3JpZC5tYWpvciA9IGVsZW1lbnRfYmxhbmsoKSwgI2VsZW1lbnRfbGluZShjb2xvdXI9IiNmMGYwZjAiKSwKICAgICAgICAgICAgICAgcGFuZWwuZ3JpZC5taW5vciA9IGVsZW1lbnRfYmxhbmsoKSwKICAgICAgICAgICAgICAgbGVnZW5kLmtleSA9IGVsZW1lbnRfcmVjdChjb2xvdXIgPSBOQSksCiAgICAgICAgICAgICAgIGxlZ2VuZC5wb3NpdGlvbiA9ICJib3R0b20iLAogICAgICAgICAgICAgICBsZWdlbmQuZGlyZWN0aW9uID0gImhvcml6b250YWwiLAogICAgICAgICAgICAgICAjbGVnZW5kLmtleS5zaXplPSB1bml0KDAuMiwgImNtIiksCiAgICAgICAgICAgICAgIGxlZ2VuZC5zcGFjaW5nID0gdW5pdCgwLCAiY20iKSwKICAgICAgICAgICAgICAgbGVnZW5kLnRpdGxlID0gZWxlbWVudF90ZXh0KCksCiAgICAgICAgICAgICAgIHBsb3QubWFyZ2luID0gdW5pdChjKDEwLDUsNSw1KSwibW0iKSwKICAgICAgICAgICAgICAgc3RyaXAuYmFja2dyb3VuZCA9IGVsZW1lbnRfcmVjdChjb2xvdXI9IiNmMGYwZjAiLGZpbGw9IiNmMGYwZjAiKSwKICAgICAgICAgICAgICAgc3RyaXAudGV4dCA9IGVsZW1lbnRfdGV4dChmYWNlPSJib2xkIiksCiAgICAgICAgICAgICAgIHN0cmlwLnRleHQueSA9IGVsZW1lbnRfdGV4dChhbmdsZSA9IDApCiAgICAgICAgICApKQogICAgICAKfQpgYGAKYGBge3IsIGZpZy5oZWlnaHQ9NiwgZmlnLndpZHRoPTh9CmRmIDwtCiAgZGYgJT4lCiAgbXV0YXRlKHBvc19udW09YXMubnVtZXJpYyhhcy5jaGFyYWN0ZXIocG9zKSkqNDUpCgpwcmVkaWN0X3BvcCAlPiUKICBnZ3Bsb3QoYWVzKG1vcnBoLCBwcmVkKjEwMCwgY29sb3I9c2Vzc2lvbikpICsKICBnZW9tX2xpbmUoKSArCiAgZ2VvbV9obGluZSh5aW50ZXJjZXB0PTUwLCBzaXplPTAuNSwgY29sb3I9J2RhcmtncmF5JywgYWxwaGE9MSwgbGluZXR5cGU9J2Rhc2hlZCcpICsKICBnZW9tX3ZsaW5lKHhpbnRlcmNlcHQ9NTAsIHNpemU9MC41LCBjb2xvcj0nZGFya2dyYXknLCBhbHBoYT0xLCBsaW5ldHlwZT0nZGFzaGVkJykgKwogICMgYWRkIGluZGl2aWR1YWwgZGF0YQogIHN0YXRfc3VtbWFyeShkYXRhPWRmLCAKICAgICAgICAgICAgICAgYWVzKG1vcnBoLCByZXNwb25zZV9iaW4pLCAKICAgICAgICAgICAgICAgZnVuLnk9ZnVuY3Rpb24oeCkgc3VtKHgpL2xlbmd0aCh4KSoxMDAsIGdlb209J3BvaW50JykgKwogIGZhY2V0X2dyaWQobW9ycGhfdHlwZSB+IHBvc19udW0pICsKICBsYWJzKHg9J1BlcmNlbnRhZ2UgbW9ycGhpbmcnLCB5PSdQb3B1bGF0aW9uIHByZWRpY3Rpb25cbnBlcmNlbnQgcmVzcG9uc2VzIHRvIHNlY29uZCBpZGVudGl0eScsIGNvbG9yPSdTZXNzaW9uJykgKwogIHNjYWxlX2NvbG9yX2JyZXdlcihwYWxldHRlPSdTZXQxJykgKwogIHRoZW1lX1B1YmxpY2F0aW9uKCkgKyAKICBjb29yZF9lcXVhbCgpCgpnZ3NhdmUoJy4uL2ltZy9wcmVkX3BvcF9nbW0ucG5nJywgd2lkdGg9OCwgaGVpZ2h0PTYpCmBgYAoKTm93IHdlIGNhbiBwcmVkaWN0IGZvciBlYWNoIGluZGl2aWR1YWwgc3ViamVjdApgYGB7cn0KZGZfcHJlZGljdCA8LQogIGV4cGFuZC5ncmlkKAogICAgbW9ycGhfcmVzYz1zZXEoLTAuNSwgMC41LCAwLjAxKSwgCiAgICBwb3M9YygnMScsICczJywgJzUnLCAnNycpLAogICAgc3ViamVjdD11bmlxdWUoZGYkc3ViamVjdCkpCgpwcmVkaWN0X3N1Ympfc2VzMSA8LSBzYXBwbHkobW9kZWxfc2VzMSwgcHJlZGljdCwgbmV3ZGF0YT1kZl9wcmVkaWN0LCAKICAgICAgICAgICAgICAgICAgICAgICAgICAgdHlwZT0ncmVzcG9uc2UnLCBzaW1wbGlmeT1GLCBVU0UuTkFNRVM9VCkKcHJlZGljdF9zdWJqX3NlczIgPC0gc2FwcGx5KG1vZGVsX3NlczIsIHByZWRpY3QsIG5ld2RhdGE9ZGZfcHJlZGljdCwgCiAgICAgICAgICAgICAgICAgICAgICAgICAgIHR5cGU9J3Jlc3BvbnNlJywgc2ltcGxpZnk9RiwgVVNFLk5BTUVTPVQpCgojIGFkZCBkZl9wcmVkaWN0IHRvIGVhY2ggb2YgdGhlbQpwcmVkaWN0X3N1Ympfc2VzMSA8LSBsYXBwbHkocHJlZGljdF9zdWJqX3NlczEsIGZ1bmN0aW9uKHgpIGNiaW5kKGRmX3ByZWRpY3QsIHByZWQ9eCkpCnByZWRpY3Rfc3Vial9zZXMyIDwtIGxhcHBseShwcmVkaWN0X3N1Ympfc2VzMiwgZnVuY3Rpb24oeCkgY2JpbmQoZGZfcHJlZGljdCwgcHJlZD14KSkKCnByZWRpY3Rfc3Vial9zZXMxIDwtIGxkcGx5KHByZWRpY3Rfc3Vial9zZXMxLCBkYXRhLmZyYW1lKQpwcmVkaWN0X3N1Ympfc2VzMiA8LSBsZHBseShwcmVkaWN0X3N1Ympfc2VzMiwgZGF0YS5mcmFtZSkKcHJlZGljdF9zdWJqX3NlczEkc2Vzc2lvbiA8LSAnMScKcHJlZGljdF9zdWJqX3NlczIkc2Vzc2lvbiA8LSAnMicKCnByZWRpY3Rfc3ViaiA8LSByYmluZChwcmVkaWN0X3N1Ympfc2VzMSwgcHJlZGljdF9zdWJqX3NlczIpCnByZWRpY3Rfc3ViaiA8LSAKICBwcmVkaWN0X3N1YmogJT4lCiAgbXV0YXRlKG1vcnBoPW1vcnBoX3Jlc2MqMTAwICArIDUwLCAKICAgICAgICAgcG9zX251bT1hcy5udW1lcmljKGFzLmNoYXJhY3Rlcihwb3MpKSo0NSwKICAgICAgICAgbW9ycGhfdHlwZT0uaWQpCmBgYAoKTm93IHdlIGNhbiBzYXZlIGVhY2ggaW5kaXZpZHVhbCBwbG90IHRvIGRpc2sKCmBgYHtyLCBmaWcuaGVpZ2h0PTYsIGZpZy53aWR0aD04fQpzdWJqZWN0cyA8LSB1bmlxdWUoZGYkc3ViamVjdCkKZm9yIChzdWJqIGluIHN1YmplY3RzKSB7CiAgb3V0X2RpciA8LSBmaWxlLnBhdGgoJy4uL2ltZycsICdwcmVkX2dtbScpCiAgZm5vdXQgPC0gZmlsZS5wYXRoKG91dF9kaXIsIHBhc3RlKHN1YmosICdfcHJlZF9nbW0ucG5nJywgc2VwPScnKSkKICAjIHNldHVwIGRhdGFmcmFtZXMgZm9yIHBsb3R0aW5nCiAgdGhpc19zdWJqZWN0X2RmIDwtIGRmICU+JSAKICAgIGZpbHRlcihzdWJqZWN0ID09IHN1YmopICU+JQogICAgIyBhZGQgcG9zaXRpb24gaW4gYW5nbGVzCiAgICBtdXRhdGUocG9zX251bT1hcy5udW1lcmljKGFzLmNoYXJhY3Rlcihwb3MpKSo0NSkKICB0aGlzX3ByZWRpY3Rfc3ViaiA8LSBwcmVkaWN0X3N1YmogJT4lIAogICAgZmlsdGVyKHN1YmplY3QgPT0gc3ViaikgJT4lCiAgICAjIGFkZCBwb3NpdGlvbiBpbiBhbmdsZXMKICAgIG11dGF0ZShwb3NfbnVtPWFzLm51bWVyaWMoYXMuY2hhcmFjdGVyKHBvcykpKjQ1KQogIAogIHBsb3RfY3VydmUgPC0KICAgIHRoaXNfcHJlZGljdF9zdWJqICU+JQogICAgIyBhZGQgcHJlZGljdGlvbgogICAgZ2dwbG90KGFlcyhtb3JwaCwgcHJlZCoxMDAsIGNvbG9yPXNlc3Npb24pKSArCiAgICBnZW9tX2hsaW5lKHlpbnRlcmNlcHQ9NTAsIHNpemU9MC41LCBjb2xvcj0nZGFya2dyYXknLCBhbHBoYT0xLCBsaW5ldHlwZT0nZGFzaGVkJykgKwogICAgZ2VvbV92bGluZSh4aW50ZXJjZXB0PTUwLCBzaXplPTAuNSwgY29sb3I9J2RhcmtncmF5JywgYWxwaGE9MSwgbGluZXR5cGU9J2Rhc2hlZCcpICsKICAgIGdlb21fbGluZSgpICsKICAgICMgYWRkIGluZGl2aWR1YWwgZGF0YQogICAgc3RhdF9zdW1tYXJ5KGRhdGE9dGhpc19zdWJqZWN0X2RmLCAKICAgICAgICAgICAgICAgICBhZXMobW9ycGgsIHJlc3BvbnNlX2JpbiksIAogICAgICAgICAgICAgICAgIGZ1bi55PWZ1bmN0aW9uKHgpIHN1bSh4KS9sZW5ndGgoeCkqMTAwLCBnZW9tPSdwb2ludCcpICsKICAgIGZhY2V0X2dyaWQobW9ycGhfdHlwZSB+IHBvc19udW0pICsKICAgIGxhYnMoeD0nUGVyY2VudGFnZSBtb3JwaGluZycsIHk9J1BlcmNlbnQgcmVzcG9uc2VzIHRvIHNlY29uZCBpZGVudGl0eScsIGNvbG9yPSdTZXNzaW9uJykgKwogICAgc2NhbGVfY29sb3JfYnJld2VyKHBhbGV0dGU9J1NldDEnKSArCiAgICBnZ3RpdGxlKHBhc3RlKCJTdWJqZWN0Iiwgc3ViaikpICsKICAgIHRoZW1lX1B1YmxpY2F0aW9uKCkgKwogICAgY29vcmRfZXF1YWwoKSAKICAKICAgICMgc2F2ZQogICAgZGlyLmNyZWF0ZShvdXRfZGlyLCByZWN1cnNpdmU9VCkKICAgIGdnc2F2ZShmaWxlbmFtZT1mbm91dCwgcGxvdD1wbG90X2N1cnZlLCB3aWR0aD04LCBoZWlnaHQ9NikKfQpgYGAKClRoZXNlIGFyZSB0aGUgcG9wdWxhdGlvbi1sZXZlbCBlc3RpbWF0ZXMKYGBge3IsIGVjaG89RkFMU0V9CiMgbGV0J3MgbWFrZSBhIGRhdGFmcmFtZSBmb3IgcGxvdHRpbmcKbWFrZV9wc2VwX2RmIDwtIGZ1bmN0aW9uKGxpc3RfZGYpIHsKICBkZl8gPC0gZGF0YS5mcmFtZSh0KGRhdGEuZnJhbWUobGlzdF9kZikpKQogIGRmXyRtb3JwaF90eXBlIDwtIHJvdy5uYW1lcyhkZl8pCiAgIyBtYWtlIGl0IGxvbmcKICBkZl8gPC0gZGZfICU+JQogICAgZ2F0aGVyKHBvcywgcHNlLCAtbW9ycGhfdHlwZSkgJT4lCiAgICBtdXRhdGUocG9zX251bT1tYXB2YWx1ZXMoCiAgICAgIHBvcywgCiAgICAgIHBhc3RlKCdwb3MnLCBjKDEsIDMsIDUsIDcpLCBzZXA9JycpLCAKICAgICAgYygxLCAzLCA1LCA3KSo0NSkpCiAgIyBhZGQgZmFjdG9yIGZvciBwb3NfbnVtCiAgZGZfJHBvc19udW0gPC0gZmFjdG9yKGRmXyRwb3NfbnVtLCBsZXZlbHM9YygxLCAzLCA1LCA3KSo0NSkKICByZXR1cm4oZGZfKQp9CgpkZl9wc2VwX3NlczEgPC0gbWFrZV9wc2VwX2RmKHBzZXBfc2VzMSkKZGZfcHNlcF9zZXMxJHNlc3Npb24gPC0gJzEnCmRmX3BzZXBfc2VzMiA8LSBtYWtlX3BzZXBfZGYocHNlcF9zZXMyKQpkZl9wc2VwX3NlczIkc2Vzc2lvbiA8LSAnMicKCnBzZXAgPC0gcmJpbmQoZGZfcHNlcF9zZXMxLCBkZl9wc2VwX3NlczIpCmBgYAoKYGBge3J9CnBzZXAgJT4lCiAgZ2dwbG90KGFlcyhwb3NfbnVtLCBwc2UqMTAwICsgNTAsIGNvbG9yPXNlc3Npb24sIGdyb3VwPXNlc3Npb24pKSArCiAgZ2VvbV9obGluZSh5aW50ZXJjZXB0PTUwLCBsaW5ldHlwZT0nZGFzaGVkJywgYWxwaGE9MC44KSArCiAgZ2VvbV9wb2ludCgpICsKICBnZW9tX2xpbmUoKSArCiAgbGFicyh4PSdBbmd1bGFyIGxvY2F0aW9uJywgeT0nUFNFIChwb3B1bGF0aW9uKScsIGNvbG9yPSdTZXNzaW9uJykgKwogICNnZ3RpdGxlKCdQb3B1bGF0aW9uLWxldmVsIFBTRScpICsKICBzY2FsZV9jb2xvcl9icmV3ZXIocGFsZXR0ZT0nU2V0MScpICsKICBmYWNldF9ncmlkKH5tb3JwaF90eXBlKSArCiAgc2NhbGVfeV9jb250aW51b3VzKGJyZWFrcz1jKDMwLCA1MCwgNzApKSArCiAgdGhlbWVfUHVibGljYXRpb24oKQogIApgYGAKIyMgU3RhYmlsaXR5IG9mIHBvcHVsYXRpb24gbGV2ZWwgZXN0aW1hdGVzIGFjcm9zcyBzZXNzaW9ucwpMZXQncyBjb21wdXRlIGEgY29ycmVsYXRpb24gYWNyb3NzIHNlc3Npb25zCgpgYGB7ciwgZmlnLmhlaWdodD01LCBmaWcud2lkdGg9NX0KcHNlcF93aWRlIDwtIApwc2VwICU+JSAKICBzcHJlYWQoc2Vzc2lvbiwgcHNlKSAlPiUgCiAgbXV0YXRlKHNlc3Npb24xPS4kJzEnLCBzZXNzaW9uMj0uJCcyJykKCmdncGxvdChwc2VwX3dpZGUsIGFlcyhzZXNzaW9uMSoxMDAgKyA1MCwgc2Vzc2lvbjIqMTAwICsgNTAsIHNoYXBlPW1vcnBoX3R5cGUsIGdyb3VwPTEpKSArIAogIGdlb21fc21vb3RoKG1ldGhvZD0nbG0nLCBjb2xvcj0nZGFya2dyYXknLCBzZT1GKSArCiAgZ2VvbV9wb2ludCgpICsKICBsYWJzKHg9J0ZpcnN0IG1lYXN1cmVtZW50IChQU0UpJywgeT0nU2Vjb25kIG1lYXN1cmVtZW50IChQU0UpJywgc2hhcGU9J01vcnBoJykgKwogIHRoZW1lX1B1YmxpY2F0aW9uKCkgKyAKICBjb29yZF9lcXVhbCgpICsKICAjZ3VpZGVzKHNoYXBlPUYpCiAgdGhlbWUobGVnZW5kLnBvc2l0aW9uPWMoMC45NSwgMC4xNSksCiAgICAgICAgbGVnZW5kLmRpcmVjdGlvbj0ndmVydGljYWwnLAogICAgICAgIGxlZ2VuZC5rZXkuc2l6ZT11bml0KC44LCAncGljYXMnKSwKICAgICAgICBsZWdlbmQudGl0bGU9ZWxlbWVudF90ZXh0KHNpemU9MTApKQogICNjb29yZF9lcXVhbCh4bGltPWMoMjAsIDk1KSwgeWxpbT1jKDIwLCA5NSkpICsKICAjc2NhbGVfeF9jb250aW51b3VzKGJyZWFrcz1zZXEoMjAsIDkwLCAxMCkpICsKICAjc2NhbGVfeV9jb250aW51b3VzKGJyZWFrcz1zZXEoMjAsIDkwLCAxMCkpCgpnZ3NhdmUoJy4uL2ltZy9wc2VfcG9wX3NjYXR0ZXIucG5nJywgd2lkdGg9NSwgaGVpZ2h0PTUpCmBgYApBbmQgdGhlc2UgYXJlIHRoZSBjb3JyZWxhdGlvbnMKYGBge3J9CnNldC5zZWVkKDM0MzIpCmJvb3RFUyhwc2VwX3dpZGVbYygnc2Vzc2lvbjEnLCAnc2Vzc2lvbjInKV0sIFI9MTAwMDApCmBgYApgYGB7cn0KY29yLnRlc3QocHNlcF93aWRlJHNlc3Npb24xLCBwc2VwX3dpZGUkc2Vzc2lvbjIpCmBgYApUaGV5IGFyZSB2ZXJ5IGNvbnNpc3RlbnQgYWNyb3NzIHNlc3Npb25zLiAKCiMjIFN0YWJpbGl0eSBvZiBzdWJqZWN0LWxldmVsIGVzdGltYXRlcyBhY3Jvc3Mgc2Vzc2lvbnMKYGBge3J9CiMgYWRkIG1vcnBoIHR5cGUKZm9yIChtb3JwaCBpbiBtb3JwaHMpIHsKICBkcHNlX3NlczFbW21vcnBoXV0kc3ViamVjdCA8LSByb3cubmFtZXMoZHBzZV9zZXMxW1ttb3JwaF1dKQogIGRwc2Vfc2VzMltbbW9ycGhdXSRzdWJqZWN0IDwtIHJvdy5uYW1lcyhkcHNlX3NlczJbW21vcnBoXV0pCn0KIyBnZXQgZHBzZSBpbiBsb25nIGZvcm1hdCBmb3IgcGxvdHRpbmcKZHBzZV9zZXMxX2xvbmcgPC0KICBsZHBseSgKICAgIGxhcHBseShkcHNlX3NlczEsIGZ1bmN0aW9uKHgpIGdhdGhlcih4LCBwb3MsIHBzZSwgLXN1YmplY3QpKSwKICAgIGRhdGEuZnJhbWUpICU+JQogIG11dGF0ZShtb3JwaF90eXBlPS5pZCwgc2Vzc2lvbj0nMScpCmRwc2Vfc2VzMl9sb25nIDwtCiAgbGRwbHkoCiAgICBsYXBwbHkoZHBzZV9zZXMyLCBmdW5jdGlvbih4KSBnYXRoZXIoeCwgcG9zLCBwc2UsIC1zdWJqZWN0KSksCiAgICBkYXRhLmZyYW1lKSAlPiUKICBtdXRhdGUobW9ycGhfdHlwZT0uaWQsIHNlc3Npb249JzInKQoKZHBzZSA8LSAKICByYmluZChkcHNlX3NlczFfbG9uZywgZHBzZV9zZXMyX2xvbmcpICU+JQogIG11dGF0ZShwb3NfbnVtPW1hcHZhbHVlcyhwb3MsIAogICAgICAgICAgICAgICAgICAgICAgICAgICBwYXN0ZSgncG9zJywgYygxLCAzLCA1LCA3KSwgc2VwPScnKSwgCiAgICAgICAgICAgICAgICAgICAgICAgICAgIGMoMSwgMywgNSwgNykqNDUpKQpkcHNlJHBvc19udW0gPC0gZmFjdG9yKGRwc2UkcG9zX251bSwgbGV2ZWxzPWMoMSwgMywgNSwgNykqNDUpCmBgYAoKYGBge3IsIGVjaG89RkFMU0UsIGZpZy5oZWlnaHQ9MTIsIGZpZy53aWR0aD04fQpkcHNlICU+JQogIGdncGxvdChhZXMocG9zX251bSwgcHNlKjEwMCwgY29sb3I9c2Vzc2lvbiwgZ3JvdXA9c2Vzc2lvbikpICsKICBnZW9tX3BvaW50KCkgKwogIGdlb21fbGluZSgpICsKICBsYWJzKHg9J0FuZ3VsYXIgbG9jYXRpb24nLCB5PVRlWCgnJFxcRGVsdGEkUFNFJyksIGNvbG9yPSdTZXNzaW9uJykgKwogICNnZ3RpdGxlKFRlWCgnU3ViamVjdC1sZXZlbCAkXFxEZWx0YSRQU0UnKSkgKwogIHNjYWxlX2NvbG9yX2JyZXdlcihwYWxldHRlPSdTZXQxJykgKwogIGZhY2V0X2dyaWQoc3ViamVjdH5tb3JwaF90eXBlKSArIHRoZW1lX1B1YmxpY2F0aW9uKCkKCmdnc2F2ZSgnLi4vaW1nL2Rwc2VfZ21tLnBuZycsIGhlaWdodD0xMSwgd2lkdGg9OCkKYGBgCkF2ZXJhZ2UgdGhlIGVzdGltYXRlcyBhY3Jvc3Mgc2Vzc2lvbnMgYW5kIHBsb3QgdGhlbQpgYGB7ciwgZmlnLmhlaWdodD0zLCBmaWcud2lkdGg9OH0KZHBzZSAlPiUgCiAgZ3JvdXBfYnkoc3ViamVjdCwgbW9ycGhfdHlwZSwgcG9zX251bSkgJT4lIAogIHN1bW1hcmlzZShwc2U9bWVhbihwc2UpKSAlPiUKICBnZ3Bsb3QoYWVzKHBvc19udW0sIHBzZSoxMDAsIGNvbG9yPXN1YmplY3QsIGdyb3VwPXN1YmplY3QpKSArCiAgZ2VvbV9wb2ludChhbHBoYT0wLjgpICsKICBnZW9tX2xpbmUoYWxwaGE9MC44KSArCiAgZmFjZXRfd3JhcCh+bW9ycGhfdHlwZSwgbnJvdz0xKSArCiAgdGhlbWVfUHVibGljYXRpb24oKSArCiAgbGFicyh4PSdBbmd1bGFyIGxvY2F0aW9uJywgeT1UZVgoJyRcXERlbHRhJFBTRScpLCBjb2xvcj0nU3ViamVjdCcpICsKICBzY2FsZV95X2NvbnRpbnVvdXMobGltaXRzPWMoLTYwLCA1MCkpICsKICB0aGVtZSgjbGVnZW5kLnBvc2l0aW9uPWMoMC45NSwgMC4xNSksCiAgICAgICAgbGVnZW5kLmRpcmVjdGlvbj0ndmVydGljYWwnLAogICAgICAgIGxlZ2VuZC5wb3NpdGlvbj0ncmlnaHQnLAogICAgICAgIGxlZ2VuZC5rZXkuc2l6ZT11bml0KC44LCAncGljYXMnKSwKICAgICAgICBsZWdlbmQudGl0bGU9ZWxlbWVudF90ZXh0KHNpemU9MTApKQpnZ3NhdmUoJy4uL2ltZy9kcHNlX3N1YmplY3RzLnBuZycsIHdpZHRoPTgsIGhlaWdodD0zKQpgYGAKTGV0J3MgYWxzbyBwbG90IGFuIGV4YW1wbGUgZml0IGZvciBzdWJqZWN0IHMxMApgYGB7cn0KIyBnZXQgdGhlIGRhdGEgdG8gcGxvdCBpbmRpdmlkdWFsIHBvaW50cwpkZl9zMTBfcGxvdF9hYiA8LQogIGRmICU+JQogIGZpbHRlcihzdWJqZWN0ID09ICdzMTAnLCBzZXNzaW9uID09ICcxJykgJT4lCiAgZmlsdGVyKG1vcnBoX3R5cGUgPT0gJ2FiJywgcG9zX251bSAlaW4lIGMoJzIyNScsICczMTUnKSkKZGZfczEwX3Bsb3RfYWMgPC0KICBkZiAlPiUKICBmaWx0ZXIoc3ViamVjdCA9PSAnczEwJywgc2Vzc2lvbiA9PSAnMScpICU+JQogIGZpbHRlcihtb3JwaF90eXBlID09ICdhYycsIHBvc19udW0gJWluJSBjKCcxMzUnLCAnMzE1JykpCmRmX3MxMF9wbG90X2JjIDwtCiAgZGYgJT4lCiAgZmlsdGVyKHN1YmplY3QgPT0gJ3MxMCcsIHNlc3Npb24gPT0gJzEnKSAlPiUKICBmaWx0ZXIobW9ycGhfdHlwZSA9PSAnYmMnLCBwb3NfbnVtICVpbiUgYygnMTM1JywgJzMxNScpKQoKZGZfczEwX3Bsb3QgPC0KICByYmluZChkZl9zMTBfcGxvdF9hYiwgZGZfczEwX3Bsb3RfYWMsIGRmX3MxMF9wbG90X2JjKQoKIyBub3cgZ2V0IHRoZSBwc2UgdmFsdWVzIHRvIHBsb3QKcHNlcDFfcGxvdCA8LQpwc2VwICU+JSAKICBmaWx0ZXIoc2Vzc2lvbiA9PSAnMScpICU+JQogIGFycmFuZ2UocG9zLCBtb3JwaF90eXBlKQpkcHNlMV9wbG90IDwtCmRwc2UgJT4lIAogIGZpbHRlcihzdWJqZWN0ID09ICdzMTAnLCBzZXNzaW9uID09ICcxJykgJT4lIAogIGFycmFuZ2UocG9zLCBtb3JwaF90eXBlKSAlPiUKICBtdXRhdGUoZHBzZT1wc2UpICU+JQogIHNlbGVjdChtb3JwaF90eXBlLCBwb3MsIHBvc19udW0sIGRwc2UpCgpwc2VfczEwX3Bsb3QgPC0gbWVyZ2UocHNlcDFfcGxvdCwgZHBzZTFfcGxvdCkKcHNlX3MxMF9wbG90IDwtCnBzZV9zMTBfcGxvdCAlPiUKICBtdXRhdGUodHBzZT0ocHNlK2Rwc2UpKjEwMCs1MCkKYGBgCmBgYHtyLCBmaWcuaGVpZ2h0PTYsIGZpZy53aWR0aD04fQpwcmVkaWN0X3N1YmogJT4lCiAgZmlsdGVyKHN1YmplY3QgPT0gJ3MxMCcsIHNlc3Npb24gPT0gJzEnKSAlPiUKICBnZ3Bsb3QoYWVzKG1vcnBoX3Jlc2MqMTAwICsgNTAsIHByZWQqMTAwLCBjb2xvcj1hcy5mYWN0b3IocG9zX251bSkpKSArCiAgZ2VvbV9zZWdtZW50KGFlcyh4PXRwc2UsIHhlbmQ9dHBzZSwgeT0tMTAsIHllbmQ9NTApLCBhbHBoYT0wLjgsIGxpbmV0eXBlPSdkYXNoZWQnLCBkYXRhPXBzZV9zMTBfcGxvdCkgKwogIGdlb21fbGluZSgpICsKICBmYWNldF93cmFwKH5tb3JwaF90eXBlLCBuY29sPTEpICsKICBjb29yZF9lcXVhbCh5bGltPWMoLTAuNCwgMTAxKSkgKwogIHRoZW1lX1B1YmxpY2F0aW9uKCkgKwogIHRoZW1lKCNsZWdlbmQucG9zaXRpb249YygwLjk1LCAwLjE1KSwKICAgICAgICBsZWdlbmQuZGlyZWN0aW9uPSd2ZXJ0aWNhbCcsCiAgICAgICAgbGVnZW5kLnBvc2l0aW9uPSdyaWdodCcsCiAgICAgICAgbGVnZW5kLmtleS5zaXplPXVuaXQoLjgsICdwaWNhcycpLAogICAgICAgIGxlZ2VuZC50aXRsZT1lbGVtZW50X3RleHQoc2l6ZT0xMCkpICsKICBsYWJzKHg9J1BlcmNlbnRhZ2UgbW9ycGhpbmcnLCB5PSdQZXJjZW50YWdlIHJlc3BvbnNlcyB0b1xuc2Vjb25kIGlkZW50aXR5JywgY29sb3I9J0FuZ3VsYXJcbmxvY2F0aW9uJykgKwogIHN0YXRfc3VtbWFyeShhZXMobW9ycGgsIHJlc3BvbnNlX2JpbiksIGZ1bi55PWZ1bmN0aW9uKHgpIHN1bSh4KS9sZW5ndGgoeCkqMTAwLCBnZW9tPSdwb2ludCcsIAogICAgICAgICAgICAgICBkYXRhPWRmX3MxMF9wbG90LCBzaXplPTAuOSwgc2hvdy5sZWdlbmQ9RikKZ2dzYXZlKCcuLi9pbWcvZXhhbXBsZWZpdF9zMTAucG5nJywgd2lkdGg9OCwgaGVpZ2h0PTYpCmBgYAoKQ29ycmVsYXRpb24gb2Ygc3ViamVjdC1sZXZlbCBlc3RpbWF0ZXMgYWNyb3NzIHNlc3Npb25zCmBgYHtyLCBmaWcuaGVpZ2h0PTUsIGZpZy53aWR0aD01fQpkcHNlX3dpZGUgPC0KZHBzZSAlPiUKICBzcHJlYWQoc2Vzc2lvbiwgcHNlKSAlPiUKICBtdXRhdGUoc2Vzc2lvbjE9LiQnMScsIHNlc3Npb24yPS4kJzInKQoKZ2dwbG90KGRwc2Vfd2lkZSwgYWVzKHNlc3Npb24xKjEwMCwgc2Vzc2lvbjIqMTAwLCBjb2xvcj1zdWJqZWN0KSkgKyAKICBnZW9tX3Ntb290aChtZXRob2Q9J2xtJywgY29sb3I9J2RhcmtncmF5Jywgc2U9RikgKwogIGdlb21fcG9pbnQoKSArCiAgbGFicyh4PVRlWCgnRmlyc3QgbWVhc3VyZW1lbnQgKCRcXERlbHRhJFBTRSknKSwgCiAgICAgICB5PVRlWCgnU2Vjb25kIG1lYXN1cmVtZW50ICgkXFxEZWx0YSRQU0UpJyksIAogICAgICAgY29sb3I9J1N1YmplY3QnKSArCiAgdGhlbWVfUHVibGljYXRpb24oKSArCiAgY29vcmRfZXF1YWwoKSArCiAgdGhlbWUobGVnZW5kLnBvc2l0aW9uPWMoMC45NSwgMC4yNSksCiAgICAgICAgbGVnZW5kLmRpcmVjdGlvbj0ndmVydGljYWwnLAogICAgICAgIGxlZ2VuZC5rZXkuc2l6ZT11bml0KC44LCAncGljYXMnKSwKICAgICAgICBsZWdlbmQudGl0bGU9ZWxlbWVudF90ZXh0KHNpemU9MTApKQogICNjb29yZF9lcXVhbCh4bGltPWMoLTYwLCA0NSksIHlsaW09YygtNjAsIDQ1KSkgKwogICNzY2FsZV94X2NvbnRpbnVvdXMoYnJlYWtzPXNlcSgtNjAsIDQwLCAyMCkpICsKICAjc2NhbGVfeV9jb250aW51b3VzKGJyZWFrcz1zZXEoLTYwLCA0MCwgMjApKQoKZ2dzYXZlKCcuLi9pbWcvcHNlX3N1Ympfc2NhdHRlci5wbmcnLCB3aWR0aD01LCBoZWlnaHQ9NSkKYGBgCkFuZCB0aGVzZSBhcmUgdGhlIGNvcnJlbGF0aW9ucy4KYGBge3J9CnNldC5zZWVkKDIzNDQ4KQpib290RVMoZHBzZV93aWRlW2MoJ3Nlc3Npb24xJywgJ3Nlc3Npb24yJyldLCBSPTEwMDAwKQpgYGAKYGBge3J9CmNvci50ZXN0KGRwc2Vfd2lkZSRzZXNzaW9uMSwgZHBzZV93aWRlJHNlc3Npb24yKQpgYGAKIyMgV2l0aGluLSB2cy4gYmV0d2Vlbi1zdWJqZWN0cyBjb3JyZWxhdGlvbgpMZXQncyBjb21wdXRlIHRoZSBjb3JyZWxhdGlvbiBiZXR3ZWVuIHRoZSBmaXJzdCBhbmQgdGhlIHNlY29uZCBzZXNzaW9uCmBgYHtyfQpjb3Jfc2VzMTIgPC0gbGlzdCgpCmZvciAobW9ycGggaW4gbW9ycGhzKSB7CiAgdGhpc19jb3IgPC0gY29yKHQoZHBzZV9zZXMxW1ttb3JwaF1dWywgMTo0XSksIHQoZHBzZV9zZXMyW1ttb3JwaF1dWywgMTo0XSkpCiAgIyBtYWtlIGl0IHN5bW1ldHJpYwogIHRoaXNfY29yIDwtICh0aGlzX2NvciArIHQodGhpc19jb3IpKS8yLgogIGNvcl9zZXMxMltbbW9ycGhdXSA9IHRoaXNfY29yCn0KCiMgbWFrZSBhIGRhdGFmcmFtZSBpbiBsb25nIGZvcm1hdAptYWtlX2Nvcl9sb25nIDwtIGZ1bmN0aW9uKGNvcikgewogIHdpdGhpbiA8LSBkaWFnKGNvcikKICBiZXR3ZWVuIDwtIGNvcltsb3dlci50cmkoY29yKV0KICAKICBkZl93aXRoaW5fYmV0d2VlbiA8LSBkYXRhLmZyYW1lKGNvcnI9Yyh3aXRoaW4sIGJldHdlZW4pLAogICAgICAgICAgICAgICAgICAgICAgICAgICAgICAgICAgdHlwZT1jKHJlcCgnd2l0aGluJywgbGVuZ3RoKHdpdGhpbikpLAogICAgICAgICAgICAgICAgICAgICAgICAgICAgICAgICAgICAgICAgIHJlcCgnYmV0d2VlbicsIGxlbmd0aChiZXR3ZWVuKSkpCiAgICAgICAgICAgICAgICAgICAgICAgICAgICAgICAgICApCiAgcmV0dXJuKGRmX3dpdGhpbl9iZXR3ZWVuKQp9Cgpjb3Jfc2VzMTJfbG9uZyA8LSBzYXBwbHkoY29yX3NlczEyLCBtYWtlX2Nvcl9sb25nLCBzaW1wbGlmeT1GLCBVU0UuTkFNRVM9VCkKIyBhZGQgZXZlcnl0aGluZyB0b2dldGhlciBmb3IgcGxvdHRpbmcKY29yX3NlczEyX2xvbmdfcGxvdCA8LSBsZHBseShjb3Jfc2VzMTJfbG9uZywgZGF0YS5mcmFtZSkKYGBgCgpXZSBjYW4gY2hlY2sgd2hldGhlciB0aGUgZXN0aW1hdGVzIGFyZSBjb25zaXN0ZW50IGFjcm9zcyBzZXNzaW9ucywgYW5kIGFsc28gCnN1YmplY3Qtc3BlY2lmaWMsIGJ5IGNvbXBhcmluZyB0aGUgd2l0aGluLXN1YmplY3QgY29ycmVsYXRpb25zIHdpdGggdGhlIGJldHdlZW4tc3ViamVjdApjb3JyZWxhdGlvbnMuIFdlIHdpbGwgY29tcHV0ZSB0aGUgYm9vdHN0cmFwcGVkIGRpZmZlcmVuY2UgJFx0ZXh0e1dpdGhpbn0gLSBcdGV4dHtCZXR3ZWVufSQuCmBgYHtyfQpyZXF1aXJlKGJvb3RFUykKcmVxdWlyZShicm9vbSkKYm9vdHN0cmFwX3dpdGhpbmJldHdlZW4gPC0gZnVuY3Rpb24oY29ycl9kZikgewogIGIgPC0gYm9vdEVTKGNvcnJfZGYsIAogICAgICAgICAgICAgIGRhdGEuY29sPSdjb3JyJywgZ3JvdXAuY29sPSd0eXBlJywgCiAgICAgICAgICAgICAgY29udHJhc3Q9Yyh3aXRoaW49MSwgYmV0d2Vlbj0tMSksIFI9MTAwMDApICAKICByZXR1cm4oYikKfQoKc2V0LnNlZWQoMTI0KQpib290X2NpcyA8LSBzYXBwbHkoY29yX3NlczEyX2xvbmcsCiAgICAgICAgICAgICAgICAgICBib290c3RyYXBfd2l0aGluYmV0d2VlbiwKICAgICAgICAgICAgICAgICAgIHNpbXBsaWZ5PUYsCiAgICAgICAgICAgICAgICAgICBVU0UuTkFNRVM9VCkKCmV4dHJhY3RfY2lzIDwtIGZ1bmN0aW9uKGJvb3Rlc19vdXQpIHsKICB0MCA8LSBib290ZXNfb3V0JHQwCiAgYm91bmRzIDwtIGJvb3Rlc19vdXQkYm91bmRzCiAgZGYgPC0gZGF0YS5mcmFtZSh0MD10MCwgbGNpPWJvdW5kc1sxXSwgcmNpPWJvdW5kc1syXSkKICByZXR1cm4oZGYpCn0KCmV4dHJhY3RfZGlzdHJpYnV0aW9uIDwtIGZ1bmN0aW9uKGJvb3Rlc19vdXQpIHsKICBkZiA8LSBkYXRhLmZyYW1lKHQ9Ym9vdGVzX291dCR0KQogIHJldHVybihkZikKfQpib290X2Npc19kZiA8LSBsZHBseShib290X2NpcywgZXh0cmFjdF9jaXMpCmJvb3RfZGlzdF9kZiA8LSBsZHBseShib290X2NpcywgZXh0cmFjdF9kaXN0cmlidXRpb24pCmBgYAoKQW5kIGZpbmFsbHkgcGxvdCB0aGVtCmBgYHtyLCBmaWcuaGVpZ2h0PTQsIGZpZy53aWR0aD02fQpnZ3Bsb3QoZGF0YT1ib290X2Rpc3RfZGYsIGFlcyguaWQsIHQpKSArCiAgZ2VvbV92aW9saW4oYWRqdXN0PTIpICsKICBnZW9tX2Vycm9yYmFyKGRhdGE9Ym9vdF9jaXNfZGYsIGFlcyh5bWluPWxjaSwgeW1heD1yY2ksIHk9dDApLCB3aWR0aD0wLjAxKSArIAogIGdlb21fcG9pbnQoZGF0YT1ib290X2Npc19kZiwgYWVzKHk9dDApKSArCiAgZ2VvbV9obGluZSh5aW50ZXJjZXB0PTAsIGxpbmV0eXBlPSdkYXNoZWQnKSArCiAgbGFicyh4PSdNb3JwaCcsIHk9J1dpdGhpbiAtIEJldHdlZW4gc3ViamVjdCBjb3JyZWxhdGlvbnMnKSArCiAgdGhlbWVfUHVibGljYXRpb24oKSArCiAgY29vcmRfZmxpcCgpICsgCiAgdGhlbWUoYXNwZWN0LnJhdGlvPTMvNCkgCmBgYApBbmQgdGhlc2UgYXJlIHRoZSB2YWx1ZXMKYGBge3J9CmJvb3RfY2lzX2RmICU+JSBhcnJhbmdlKC5pZCkKYGBgCkxldCdzIGFsc28gZG8gaXQgc2VwYXJhdGVseSBmb3Igd2l0aGluIGFuZCBiZXR3ZWVuCmBgYHtyfQpib290c3RyYXAgPC0gZnVuY3Rpb24oY29ycl9kZikgewogIGIgPC0gYm9vdEVTKGNvcnJfZGYsIAogICAgICAgICAgICAgIGRhdGEuY29sPSdjb3JyJywgUj0xMDAwMCkgIAogIHJldHVybihiKQp9Cgpjb3Jfc2VzMTJfbG9uZ193aXRoaW4gPC0gc2FwcGx5KGNvcl9zZXMxMl9sb25nLCAKICAgICAgICAgICAgICAgICAgICAgICAgICAgICAgICBmdW5jdGlvbih4KSBmaWx0ZXIoeCwgdHlwZT09J3dpdGhpbicpLAogICAgICAgICAgICAgICAgICAgICAgICAgICAgICAgIHNpbXBsaWZ5PUYsCiAgICAgICAgICAgICAgICAgICAgICAgICAgICAgICAgVVNFLk5BTUVTPVQpCmNvcl9zZXMxMl9sb25nX2JldHdlZW4gPC0gc2FwcGx5KGNvcl9zZXMxMl9sb25nLCAKICAgICAgICAgICAgICAgICAgICAgICAgICAgICAgICBmdW5jdGlvbih4KSBmaWx0ZXIoeCwgdHlwZT09J2JldHdlZW4nKSwKICAgICAgICAgICAgICAgICAgICAgICAgICAgICAgICBzaW1wbGlmeT1GLAogICAgICAgICAgICAgICAgICAgICAgICAgICAgICAgIFVTRS5OQU1FUz1UKQoKc2V0LnNlZWQoMzI0MykKYnNfd2l0aGluIDwtIGxkcGx5KAogIGxhcHBseShjb3Jfc2VzMTJfbG9uZ193aXRoaW4sIGJvb3RzdHJhcCksCiAgZXh0cmFjdF9jaXMpCnNldC5zZWVkKDIzNDIzKQpic19iZXR3ZWVuIDwtIGxkcGx5KAogIGxhcHBseShjb3Jfc2VzMTJfbG9uZ19iZXR3ZWVuLCBib290c3RyYXApLAogIGV4dHJhY3RfY2lzKQpgYGAKCmBgYHtyfQpic193aXRoaW4gJT4lIGFycmFuZ2UoLmlkKQpgYGAKYGBge3J9CmJzX2JldHdlZW4gJT4lIGFycmFuZ2UoLmlkKQpgYGAKCiMjIENvcnJlbGF0aW9uIHdpdGggZmFtaWxpYXJpdHkgcmF0aW5ncwoKYGBge3J9CmRmX3F1ZXN0IDwtIHJlYWRfY3N2KCcuLi9kYXRhL3F1ZXN0aW9ubmFpcmUuY3N2JywKICAgICAgICAgICAgICAgICAgICAgY29sX3R5cGVzPSdjY2RkZGRkZGRkZGRkZGQnKQpgYGAKCldlIGFyZSBnb2luZyB0byBjcmVhdGUgYSBjb21wb3NpdGUgc2NvcmUgYnkgc2ltcGx5IGF2ZXJhZ2luZyB0aGUgcXVlc3Rpb25zCnJlbGF0ZWQgdG8gY2xvc2VuZXNzLgpgYGB7cn0KIyBhZGQgY29tcG9zaXRlIHNjb3JlCmRmX3F1ZXN0IDwtCmRmX3F1ZXN0ICU+JQogICMgZXZlcnlib2R5IGdvdCB0aGUgbmFtZSByaWdodAogIHNlbGVjdCgtbmFtZSkgJT4lCiAgcm93d2lzZSgpICU+JQogICMgTGV0J3MgbWFrZSBhIGNvbXBvc2l0ZSBzY29yZSBieSBhdmVyYWdpbmcgaW9zLCB3ZXNjYWxlLCBzY2kxLCBzY2kyCiAgbXV0YXRlKGNvbXBzY29yZT1tZWFuKGMoaW9zLCB3ZXNjYWxlLCBzY2kxLCBzY2kyKSkpCmBgYApMZXQncyBzdGFydCBzaW1wbHkgYnkgbG9va2luZyBhdCB0aGUgYXZlcmFnZSB2YWx1ZXMgZm9yIGVhY2ggaWQKYGBge3J9CmRmX3F1ZXN0X2xvbmcgPC0KZGZfcXVlc3QgJT4lCiAgZ2F0aGVyKHNjYWxlLCB2YWx1ZSwgLXN1YmplY3QsIC1zdGltKQpgYGAKCmBgYHtyfQpkZl9xdWVzdF9sb25nICU+JQogIGdncGxvdChhZXMoc2NhbGUsIHZhbHVlLCBjb2xvcj1zdGltKSkgKwogIHN0YXRfc3VtbWFyeShmdW4uZGF0YT1tZWFuX2NsX2Jvb3QsIGdlb209J2Vycm9yYmFyJywgd2lkdGg9MC4xLCBwb3NpdGlvbj1wb3NpdGlvbl9kb2RnZSh3PTAuMykpICsKICBzdGF0X3N1bW1hcnkoZnVuLnk9bWVhbiwgZ2VvbT0ncG9pbnQnLCBwb3NpdGlvbj1wb3NpdGlvbl9kb2RnZSh3PTAuMykpICsKICB0aGVtZV9QdWJsaWNhdGlvbigpICsKICB0aGVtZShheGlzLnRleHQueD1lbGVtZW50X3RleHQoYW5nbGU9NDUsIGhqdXN0PTEpKQoKZ2dzYXZlKCcuLi9pbWcvc2NvcmVzLnBuZycsIHdpZHRoPTgsIGhlaWdodD02KQpgYGAKCmBgYHtyfQojIGNvbXB1dGUgYSBzY29yZSBvZiBkZXZpYW5jZSBmcm9tIDAgZm9yIGVhY2ggc3ViamVjdCBhbmQgbW9ycGggdHlwZQojIGJhc2ljYWxseSB0aGUgdmFyaWFuY2UKZHBzZV9kZXZpYW5jZSA8LQpkcHNlICU+JQogIGdyb3VwX2J5KHN1YmplY3QsIG1vcnBoX3R5cGUsIHBvc19udW0pICU+JSAKICBzdW1tYXJpc2UocHNlPW1lYW4ocHNlKSkgJT4lCiAgZ3JvdXBfYnkoc3ViamVjdCwgbW9ycGhfdHlwZSkgJT4lCiAgc3VtbWFyaXNlKHBzZXNjb3JlPXN1bShwc2VeMikpCgojIG5vdyBmb3IgZWFjaCBzdWJqZWN0IGNvbXB1dGUgdGhlIGF2ZXJhZ2Ugc2NvcmUgZm9yIHRoZSBtb3JwaHMKZGZfcXVlc3RfYXZnIDwtCmRmX3F1ZXN0ICU+JQogIHNlbGVjdChzdWJqZWN0LCBzdGltLCBjb21wc2NvcmUpICU+JQogIHNwcmVhZChzdGltLCBjb21wc2NvcmUpICU+JQogIHJvd3dpc2UoKSAlPiUKICBtdXRhdGUoc2NvcmVfYWI9bWVhbihjKGEsIGIpKSwgCiAgICAgICAgIHNjb3JlX2JjPW1lYW4oYyhiLCBjKSksIAogICAgICAgICBzY29yZV9hYz1tZWFuKGMoYSwgYykpKSAlPiUKICBzZWxlY3QoLWEsIC1iLCAtYykgJT4lCiAgZ2F0aGVyKG1vcnBoX3R5cGUsIHNjb3JlLCAtc3ViamVjdCkKCmRmX3F1ZXN0X2F2ZyRtb3JwaF90eXBlIDwtCiAgbWFwdmFsdWVzKGRmX3F1ZXN0X2F2ZyRtb3JwaF90eXBlLAogICAgICAgICAgICBjKCdzY29yZV9hYicsICdzY29yZV9hYycsICdzY29yZV9iYycpLAogICAgICAgICAgICBjKCdhYicsICdhYycsICdiYycpKQpkZl9xdWVzdF9kZXZpYW5jZSA8LQpkZl9xdWVzdF9hdmcgJT4lCiAgYXJyYW5nZShzdWJqZWN0LCBtb3JwaF90eXBlKSAlPiUKICBtZXJnZShkcHNlX2RldmlhbmNlKQpgYGAKCmBgYHtyLCBmaWcuaGVpZ2h0PTUsIGZpZy53aWR0aD01fQpnZ3Bsb3QoYWVzKHNjb3JlLCBwc2VzY29yZSwgY29sb3I9bW9ycGhfdHlwZSksIGRhdGE9ZGZfcXVlc3RfZGV2aWFuY2UpICsKICBnZW9tX3Ntb290aChtZXRob2Q9J2xtJywgYWVzKGdyb3VwPTEpLCBzZT1GLCBjb2xvcj0nZGFya2dyYXknKSArIAogIGdlb21fcG9pbnQoKSArCiAgdGhlbWVfUHVibGljYXRpb24oKSArCiAgY29vcmRfZml4ZWQocmF0aW89MTApICsKICBsYWJzKHg9J0ZhbWlsaWFyaXR5IHNjb3JlJywgeT1UZVgoJyRcXERlbHRhJFBTRSB2YXJpYW5jZScpLCBjb2xvcj0nTW9ycGggdHlwZScpICsgCiAgdGhlbWUobGVnZW5kLnBvc2l0aW9uPWMoMC45NSwgMC42KSwKICAgICAgICBsZWdlbmQuZGlyZWN0aW9uPSd2ZXJ0aWNhbCcsCiAgICAgICAgbGVnZW5kLmtleS5zaXplPXVuaXQoLjgsICdwaWNhcycpLAogICAgICAgIGxlZ2VuZC50aXRsZT1lbGVtZW50X3RleHQoc2l6ZT0xMCkpICsKICBzY2FsZV9jb2xvcl9icmV3ZXIocGFsZXR0ZT0nU2V0MicpCmdnc2F2ZSgnLi4vaW1nL3BzZXF1ZXN0X3NjYXR0ZXIucG5nJywgd2lkdGg9NSwgaGVpZ2h0PTUpCmBgYApgYGB7cn0KY29yLnRlc3QoZGZfcXVlc3RfZGV2aWFuY2Ukc2NvcmUsIGRmX3F1ZXN0X2RldmlhbmNlJHBzZXNjb3JlKQpgYGAKYGBge3J9CnNldC5zZWVkKDMyNCkKYm9vdEVTKGRmX3F1ZXN0X2RldmlhbmNlW2MoJ3Njb3JlJywgJ3BzZXNjb3JlJyldLCBSPTEwMDAwKQpgYGAKCiMjIElzIGl0IHBlcnNvbmFsIGZhbWlsaWFyaXR5IG9yIGlzIGl0IGNvbnRhY3Q/CgpgYGB7cn0KZGZfcXVlc3RfY29udGFjdF9hdmcgPC0KZGZfcXVlc3QgJT4lCiAgc2VsZWN0KHN1YmplY3QsIHN0aW0sIGNvbnRhY3QpICU+JQogIHNwcmVhZChzdGltLCBjb250YWN0KSAlPiUKICByb3d3aXNlKCkgJT4lCiAgbXV0YXRlKGFiPW1lYW4oYyhhLCBiKSksIAogICAgICAgICBiYz1tZWFuKGMoYiwgYykpLCAKICAgICAgICAgYWM9bWVhbihjKGEsIGMpKSkgJT4lCiAgc2VsZWN0KC1hLCAtYiwgLWMpICU+JQogIGdhdGhlcihtb3JwaF90eXBlLCBjb250YWN0LCAtc3ViamVjdCkKCmRmX2NvbnRhY3RfZGV2aWFuY2UgPC0KZGZfcXVlc3RfZGV2aWFuY2UgJT4lCiAgYXJyYW5nZShzdWJqZWN0LCBtb3JwaF90eXBlKSAlPiUKICBtZXJnZShkZl9xdWVzdF9jb250YWN0X2F2ZykKYGBgCgpBcmUgZmFtaWxpYXJpdHkgc2NvcmUgYW5kIGNvbnRhY3QgY29ycmVsYXRlZD8KYGBge3J9CmNvci50ZXN0KGRmX2NvbnRhY3RfZGV2aWFuY2Ukc2NvcmUsIGRmX2NvbnRhY3RfZGV2aWFuY2UkY29udGFjdCkKc2V0LnNlZWQoMzI0KQpib290RVMoZGZfY29udGFjdF9kZXZpYW5jZVtjKCdzY29yZScsICdjb250YWN0JyldLCBSPTEwMDAwKQpgYGAKClJ1biBhIG1vZGVsIHRoYXQgcHJlZGljdHMgdGhlIHZhcmlhbmNlIG9mICRcRGVsdGFcdGV4dHtQU0V9JCB1c2luZyBvbmx5IHRoZQpmYW1pbGlhcml0eSBzY29yZSBhbmQgdGhlbiBhbHNvIHRoZSBjb250YWN0IHNjb3JlLgpgYGB7cn0KbTEgPC0gbG0ocHNlc2NvcmUgfiBzY29yZSwgZGF0YT1kZl9jb250YWN0X2RldmlhbmNlKQptMiA8LSB1cGRhdGUobTEsIC4gfiAuICsgY29udGFjdCkKCnRpZHkobTEpCnRpZHkobTIpCmFub3ZhKG0xLCBtMikKCnN1bW1hcnkobTEpCnN1bW1hcnkobTIpCgpyZXF1aXJlKGhlcGxvdHMpCnJvdW5kKGV0YXNxKG0xKSwgMikKcm91bmQoZXRhc3EobTIpLCAyKQoKIyBjb21wdXRlIGxvZy1saWtlbGlob29kIHJhdGlvIG1hbnVhbGx5Cm0xX2xvZ2xpayA8LSBsb2dMaWsobTEpCm0yX2xvZ2xpayA8LSBsb2dMaWsobTIpCmxyIDwtIGFzLm51bWVyaWMoMiAqIChtMl9sb2dsaWsgLSBtMV9sb2dsaWspKQpwbHIgPC0gcGNoaXNxKGxyLCAxLCBsb3dlci50YWlsPUYpCgpwcmludChwYXN0ZSgiTG9nLWxpa2VsaWhvb2QgcmF0aW86IiwgbHIsICJwdmFsdWU6IiwgcGxyKSkKYGBgCmBgYHtyfQojIGZyb20gaHR0cDovL3d3dy5zdGF0bWV0aG9kcy5uZXQvYWR2c3RhdHMvYm9vdHN0cmFwcGluZy5odG1sCmJzIDwtIGZ1bmN0aW9uKGZvcm11bGEsIGRhdGEsIGluZGljZXMpIHsKICBkIDwtIGRhdGFbaW5kaWNlcyxdICMgYWxsb3dzIGJvb3QgdG8gc2VsZWN0IHNhbXBsZSAKICBmaXQgPC0gbG0oZm9ybXVsYSwgZGF0YT1kKQogIHJldHVybihjb2VmKGZpdCkpIAp9IAoKc2V0LnNlZWQoMjM0KQpyZXN1bHRzIDwtIGJvb3QoZGF0YT1kZl9jb250YWN0X2RldmlhbmNlLCBzdGF0aXN0aWM9YnMsCiAgICAgICAgICAgICAgICBSPTEwMDAwLCBmb3JtdWxhPXBzZXNjb3JlIH4gc2NvcmUgKyBjb250YWN0KQoKdDAgPC0gcmVzdWx0cyR0MApjb25mIDwtIGMoKQpmb3IgKGkgaW4gMTpsZW5ndGgodDApKSB7CiAgYiA8LSBib290LmNpKHJlc3VsdHMsIHR5cGU9J2JjYScsIGluZGV4PWkpCiAgY29uZiA8LSByYmluZChjb25mLCBiJGJjYVs0OjVdKQp9CgpkZl9jaSA8LSBkYXRhLmZyYW1lKHQwPXQwLCBsY2k9Y29uZlssMV0sIHJjaT1jb25mWywyXSkKYGBgCmBgYHtyfQpkZl9jaQpgYGAKTm93IGNvbXB1dGUgcGFydGlhbCBjb3JyZWxhdGlvbnMKYGBge3J9CnBhcnRpYWxvdXRfdmFyaWFibGUgPC0gZnVuY3Rpb24oZGYsIHgsIHksIHopIHsKICBteCA8LSBsbShkZltbeF1dIH4gZGZbW3pdXSkKICBteSA8LSBsbShkZltbeV1dIH4gZGZbW3pdXSkKICBkZl9vdXQgPC0gZGF0YS5mcmFtZShteCRyZXNpZHVhbHMsIG15JHJlc2lkdWFscykKICBuYW1lcyhkZl9vdXQpIDwtIGMoeCwgeSkKICByZXR1cm4oZGZfb3V0KQp9CgojIE1ha2UgcGxvdHMgb2YgcGFydGlhbCBjb3JyZWxhdGlvbiB3aXRoIHJlc2lkdWFscwpwYXJ0aWFsX2NvbnRhY3QgPC0gcGFydGlhbG91dF92YXJpYWJsZShkZl9jb250YWN0X2RldmlhbmNlLCAicHNlc2NvcmUiLCAic2NvcmUiLCAiY29udGFjdCIpCnBhcnRpYWxfc2NvcmUgPC0gcGFydGlhbG91dF92YXJpYWJsZShkZl9jb250YWN0X2RldmlhbmNlLCAicHNlc2NvcmUiLCAiY29udGFjdCIsICJzY29yZSIpCmNvci50ZXN0KHBhcnRpYWxfY29udGFjdCRwc2VzY29yZSwgcGFydGlhbF9jb250YWN0JHNjb3JlKQpjb3IudGVzdChwYXJ0aWFsX3Njb3JlJHBzZXNjb3JlLCBwYXJ0aWFsX3Njb3JlJGNvbnRhY3QpCgpzZXQuc2VlZCgzMjQyKQpib290RVMocGFydGlhbF9jb250YWN0W2MoJ3BzZXNjb3JlJywgJ3Njb3JlJyldLCBSPTEwMDAwKQpib290RVMocGFydGlhbF9zY29yZVtjKCdwc2VzY29yZScsICdjb250YWN0JyldLCBSPTEwMDAwKQpgYGAKYGBge3J9CiMgYWRkIHRoZSBtb3JwaCB0eXBlcyB0byBwYXJ0aWFsX2NvbnRhY3QgYW5kIHBhcnRpYWxfc2NvcmUKcGFydGlhbF9jb250YWN0JG1vcnBoX3R5cGUgPC0gZGZfY29udGFjdF9kZXZpYW5jZSRtb3JwaF90eXBlCnBhcnRpYWxfc2NvcmUkbW9ycGhfdHlwZSA8LSBkZl9jb250YWN0X2RldmlhbmNlJG1vcnBoX3R5cGUKYGBgCgpgYGB7cn0KIyBqdXN0IGEgdGhlbWUgdG8gdW5pZm9ybSBzY2F0dGVycwp0aGVtZV9QdWJsaWNhdGlvbl9zY2F0dGVyIDwtIGZ1bmN0aW9uKGJhc2Vfc2l6ZT0xMikgewogICh0aGVtZV9QdWJsaWNhdGlvbihiYXNlX3NpemU9YmFzZV9zaXplKSArIAogIHRoZW1lKGxlZ2VuZC5wb3NpdGlvbj1jKDAuOTUsIDAuNiksCiAgICAgICAgbGVnZW5kLmRpcmVjdGlvbj0ndmVydGljYWwnLAogICAgICAgIGxlZ2VuZC5rZXkuc2l6ZT11bml0KC44LCAncGljYXMnKSwKICAgICAgICBsZWdlbmQudGl0bGU9ZWxlbWVudF90ZXh0KHNpemU9MTApLAogICAgICAgIGF4aXMudGl0bGU9ZWxlbWVudF90ZXh0KHNpemU9MTApKSkKfQpgYGAKYGBge3IsIGZpZy5oZWlnaHQ9MywgZmlnLndpZHRoPTN9CmdncGxvdChhZXMoc2NvcmUsIHBzZXNjb3JlLCBjb2xvcj1tb3JwaF90eXBlKSwgZGF0YT1kZl9xdWVzdF9kZXZpYW5jZSkgKwogIGdlb21fc21vb3RoKG1ldGhvZD0nbG0nLCBhZXMoZ3JvdXA9MSksIHNlPUYsIGNvbG9yPSdkYXJrZ3JheScpICsgCiAgZ2VvbV9wb2ludCgpICsKICBjb29yZF9maXhlZChyYXRpbz0xMCkgKwogIGxhYnMoeD0nRmFtaWxpYXJpdHkgc2NvcmUnLCB5PVRlWCgnJFxcRGVsdGEkUFNFIHZhcmlhbmNlJyksIGNvbG9yPSdNb3JwaCB0eXBlJykgKyAKICB0aGVtZV9QdWJsaWNhdGlvbl9zY2F0dGVyKCkgKwogIHNjYWxlX2NvbG9yX2JyZXdlcihwYWxldHRlPSdTZXQyJykKZ2dzYXZlKCcuLi9pbWcvcHNlcXVlc3Rfc2NhdHRlcl9wc2VmYW0ucG5nJywgd2lkdGg9MywgaGVpZ2h0PTMpCmBgYApgYGB7ciwgZmlnLmhlaWdodD0zLCBmaWcud2lkdGg9M30KZ2dwbG90KGFlcyhzY29yZSwgcHNlc2NvcmUsIGNvbG9yPW1vcnBoX3R5cGUpLCBkYXRhPXBhcnRpYWxfY29udGFjdCkgKwogIGdlb21fc21vb3RoKG1ldGhvZD0nbG0nLCBhZXMoZ3JvdXA9MSksIHNlPUYsIGNvbG9yPSdkYXJrZ3JheScpICsgCiAgZ2VvbV9wb2ludCgpICsKICBjb29yZF9maXhlZChyYXRpbz0xMCkgKwogIGxhYnMoeD0nRmFtaWxpYXJpdHkgc2NvcmUgfCBDb250YWN0JywgeT1UZVgoJyRcXERlbHRhJFBTRSB2YXJpYW5jZSB8IENvbnRhY3QnKSwgY29sb3I9J01vcnBoIHR5cGUnKSArIAogIHRoZW1lX1B1YmxpY2F0aW9uX3NjYXR0ZXIoKSArCiAgc2NhbGVfY29sb3JfYnJld2VyKHBhbGV0dGU9J1NldDInKQpnZ3NhdmUoJy4uL2ltZy9wc2VxdWVzdF9zY2F0dGVyX3BzZWZhbV9jb250YWN0LnBuZycsIHdpZHRoPTMsIGhlaWdodD0zKQpgYGAKYGBge3IsIGZpZy5oZWlnaHQ9MywgZmlnLndpZHRoPTN9CmdncGxvdChhZXMoY29udGFjdCwgcHNlc2NvcmUsIGNvbG9yPW1vcnBoX3R5cGUpLCBkYXRhPXBhcnRpYWxfc2NvcmUpICsKICBnZW9tX3Ntb290aChtZXRob2Q9J2xtJywgYWVzKGdyb3VwPTEpLCBzZT1GLCBjb2xvcj0nZGFya2dyYXknKSArIAogIGdlb21fcG9pbnQoKSArCiAgY29vcmRfZml4ZWQocmF0aW89MTAsIGV4cGFuZD1UKSArCiAgbGFicyh4PSdDb250YWN0IHNjb3JlIHwgRmFtaWxpYXJpdHknLCB5PVRlWCgnJFxcRGVsdGEkUFNFIHZhcmlhbmNlIHwgRmFtaWxpYXJpdHknKSwgY29sb3I9J01vcnBoIHR5cGUnKSArIAogIHRoZW1lX1B1YmxpY2F0aW9uX3NjYXR0ZXIoKSArCiAgc2NhbGVfY29sb3JfYnJld2VyKHBhbGV0dGU9J1NldDInKQpnZ3NhdmUoJy4uL2ltZy9wc2VxdWVzdF9zY2F0dGVyX3BzZWNvbnRhY3RfZmFtLnBuZycsIHdpZHRoPTMsIGhlaWdodD0zKQpgYGAKCiMgUFNFIGZvciBpbmRpdmlkdWFsIGlkZW50aXRpZXMKCldlIGFyZSBnb2luZyB0byBldmFsdWF0ZSB0aGUgYmlhcyB0b3dhcmRzIHNwZWNpZmljIGlkZW50aXRpZXMsCmluc3RlYWQgb2YgcGFpcnMgb2YgbW9ycGhzLiBUbyBkbyBzbywgd2UnbGwgZml0IGEgbGluZWFyIG1vZGVsIHByZWRpY3RpbmcgdGhlClBTRSwgdGFraW5nIHRoZSBkYXRhIG9mIGVhY2ggcGFpciBvZiBtb3JwaHMgY29udGFpbmluZyB0aGUgc2FtZSBpZGVudGl0eS4gRm9yCmV4YW1wbGUsIHRvIGV2YWx1YXRlIHRoZSBiaWFzIGZvciBpZGVudGl0eSBgYWAsIHdlJ2xsIGNvbnNpZGVyIHRoZSBQU0UKZXN0aW1hdGVzIGZvciBtb3JwaHMgYGFiYCBhbmQgYGFjYCBhY3Jvc3MgdGhlIHR3byBzZXNzaW9ucywgYW5kIGZpdCBhIGxpbmVhcgptb2RlbCB3aXRoIHBhcnRpY2lwYW50cywgYW5ndWxhciBsb2NhdGlvbiwgYW5kIHRoZWlyIGludGVyYWN0aW9uIGFzIHByZWRpY3RvcnMuCgpgYGB7ciBtZXNzYWdlPUZBTFNFLCB3YXJuaW5nPUZBTFNFfQpyZXF1aXJlKHRpZHl2ZXJzZSkKcmVxdWlyZShsbWU0KQpyZXF1aXJlKGNhcikKcmVxdWlyZShicm9vbSkKYGBgCgpGaXJzdCB3ZSBjb21wdXRlIHRoZSBQU0UgZm9yIGVhY2ggcGFydGljaXBhbnQgYnkgYWRkaW5nIFBTRXAgYW5kIERQU0UuCmBgYHtyfQpjb2xzIDwtIGMoJ3BvczEnLCAncG9zMycsICdwb3M1JywgJ3BvczcnKQpwc2Vfc2VzMSA9IGxpc3QoKQpwc2Vfc2VzMiA9IGxpc3QoKQoKIyBjb21wdXRlIHRoZSBQU0UgYnkgYWRkaW5nIFBTRXAgdG8gRFBTRSBmb3IgZWFjaCBzdWJqZWN0CmZvciAobW9ycGggaW4gYygnYWMnLCAnYWInLCAnYmMnKSkgewogIHBzZXAgPC0gcHNlcF9zZXMxW1ttb3JwaF1dCiAgZHBzZSA8LSBkcHNlX3NlczFbW21vcnBoXV0KICBwc2UgPC0gKG1hdHJpeChyZXAocHNlcFtjb2xzXSwgMTApLCBucm93PTEwLCBieXJvdz1UKSArIAogICAgICAgICAgICAgICAgICAgICAgIGRwc2VbY29sc10pKjEwMCArIDUwCiAgcHNlJHN1YmplY3QgPC0gcm93Lm5hbWVzKHBzZSkKICBwc2Vfc2VzMVtbbW9ycGhdXSA8LSBwc2UKICAKICBwc2VwIDwtIHBzZXBfc2VzMltbbW9ycGhdXQogIGRwc2UgPC0gZHBzZV9zZXMyW1ttb3JwaF1dCiAgcHNlIDwtIChtYXRyaXgocmVwKHBzZXBbY29sc10sIDEwKSwgbnJvdz0xMCwgYnlyb3c9VCkgKyAKICAgICAgICAgICAgICAgICAgICAgICBkcHNlW2NvbHNdKSoxMDAgKyA1MAogIHBzZSRzdWJqZWN0IDwtIHJvdy5uYW1lcyhwc2UpCiAgcHNlX3NlczJbW21vcnBoXV0gPC0gcHNlCn0KCiMgbm93IHN0b3JlIGl0IGluIGxvbmcgZm9ybWF0CnBzZV9zZXMxX2xvbmcgPC0KICBsZHBseSgKICAgIGxhcHBseShwc2Vfc2VzMSwgZnVuY3Rpb24oeCkgbXV0YXRlKHgsIHN1YmplY3Q9cm93bmFtZXMoeCkpICU+JSBnYXRoZXIocG9zLCBwc2UsIC1zdWJqZWN0KSksCiAgICBkYXRhLmZyYW1lKSAlPiUKICBtdXRhdGUobW9ycGhfdHlwZT0uaWQsIHNlc3Npb249JzEnKQpwc2Vfc2VzMl9sb25nIDwtCiAgbGRwbHkoCiAgICBsYXBwbHkocHNlX3NlczIsIGZ1bmN0aW9uKHgpIG11dGF0ZSh4LCBzdWJqZWN0PXJvd25hbWVzKHgpKSAlPiUgZ2F0aGVyKHBvcywgcHNlLCAtc3ViamVjdCkpLAogICAgZGF0YS5mcmFtZSkgJT4lCiAgbXV0YXRlKG1vcnBoX3R5cGU9LmlkLCBzZXNzaW9uPScyJykKCnBzZSA8LSAKICByYmluZChwc2Vfc2VzMV9sb25nLCBwc2Vfc2VzMl9sb25nKSAlPiUKICBtdXRhdGUocG9zX251bT1tYXB2YWx1ZXMocG9zLCAKICAgICAgICAgICAgICAgICAgICAgICAgICAgcGFzdGUoJ3BvcycsIGMoMSwgMywgNSwgNyksIHNlcD0nJyksIAogICAgICAgICAgICAgICAgICAgICAgICAgICBjKDEsIDMsIDUsIDcpKjQ1KSkKcHNlJHBvc19udW0gPC0gZmFjdG9yKHBzZSRwb3NfbnVtLCBsZXZlbHM9YygxLCAzLCA1LCA3KSo0NSkKYGBgCgpMZXQncyBzYXZlIGl0IGZvciBsYXRlciB1c2UuCmBgYHtyfQojIHNhdmUgcHNlCnBzZV9mbiA8LSAnLi4vZGVyaXZhdGl2ZXMvcHNlLmNzdicKCmlmICghZmlsZS5leGlzdHMocHNlX2ZuKSkgewogIHBzZV8gPC0gcHNlICU+JQogICAgc2VsZWN0KHN1YmplY3QsIHNlc3Npb24sIG1vcnBoX3R5cGUsIHBvc19udW0sIHBzZSkKICB3cml0ZV9jc3YocHNlXywgcHNlX2ZuKQp9CmBgYAoKCkhlcmUgd2UgbG9hZCB0aGUgcHJlLWVzdGltYXRlZCBkYXRhCmBgYHtyfQpwc2UgPC0gcmVhZF9jc3YoJy4uL2Rlcml2YXRpdmVzL3BzZS5jc3YnLCBjb2xfdHlwZXM9J2NpY2NkJykKcHNlJG1vcnBoX3R5cGUgPC0gYXMuZmFjdG9yKHBzZSRtb3JwaF90eXBlKQpwc2UkcG9zX251bSA8LSBmYWN0b3IocHNlJHBvc19udW0sIAogICAgICAgICAgICAgICAgICAgICAgbGV2ZWxzPWMoJzQ1JywgJzEzNScsICcyMjUnLCAnMzE1JykpCmBgYAoKCldlIHNldCB1cCBhIGZ1bmN0aW9uIHRvIGZpbHRlciB0aGUgZGF0YSBmb3IgZWFjaCBpbmRpdmlkdWFsIGlkZW50aXR5LiBNb3Jlb3ZlciwKd2UgbWFrZSBzdXJlIHRoYXQgdGhlIFBTRSB2YWx1ZSBpbmRpY2F0ZXMgY29uc2lzdGVudGx5IHRoZSBiaWFzIHdpdGggcmVzcGVjdCB0bwp0aGUgc2FtZSBpZGVudGl0eS4KYGBge3J9CmZpbHRlcl9kYXRhc2V0IDwtIGZ1bmN0aW9uKGlkLCBkZikgewogIGlkZW50aXR5Mm1vcnBocyA8LSBsaXN0KGE9YygnYWInLCAnYWMnKSwKICAgICAgICAgICAgICAgICAgICAgICAgICBiPWMoJ2FiJywgJ2JjJyksCiAgICAgICAgICAgICAgICAgICAgICAgICAgYz1jKCdiYycsICdhYycpKQogIGRmXyA8LQogICAgZGYgJT4lCiAgICBmaWx0ZXIobW9ycGhfdHlwZSAlaW4lIGlkZW50aXR5Mm1vcnBoc1tbaWRdXSkgJT4lCiAgICBkcm9wbGV2ZWxzKC4kbW9ycGhfdHlwZSkKICAKICAjIHB1dCB0aGUgUFNFIGluIHRoZSByaWdodCBvcmRlcjogPiA1MCBpbmRpY2F0ZXMgCiAgIyBtb3JlIHRvd2FyZHMgdGhlIGlkZW50aXR5IG9mIGludGVyZXN0CiAgaWYgKGlkID09ICdiJykgewogICAgZGZfIDwtCiAgICBkZl8gJT4lCiAgICAgIG11dGF0ZShwc2U9aWZlbHNlKG1vcnBoX3R5cGUgPT0gJ2FiJywgMTAwIC0gcHNlLCBwc2UpKQogIH0gZWxzZSBpZiAoaWQgPT0gJ2MnKSB7CiAgICBkZl8gPC0KICAgIGRmXyAlPiUKICAgICAgbXV0YXRlKHBzZT0xMDAgLSBwc2UpCiAgfQogIAogIHJldHVybihkZl8pCn0KYGBgCgoKRmluYWxseSB3ZSBjYW4gZml0IHRoZSBtb2RlbHMKYGBge3J9CmlkcyA8LSBjKCdhJywgJ2InLCAnYycpCiMgc3BsaXQgdGhlIGRhdGFzZXRzCnBzZV9pZHMgPC0gbWFwKGlkcywgZmlsdGVyX2RhdGFzZXQsIHBzZSkKCmZpdF9tb2RlbCA8LSBmdW5jdGlvbihkZikgewogIGRmJHN1YmplY3QgPC0gYXMuZmFjdG9yKGRmJHN1YmplY3QpCiAgZGYkcG9zX251bSA8LSBhcy5mYWN0b3IoZGYkcG9zX251bSkKICBjb250cmFzdHMoZGYkc3ViamVjdCkgPC0gY29udHIuc3VtKGxlbmd0aCh1bmlxdWUoZGYkc3ViamVjdCkpKQogIGNvbnRyYXN0cyhkZiRwb3NfbnVtKSA8LSBjb250ci5zdW0obGVuZ3RoKHVuaXF1ZShkZiRwb3NfbnVtKSkpCiAgCiAgcmV0dXJuKGxtKHBzZSB+IHN1YmplY3QgKiBwb3NfbnVtLCBkYXRhPWRmKSkKfQoKbW9kZWxfaWRzIDwtIAogIG1hcChwc2VfaWRzLCBmaXRfbW9kZWwpICU+JQogIHNldF9uYW1lcyhpZHMpCgphbm92YV9tb2RlbCA8LQogIG1vZGVsX2lkcyAlPiUKICBtYXAoQW5vdmEsIHR5cGU9MykKCnRpZHlfYW5vdmEgPC0KICBhbm92YV9tb2RlbCAlPiUKICBtYXBfZGYodGlkeSwgLmlkPSdpZCcpCmBgYAoKQW5kIGhlcmUgYXJlIHRoZSByZXN1bHRzCmBgYHtyfQphbm92YV9tb2RlbApgYGAKV2UgZmluZCBhIHNpZ25pZmljYW50IGludGVyYWN0aW9uIGJldHdlZW4gcGFydGljaXBhbnRzIGFuZCBhbmd1bGFyIGxvY2F0aW9uIGZvcgppZGVudGl0aWVzIGBiYCBhbmQgYGNgLCBidXQgbm90IGZvciBpZGVudGl0eSBgYWAsIHN1Z2dlc3RpbmcgdGhhdCBmb3IgaWRlbnRpdHkKYGFgIHRoZSBiaWFzIGlzIGNvbnNpc3RlbnRseSBtb3JlIGhvbW9nZW5lb3VzIGFjcm9zcyBwYXJ0aWNpcGFudHMuCgojIyBVc2UgdGhlIG1vZGVsIHRvIHByZWRpY3QgdGhlIGJpYXNlcwoKV2UgY2FuIGFsc28gdXNlIHRoZXNlIG1vZGVscyBub3cgdG8gcHJlZGljdCB0aGUgYmlhc2VzIGF0IGVhY2ggYW5ndWxhciBsb2NhdGlvbgpmb3IgZWFjaCBpbmRpdmlkdWFsIHBhcnRpY2lwYW50LgoKYGBge3J9CmRmX3ByZWQgPC0gZXhwYW5kLmdyaWQoCiAgc3ViamVjdD11bmlxdWUocHNlJHN1YmplY3QpLAogIHBvc19udW09dW5pcXVlKHBzZSRwb3NfbnVtKQopCgpkZl9wcmVkIDwtIAptYXBfZGYobW9kZWxfaWRzLCBwcmVkaWN0LCBkZl9wcmVkKSAlPiUKICBnYXRoZXIoaWRlbnRpdHksIHBzZSkgJT4lCiAgY2JpbmQoZGZfcHJlZCwgLikKYGBgCmBgYHtyfQpnZ3Bsb3QoYWVzKHBvc19udW0sIHBzZS01MCwgY29sb3I9c3ViamVjdCwgZ3JvdXA9c3ViamVjdCksIGRhdGE9ZGZfcHJlZCkgKwogIGdlb21fcG9pbnQoKSArCiAgZ2VvbV9saW5lKCkgKwogIGZhY2V0X3dyYXAofmlkZW50aXR5LCBucm93PTEpCmBgYAoKCgoKCgoKCgoKCg==
