# Supplementary material for: Idiosyncratic, Retinotopic Bias in Face Identification Modulated by Familiarity
Source: eNeuro. 2018 Oct 4;5(5):ENEURO.0054-18.2018. doi: 10.1523/ENEURO.0054-18.2018 (PMC6171739; doi:10.1523/ENEURO.0054-18.2018)
Supplement: Extended Data — The archive contains data from both experiments, as well as the analysis scripts. Download Extended Data 1, ZIP file. [file sup_enu-eN-NWR-0054-18-s02.zip › famretino2-3.0.0/exp2/code/analysis_exp2.pdf]

Experiment 2

Contents

Procedure and equations 2

Model fitting 2

Predict psychometric curves . . . 4
Stability of subject-level estimates across sessions . . . 11
Within- vs. between-subjects correlation . . . 17
Correlation with familiarity ratings . . . 19
Is it personal familiarity or is it contact? . . . 22

PSE for individual identities 28

Use the model to predict the biases . . . 31

Start by loading functions and data

```
require(latex2exp)
require(bootES)
# load plyr *BEFORE* dplyr (i.e., tidyverse)
require(plyr)
require(tidyverse)
require(assertthat)
df <- read_csv('../data/data.csv')

# create output img directory if not existant
dir.create('../img', showWarning=F)
```

Preprocess the data, that is do the following

- remove trials where participants responded with a third option; that is, if they were presented with morph *ab* and they responded with *c*, that trial will be removed
- binarize the responses to run the model

```
df <- df %>%
  filter(third_option != 1)

# now we need to add a binarized response to compute the psychometric curves
# but this changes depending on morph_type, so we need to make a function that
# checks the conditions
binarize_response <- function(morph_type, response_identity) {
  if (morph_type == 'ab') {
    return(ifelse(response_identity == 'a', 0, 1))
  } else if (morph_type == 'bc') {
    return(ifelse(response_identity == 'b', 0, 1))
  } else if (morph_type == 'ac') {
    return(ifelse(response_identity == 'a', 0, 1))
  }
}

df <-
df %>%
  rowwise() %>%
```

```
  mutate(response_bin=binarize_response(morph_type, response_identity))

df$pos <- as.factor(df$pos)
df$session <- as.factor(df$session)

# make sure that binarize_response worked, i.e. we should get 1s and 0s for each
# morph_type
check_ <-
df %>%
  group_by(morph_type) %>%
  summarise(check=length(unique(response_bin)))
assert_that(length(check_$check) == 3)
```

```
## [1] TRUE
```

```
assert_that(all(check_$check == c(2, 2, 2)))
```

```
## [1] TRUE
```

Procedure and equations

We are going to fit a linear mixed effect model to the data. We will model the data as follows

$$y^k = \text{logit}\left(g(x)\right) g(x) = \beta_0 x + \sum_{i=1}^{8} \left(\beta_i + z_i^k\right) I_i$$

Where y^k is the response for subject k, x is the (scaled) percentage of morphing, $\beta_i, i = 1...4$ are the fixed-effects for each angular location (0 to 315 in 45 deg steps), and z_i^k are the random-effects (random slopes for location) for each subject, and I_i is an indicator variable, indicating the angular location for each trial.

In this way for each subject we can find the PSE as the point where $y^k = 0.5$, that is the point $\hat{x}$

$$\text{logit}(g(\hat{x})) = 0.5 \iff g(\hat{x}) = 0 \iff \beta_0 \hat{x} + \sum_{i=1}^{4} \left(\beta_i + z_i^k\right) I_i = 0 \iff \hat{x} = -\frac{\sum_{i=1}^{4} \left(\beta_i + z_i^k\right) I_i}{\beta_0}$$

Thus for every angular location i we have that

$$\hat{x}_i = -\frac{\beta_i}{\beta_0} - \frac{z_i^k}{\beta_0} = \text{PSE}_i^p + \Delta\text{PSE}_i^s$$

with PSE_i^p being the population-level PSE at location i, and ΔPSE_i^s being the change at location i for subject s.

We will fit one such model for each of the morph types, and one for each session.

Model fitting

```
require(lme4)

# define some functions
```

```r
extract_morph_session <- function(df, mt, ses) {
  # Extract trials from one particular morph_type and session
  df_ <-
    df %>%
    filter(morph_type == mt, session == ses) %>%
    mutate(morph_resc=(morph - 50)/100)
  return(df_)
}
run_model_session <- function(df) {
  # Run the following logit mixed-effect model for one session
  # response_bin ~ morph_resc + pos - 1 + (pos - 1 | subject)
  m <- glmer(response_bin ~ morph_resc + pos - 1 + (pos - 1 | subject),
          data=df,
          family=binomial(link='logit'),
          control=glmerControl(optimizer='bobyqa', optCtrl=list(maxfun=20000)))
  return(m)
}
```

Run the model separately for every morph

```r
morphs <- unique(df$morph_type)
df_ses1 <- sapply(morphs, function(x) extract_morph_session(df, x, '1'),
                  simplify=F, USE.NAMES=T)
df_ses2 <- sapply(morphs, function(x) extract_morph_session(df, x, '2'),
                  simplify=F, USE.NAMES=T)
# check that we have all the trials
assert_that(sum(sapply(df_ses1, nrow)) + sum(sapply(df_ses2, nrow)) == nrow(df))
```

```
## [1] TRUE
```

```r
# now compute models
model_ses1 <- sapply(df_ses1, run_model_session, simplify=F, USE.NAMES=T)
model_ses2 <- sapply(df_ses2, run_model_session, simplify=F, USE.NAMES=T)
```

The following functions are used to extract both the population PSE^p and the subject ΔPSE^s. Remember that $\text{PSE}^s = \text{PSE}^p + \Delta\text{PSE}^s$.

```r
population_pse <- function(model) {
  # Computes population-level PSE (see equations above)
  # Note: they are on the scale of morph_resc
  morph_beta <- fixef(model)[1]
  pos_betas <- fixef(model)[-1]
  return(-pos_betas/morph_beta)
}

subjects_pse <- function(model) {
  # Computes subject-level PSE (see equations above)
  morph_pop <- fixef(model)[1]
  position_pop <- fixef(model)[-1]
  ranef_model <- ranef(model)$subject
  # this is the denominator
  morph_subj <- ranef_model[, 1] + morph_pop
  # these are the numerators
  position_subj <- ranef_model[, -1]
  # extend position_beta to get the same shape as ranef_pos
  position_pop <- matrix(rep(position_beta, nrow(ranef_pos)),
```

```
                          byrow=T, nrow=nrow(ranef_pos))
  position_subj <- position_subj + position_pop
  # extend morph_subj to get the same shape as position_subj
  morph_subj <- matrix(rep(morph_subj, ncol(position_subj)), ncol=ncol(position_subj))
  return(-position_subj/morph_subj)
}

delta_pse <- function(model) {
  # Computes subject-level PSE (see equations above)
  # Note: they are on the scale of morph_resc
  # IF 'morph_resc' is entered as a random effect, add that for each individual
  # morph
  morph_beta <- fixef(model)[1]
  ranef_model <- ranef(model)$subject
  if ('morph_resc' %in% names(ranef_model)) {
    pse_pop <- population_pse(model)
    pse_subj <- subjects_pse(model)
    pse_pop <- matrix(rep(pse_pop, nrow(pse_subj)), nrow=nrow(pse_subj), byrow=T)
    return(pse_subj - pse_pop)
  } else {
    return(-ranef_model/morph_beta)
  }
}
```

Let's look at the population estimates for the PSEs across the two sessions, as well as the subject-level estimates.

```
psep_ses1 <- sapply(model_ses1, population_pse, simplify=F, USE.NAMES=T)
psep_ses2 <- sapply(model_ses2, population_pse, simplify=F, USE.NAMES=T)
dpse_ses1 <- sapply(model_ses1, delta_pse, simplify=F, USE.NAMES=T)
dpse_ses2 <- sapply(model_ses2, delta_pse, simplify=F, USE.NAMES=T)
```

Predict psychometric curves

Let's plot the population estimates first

```
df_predict <-
  expand.grid(morph_resc=seq(-0.5, 0.5, 0.01), pos=c('1', '3', '5', '7'))

predict_pop_ses1 <- sapply(model_ses1, predict, newdata=df_predict,
                           re.form=NA, type='response', simplify=F, USE.NAMES=T)
predict_pop_ses2 <- sapply(model_ses2, predict, newdata=df_predict,
                           re.form=NA, type='response', simplify=F, USE.NAMES=T)

# add df_predict to each of them
predict_pop_ses1 <- lapply(predict_pop_ses1, function(x) cbind(df_predict, pred=x))
predict_pop_ses2 <- lapply(predict_pop_ses2, function(x) cbind(df_predict, pred=x))

predict_pop_ses1 <- ldply(predict_pop_ses1, data.frame)
predict_pop_ses2 <- ldply(predict_pop_ses2, data.frame)
predict_pop_ses1$session <- '1'
predict_pop_ses2$session <- '2'

predict_pop <- rbind(predict_pop_ses1, predict_pop_ses2)
```

```r
predict_pop <-
  predict_pop %>%
  mutate(morph=morph_resc*100  + 50,
         pos_num=as.numeric(as.character(pos))*45,
         morph_type=.id)
```

```r
# modified from https://rpubs.com/Koundy/71792
theme_Publication <- function(base_size=12) {
      library(ggthemes)
      (theme_foundation(base_size=base_size)
       + theme(plot.title = element_text(face = "bold",
                                         size = rel(1.2), hjust = 0.5),
               text = element_text(),
               panel.background = element_rect(colour = NA),
               plot.background = element_rect(colour = NA),
               panel.border = element_rect(colour = NA),
               axis.title = element_text(size = rel(1)),
               axis.title.y = element_text(angle=90,vjust =2),
               axis.title.x = element_text(vjust = -0.2),
               axis.text = element_text(),
               axis.line = element_line(colour="black"),
               axis.ticks = element_line(),
               panel.grid.major = element_blank(), #element_line(colour="#f0f0f0"),
               panel.grid.minor = element_blank(),
               legend.key = element_rect(colour = NA),
               legend.position = "bottom",
               legend.direction = "horizontal",
               #legend.key.size= unit(0.2, "cm"),
               legend.spacing = unit(0, "cm"),
               legend.title = element_text(),
               plot.margin = unit(c(10,5,5,5),"mm"),
               strip.background = element_rect(colour="#f0f0f0",fill="#f0f0f0"),
               strip.text = element_text(face="bold"),
               strip.text.y = element_text(angle = 0)
          ))

}
```

```r
df <-
  df %>%
  mutate(pos_num=as.numeric(as.character(pos))*45)

predict_pop %>%
  ggplot(aes(morph, pred*100, color=session)) +
  geom_line() +
  geom_hline(yintercept=50, size=0.5, color='darkgray', alpha=1, linetype='dashed') +
  geom_vline(xintercept=50, size=0.5, color='darkgray', alpha=1, linetype='dashed') +
  # add individual data
  stat_summary(data=df,
               aes(morph, response_bin),
               fun.y=function(x) sum(x)/length(x)*100, geom='point') +
  facet_grid(morph_type ~ pos_num) +
  labs(x='Percentage morphing', y='Population prediction\npercent responses to second identity', color=
  scale_color_brewer(palette='Set1') +
```

```r
  theme_Publication() +
  coord_equal()
```

```
## Warning: package 'ggthemes' was built under R version 3.2.5
```

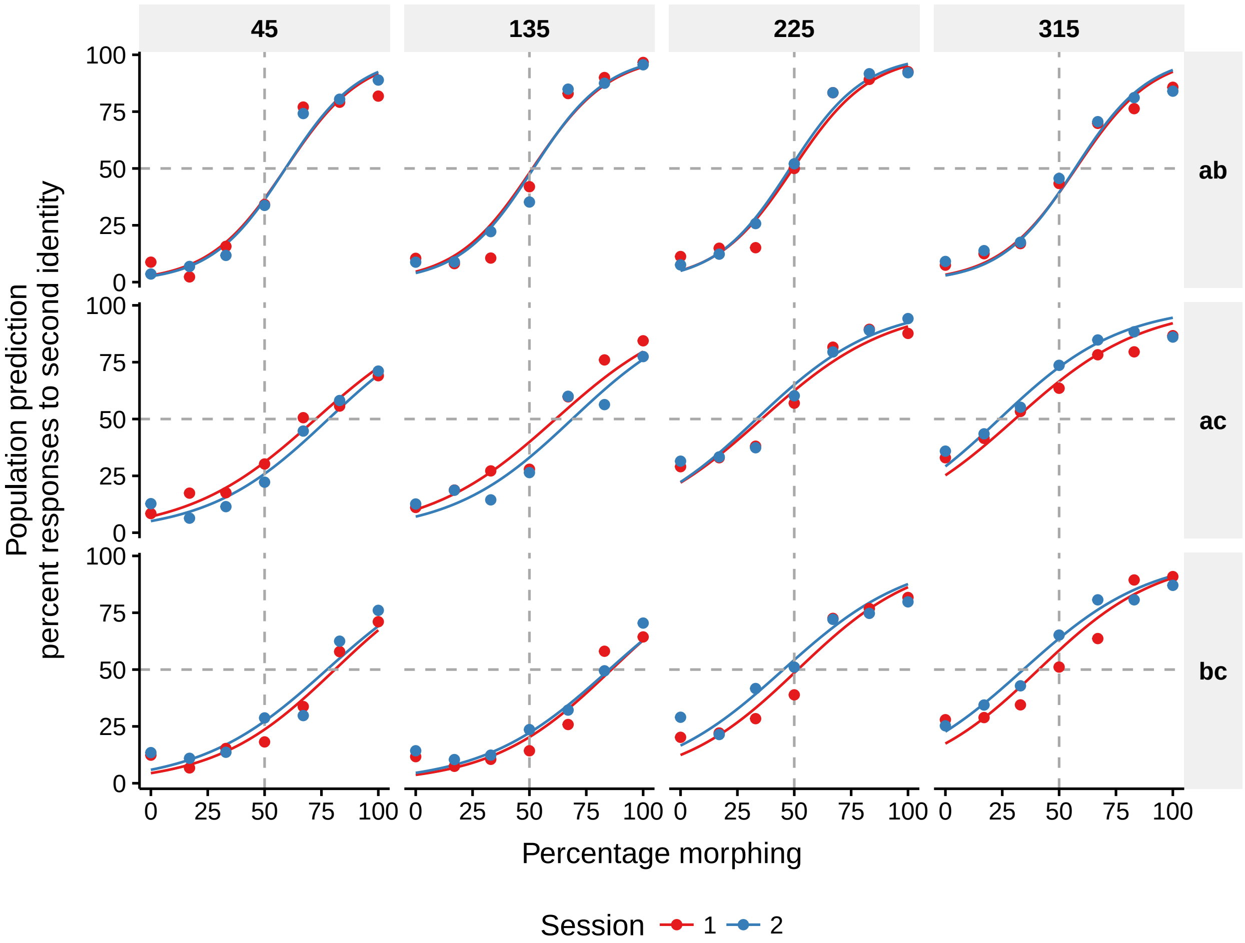


```r
ggsave('../img/pred_pop_gmm.png', width=8, height=6)
```

Now we can predict for each individual subject

```r
df_predict <-
  expand.grid(
    morph_resc=seq(-0.5, 0.5, 0.01),
    pos=c('1', '3', '5', '7'),
    subject=unique(df$subject))

predict_subj_ses1 <- sapply(model_ses1, predict, newdata=df_predict,
                            type='response', simplify=F, USE.NAMES=T)
predict_subj_ses2 <- sapply(model_ses2, predict, newdata=df_predict,
                            type='response', simplify=F, USE.NAMES=T)

# add df_predict to each of them
predict_subj_ses1 <- lapply(predict_subj_ses1, function(x) cbind(df_predict, pred=x))
predict_subj_ses2 <- lapply(predict_subj_ses2, function(x) cbind(df_predict, pred=x))

predict_subj_ses1 <- ldply(predict_subj_ses1, data.frame)
```

```r
predict_subj_ses2 <- ldply(predict_subj_ses2, data.frame)
predict_subj_ses1$session <- '1'
predict_subj_ses2$session <- '2'

predict_subj <- rbind(predict_subj_ses1, predict_subj_ses2)
predict_subj <-
  predict_subj %>%
  mutate(morph=morph_resc*100  + 50,
         pos_num=as.numeric(as.character(pos))*45,
         morph_type=.id)
```

Now we can save each individual plot to disk

```r
subjects <- unique(df$subject)
for (subj in subjects) {
  out_dir <- file.path('../img', 'pred_gmm')
  fnout <- file.path(out_dir, paste(subj, '_pred_gmm.png', sep=''))
  # setup dataframes for plotting
  this_subject_df <- df %>%
    filter(subject == subj) %>%
    # add position in angles
    mutate(pos_num=as.numeric(as.character(pos))*45)
  this_predict_subj <- predict_subj %>%
    filter(subject == subj) %>%
    # add position in angles
    mutate(pos_num=as.numeric(as.character(pos))*45)

  plot_curve <-
    this_predict_subj %>%
    # add prediction
    ggplot(aes(morph, pred*100, color=session)) +
    geom_hline(yintercept=50, size=0.5, color='darkgray', alpha=1, linetype='dashed') +
    geom_vline(xintercept=50, size=0.5, color='darkgray', alpha=1, linetype='dashed') +
    geom_line() +
    # add individual data
    stat_summary(data=this_subject_df,
                 aes(morph, response_bin),
                 fun.y=function(x) sum(x)/length(x)*100, geom='point') +
    facet_grid(morph_type ~ pos_num) +
    labs(x='Percentage morphing', y='Percent responses to second identity', color='Session') +
    scale_color_brewer(palette='Set1') +
    ggtitle(paste("Subject", subj)) +
    theme_Publication() +
    coord_equal()

    # save
    dir.create(out_dir, recursive=T)
    ggsave(filename=fnout, plot=plot_curve, width=8, height=6)
}
```

```
## Warning in dir.create(out_dir, recursive = T): '../img/pred_gmm' already
## exists

## Warning in dir.create(out_dir, recursive = T): '../img/pred_gmm' already
```

```
## exists

## Warning in dir.create(out_dir, recursive = T): '../img/pred_gmm' already
## exists

## Warning in dir.create(out_dir, recursive = T): '../img/pred_gmm' already
## exists

## Warning in dir.create(out_dir, recursive = T): '../img/pred_gmm' already
## exists

## Warning in dir.create(out_dir, recursive = T): '../img/pred_gmm' already
## exists

## Warning in dir.create(out_dir, recursive = T): '../img/pred_gmm' already
## exists

## Warning in dir.create(out_dir, recursive = T): '../img/pred_gmm' already
## exists

## Warning in dir.create(out_dir, recursive = T): '../img/pred_gmm' already
## exists

## Warning in dir.create(out_dir, recursive = T): '../img/pred_gmm' already
## exists
```

These are the population-level estimates

```
psep %>%
  ggplot(aes(pos_num, pse*100 + 50, color=session, group=session)) +
  geom_hline(yintercept=50, linetype='dashed', alpha=0.8) +
  geom_point() +
  geom_line() +
  labs(x='Angular location', y='PSE (population)', color='Session') +
  #ggtitle('Population-level PSE') +
  scale_color_brewer(palette='Set1') +
  facet_grid(~morph_type) +
  scale_y_continuous(breaks=c(30, 50, 70)) +
  theme_Publication()
```

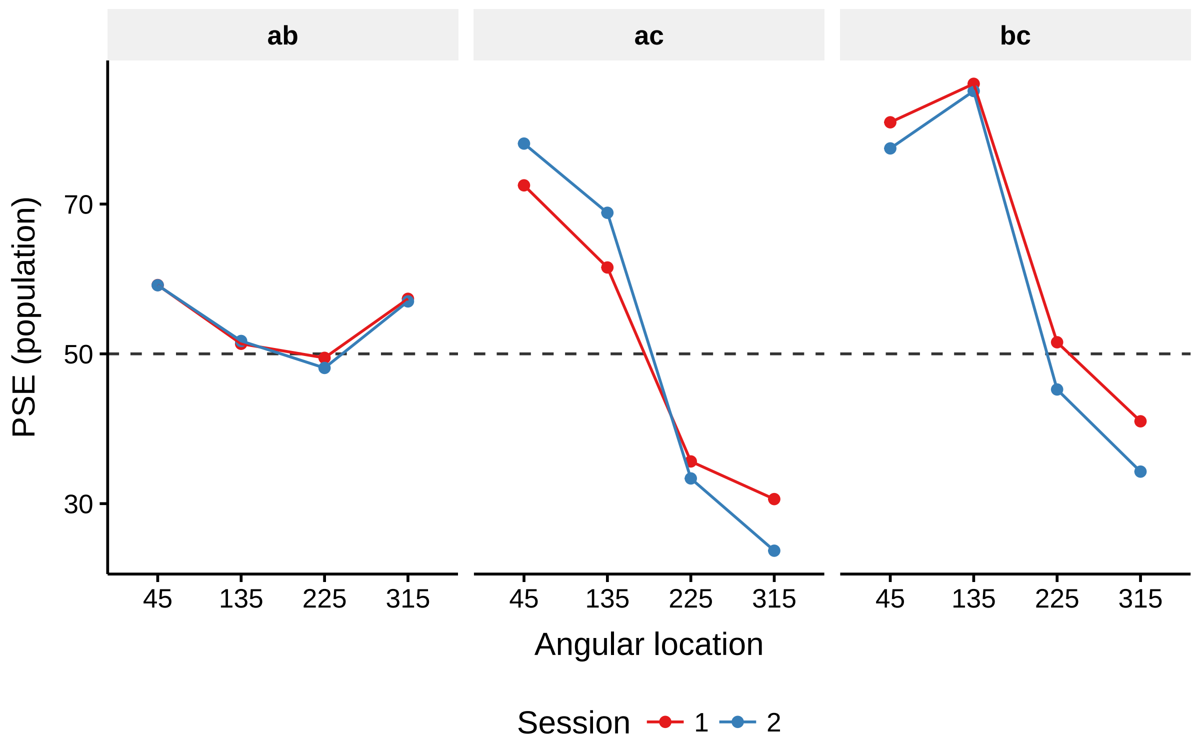

Stability of population level estimates across sessions

Let's compute a correlation across sessions

```
psep_wide <-
psep %>%
  spread(session, pse) %>%
  mutate(session1=.$'1', session2=.$'2')

ggplot(psep_wide, aes(session1*100 + 50, session2*100 + 50, shape=morph_type, group=1)) +
  geom_smooth(method='lm', color='darkgray', se=F) +
  geom_point() +
  labs(x='First measurement (PSE)', y='Second measurement (PSE)', shape='Morph') +
  theme_Publication() +
  coord_equal() +
  #guides(shape=F)
  theme(legend.position=c(0.95, 0.15),
        legend.direction='vertical',
        legend.key.size=unit(.8, 'picas'),
        legend.title=element_text(size=10))
```

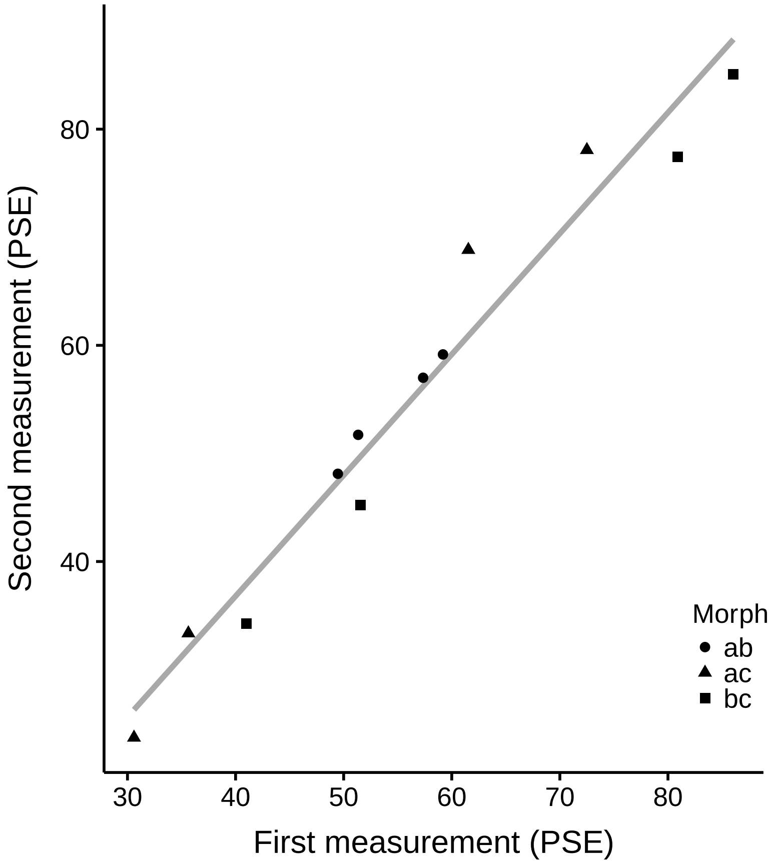


```r
  #coord_equal(xlim=c(20, 95), ylim=c(20, 95)) +
  #scale_x_continuous(breaks=seq(20, 90, 10)) +
  #scale_y_continuous(breaks=seq(20, 90, 10))

ggsave('../img/pse_pop_scatter.png', width=5, height=5)
```

And these are the correlations

```r
set.seed(3432)
bootES(psep_wide[c('session1', 'session2')], R=10000)
```

```
##
## 95.00% bca Confidence Interval, 10000 replicates
## Stat        CI (Low)    CI (High)   bias        SE
## 0.979       0.929       0.992       0.000       0.011
```

```r
cor.test(psep_wide$session1, psep_wide$session2)
```

```
##
##  Pearson's product-moment correlation
##
## data:  psep_wide$session1 and psep_wide$session2
## t = 15.219, df = 10, p-value = 3.042e-08
## alternative hypothesis: true correlation is not equal to 0
## 95 percent confidence interval:
##  0.9248693 0.9942948
## sample estimates:
##       cor
```

```
## 0.9790871
```

They are very consistent across sessions.

Stability of subject-level estimates across sessions

```r
# add morph type
for (morph in morphs) {
  dpse_ses1[[morph]]$subject <- row.names(dpse_ses1[[morph]])
  dpse_ses2[[morph]]$subject <- row.names(dpse_ses2[[morph]])
}
# get dpse in long format for plotting
dpse_ses1_long <-
  ldply(
    lapply(dpse_ses1, function(x) gather(x, pos, pse, -subject)),
    data.frame) %>%
  mutate(morph_type=.id, session='1')
dpse_ses2_long <-
  ldply(
    lapply(dpse_ses2, function(x) gather(x, pos, pse, -subject)),
    data.frame) %>%
  mutate(morph_type=.id, session='2')

dpse <-
  rbind(dpse_ses1_long, dpse_ses2_long) %>%
  mutate(pos_num=mapvalues(pos,
                           paste('pos', c(1, 3, 5, 7), sep=''),
                           c(1, 3, 5, 7)*45))
dpse$pos_num <- factor(dpse$pos_num, levels=c(1, 3, 5, 7)*45)
```

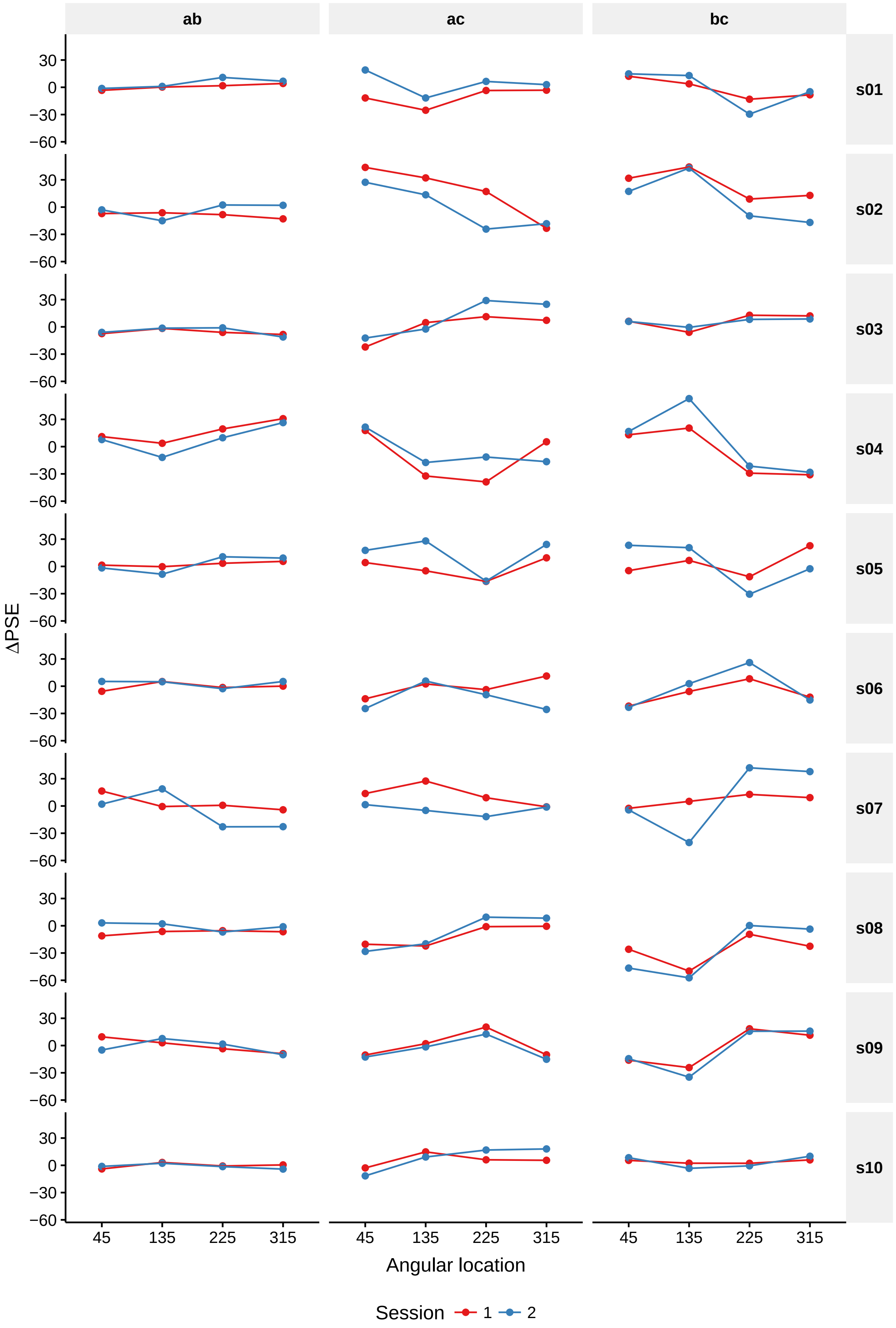
ab
ac
bc
s01
s02
s03
s04
s05
s06
s07
s08
s09
s10
30
0
−30
−60
ΔPSE
45
135
225
315
Angular location
Session
1
2

Aver-

age the estimates across sessions and plot them

```
dpse %>%
  group_by(subject, morph_type, pos_num) %>%
  summarise(pse=mean(pse)) %>%
  ggplot(aes(pos_num, pse*100, color=subject, group=subject)) +
  geom_point(alpha=0.8) +
  geom_line(alpha=0.8) +
  facet_wrap(~morph_type, nrow=1) +
  theme_Publication() +
  labs(x='Angular location', y=TeX('$\\Delta$PSE'), color='Subject') +
  scale_y_continuous(limits=c(-60, 50)) +
  theme(#legend.position=c(0.95, 0.15),
        legend.direction='vertical',
        legend.position='right',
        legend.key.size=unit(.8, 'picas'),
        legend.title=element_text(size=10))
```

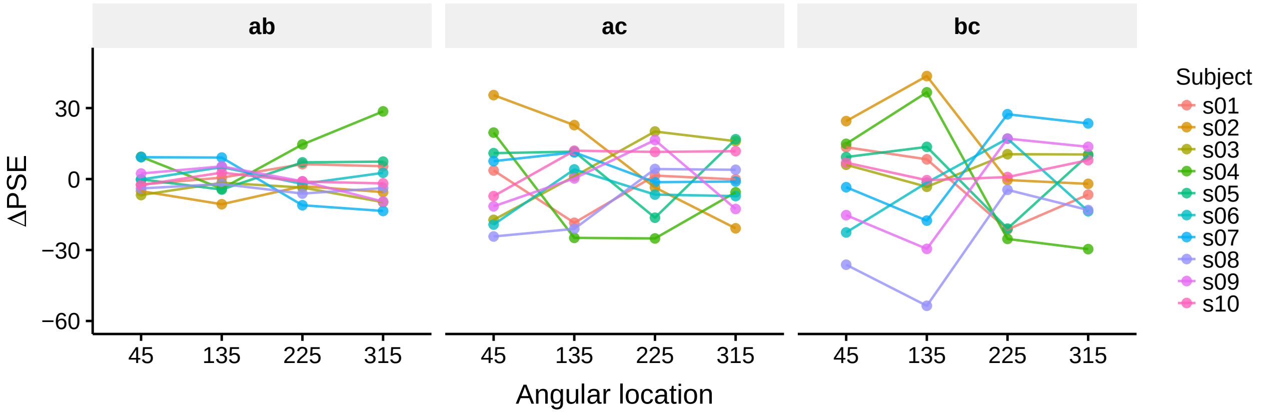


```
ggsave('../img/dpse_subjects.png', width=8, height=3)
```

Let's also plot an example fit for subject s10

```
# get the data to plot individual points
df_s10_plot_ab <-
  df %>%
  filter(subject == 's10', session == '1') %>%
  filter(morph_type == 'ab', pos_num %in% c('225', '315'))
df_s10_plot_ac <-
  df %>%
  filter(subject == 's10', session == '1') %>%
  filter(morph_type == 'ac', pos_num %in% c('135', '315'))
df_s10_plot_bc <-
  df %>%
  filter(subject == 's10', session == '1') %>%
  filter(morph_type == 'bc', pos_num %in% c('135', '315'))

df_s10_plot <-
  rbind(df_s10_plot_ab, df_s10_plot_ac, df_s10_plot_bc)

# now get the pse values to plot
psep1_plot <-
psep %>%
```

```
  filter(session == '1') %>%
  arrange(pos, morph_type)
dpse1_plot <-
dpse %>%
  filter(subject == 's10', session == '1') %>%
  arrange(pos, morph_type) %>%
  mutate(dpse=pse) %>%
  select(morph_type, pos, pos_num, dpse)

pse_s10_plot <- merge(psep1_plot, dpse1_plot)
pse_s10_plot <-
pse_s10_plot %>%
  mutate(tpse=(pse+dpse)*100+50)
```

```
predict_subj %>%
  filter(subject == 's10', session == '1') %>%
  ggplot(aes(morph_resc*100 + 50, pred*100, color=as.factor(pos_num))) +
  geom_segment(aes(x=tpse, xend=tpse, y=-10, yend=50), alpha=0.8, linetype='dashed', data=pse_s10_plot)
  geom_line() +
  facet_wrap(~morph_type, ncol=1) +
  coord_equal(ylim=c(-0.4, 101)) +
  theme_Publication() +
  theme(#legend.position=c(0.95, 0.15),
        legend.direction='vertical',
        legend.position='right',
        legend.key.size=unit(.8, 'picas'),
        legend.title=element_text(size=10)) +
  labs(x='Percentage morphing', y='Percentage responses to\nsecond identity', color='Angular\nlocation')
  stat_summary(aes(morph, response_bin), fun.y=function(x) sum(x)/length(x)*100, geom='point',
               data=df_s10_plot, size=0.9, show.legend=F)
```

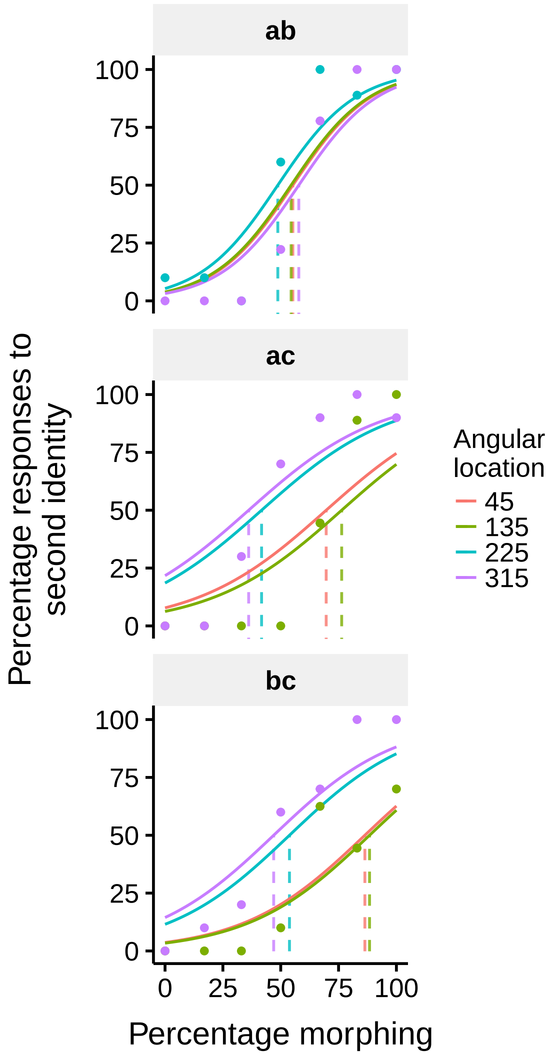


```
ggsave('../img/examplefit_s10.png', width=8, height=6)
```

Correlation of subject-level estimates across sessions

```
dpse_wide <-
dpse %>%
  spread(session, pse) %>%
  mutate(session1=.$'1', session2=.$'2')

ggplot(dpse_wide, aes(session1*100, session2*100, color=subject)) +
  geom_smooth(method='lm', color='darkgray', se=F) +
  geom_point() +
  labs(x=TeX('First measurement ($\\Delta$PSE)'),
       y=TeX('Second measurement ($\\Delta$PSE)'),
       color='Subject') +
  theme_Publication() +
  coord_equal() +
  theme(legend.position=c(0.95, 0.25),
        legend.direction='vertical',
        legend.key.size=unit(.8, 'picas'),
        legend.title=element_text(size=10))
```

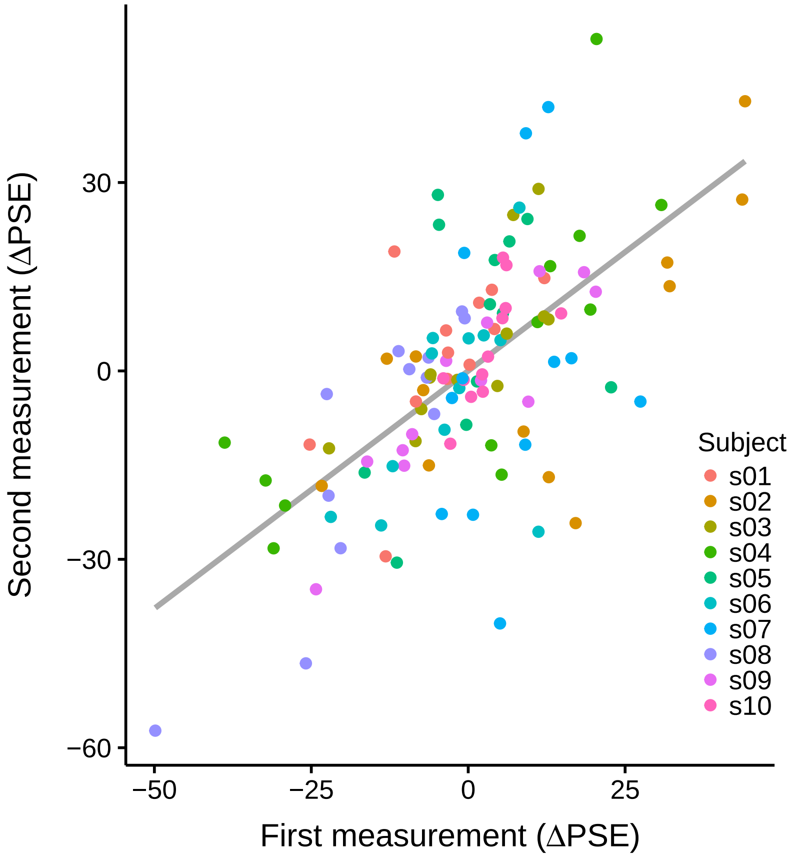


```
  #coord_equal(xlim=c(-60, 45), ylim=c(-60, 45)) +
  #scale_x_continuous(breaks=seq(-60, 40, 20)) +
  #scale_y_continuous(breaks=seq(-60, 40, 20))

ggsave('../img/pse_subj_scatter.png', width=5, height=5)
```

And these are the correlations.

```
set.seed(23448)
bootES(dpse_wide[c('session1', 'session2')], R=10000)
```

```
##
## 95.00% bca Confidence Interval, 10000 replicates
## Stat        CI (Low)    CI (High)   bias        SE
## 0.639       0.499       0.750       -0.003      0.064
```

```
cor.test(dpse_wide$session1, dpse_wide$session2)
```

```
##
##  Pearson's product-moment correlation
##
## data:  dpse_wide$session1 and dpse_wide$session2
## t = 9.0205, df = 118, p-value = 3.997e-15
## alternative hypothesis: true correlation is not equal to 0
## 95 percent confidence interval:
##  0.5190456 0.7340393
## sample estimates:
##       cor
```

```
## 0.6388518
```

Within- vs. between-subjects correlation

Let's compute the correlation between the first and the second session

```
cor_ses12 <- list()
for (morph in morphs) {
  this_cor <- cor(t(dpse_ses1[[morph]][, 1:4]), t(dpse_ses2[[morph]][, 1:4]))
  # make it symmetric
  this_cor <- (this_cor + t(this_cor))/2.
  cor_ses12[[morph]] = this_cor
}

# make a dataframe in long format
make_cor_long <- function(cor) {
  within <- diag(cor)
  between <- cor[lower.tri(cor)]

  df_within_between <- data.frame(corr=c(within, between),
                                  type=c(rep('within', length(within)),
                                         rep('between', length(between)))
                                  )
  return(df_within_between)
}

cor_ses12_long <- sapply(cor_ses12, make_cor_long, simplify=F, USE.NAMES=T)
# add everything together for plotting
cor_ses12_long_plot <- ldply(cor_ses12_long, data.frame)
```

We can check whether the estimates are consistent across sessions, and also subject-specific, by comparing the within-subject correlations with the between-subject correlations. We will compute the bootstrapped difference Within – Between.

```
require(bootES)
require(broom)
```

```
## Loading required package: broom

## Warning: package 'broom' was built under R version 3.2.5
```

```
bootstrap_withinbetween <- function(corr_df) {
  b <- bootES(corr_df,
              data.col='corr', group.col='type',
              contrast=c(within=1, between=-1), R=10000)
  return(b)
}

set.seed(124)
boot_cis <- sapply(cor_ses12_long,
                   bootstrap_withinbetween,
                   simplify=F,
                   USE.NAMES=T)

extract_cis <- function(bootes_out) {
```

```r
  t0 <- bootes_out$t0
  bounds <- bootes_out$bounds
  df <- data.frame(t0=t0, lci=bounds[1], rci=bounds[2])
  return(df)
}

extract_distribution <- function(bootes_out) {
  df <- data.frame(t=bootes_out$t)
  return(df)
}
boot_cis_df <- ldply(boot_cis, extract_cis)
boot_dist_df <- ldply(boot_cis, extract_distribution)
```

And finally plot them

```r
ggplot(data=boot_dist_df, aes(.id, t)) +
  geom_violin(adjust=2) +
  geom_errorbar(data=boot_cis_df, aes(ymin=lci, ymax=rci, y=t0), width=0.01) +
  geom_point(data=boot_cis_df, aes(y=t0)) +
  geom_hline(yintercept=0, linetype='dashed') +
  labs(x='Morph', y='Within - Between subject correlations') +
  theme_Publication() +
  coord_flip() +
  theme(aspect.ratio=3/4)
```

```
## Warning: Ignoring unknown aesthetics: y
```

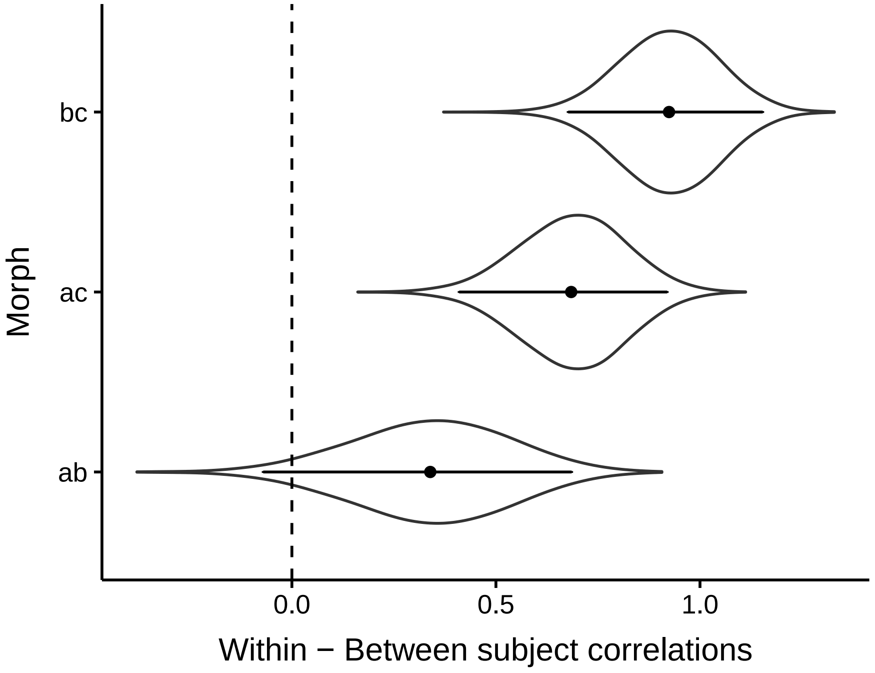

And these are the values

```r
boot_cis_df %>% arrange(.id)
```

```
##   .id        t0         lci       rci
## 1  ab 0.3391053 -0.07029715 0.6855457
## 2  ac 0.6843746  0.40988453 0.9196337
## 3  bc 0.9241400  0.67701408 1.1536785
```

Let's also do it separately for within and between

```
bootstrap <- function(corr_df) {
  b <- bootES(corr_df,
              data.col='corr', R=10000)
  return(b)
}

cor_ses12_long_within <- sapply(cor_ses12_long,
                                function(x) filter(x, type=='within'),
                                simplify=F,
                                USE.NAMES=T)
cor_ses12_long_between <- sapply(cor_ses12_long,
                                function(x) filter(x, type=='between'),
                                simplify=F,
                                USE.NAMES=T)

set.seed(3243)
bs_within <- ldply(
  lapply(cor_ses12_long_within, bootstrap),
  extract_cis)
set.seed(23423)
bs_between <- ldply(
  lapply(cor_ses12_long_between, bootstrap),
  extract_cis)
```

```
bs_within %>% arrange(.id)
```

```
##   .id        t0        lci       rci
## 1  ab 0.3189388 -0.1007527 0.6151277
## 2  ac 0.6179501  0.3513429 0.7855175
## 3  bc 0.8490889  0.6115145 0.9463881
```

```
bs_between %>% arrange(.id)
```

```
##   .id          t0        lci        rci
## 1  ab -0.02016651 -0.1510517 0.10897154
## 2  ac -0.06642447 -0.2118914 0.08072318
## 3  bc -0.07505119 -0.2658810 0.12166054
```

Correlation with familiarity ratings

```
df_quest <- read_csv('../data/questionnaire.csv',
                     col_types='ccddddddddddddd')
```

We are going to create a composite score by simply averaging the questions related to closeness.

```
# add composite score
df_quest <-
df_quest %>%
  # everybody got the name right
  select(-name) %>%
  rowwise() %>%
  # Let's make a composite score by averaging ios, wescale, sci1, sci2
  mutate(compscore=mean(c(ios, wescale, sci1, sci2)))
```

Let's start simply by looking at the average values for each id

```
df_quest_long <-
df_quest %>%
  gather(scale, value, -subject, -stim)
```

```
df_quest_long %>%
  ggplot(aes(scale, value, color=stim)) +
  stat_summary(fun.data=mean_cl_boot, geom='errorbar', width=0.1, position=position_dodge(w=0.3)) +
  stat_summary(fun.y=mean, geom='point', position=position_dodge(w=0.3)) +
  theme_Publication() +
  theme(axis.text.x=element_text(angle=45, hjust=1))
```

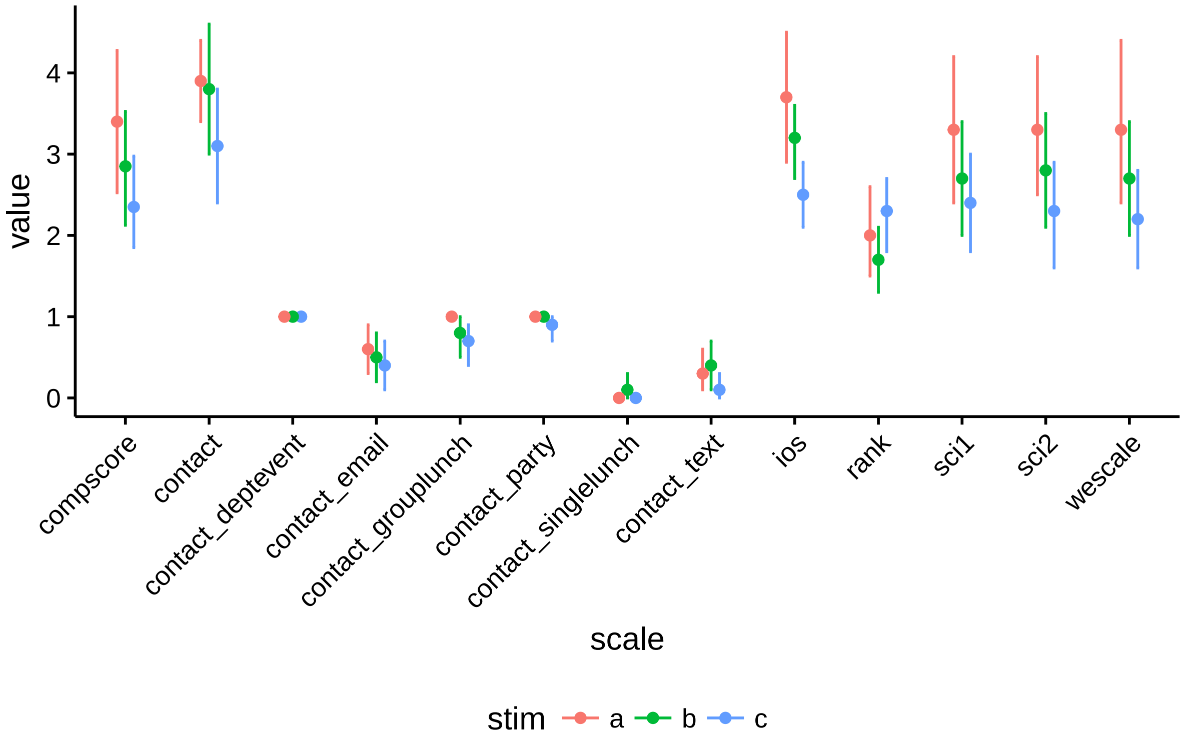


```
ggsave('../img/scores.png', width=8, height=6)
```

```
# compute a score of deviance from 0 for each subject and morph type
# basically the variance
dpse_deviance <-
dpse %>%
  group_by(subject, morph_type, pos_num) %>%
  summarise(pse=mean(pse)) %>%
  group_by(subject, morph_type) %>%
  summarise(psescore=sum(pse^2))

# now for each subject compute the average score for the morphs
df_quest_avg <-
df_quest %>%
  select(subject, stim, compscore) %>%
  spread(stim, compscore) %>%
  rowwise() %>%
  mutate(score_ab=mean(c(a, b)),
         score_bc=mean(c(b, c)),
```

```r
          score_ac=mean(c(a, c))) %>%
  select(-a, -b, -c) %>%
  gather(morph_type, score, -subject)

df_quest_avg$morph_type <-
  mapvalues(df_quest_avg$morph_type,
            c('score_ab', 'score_ac', 'score_bc'),
            c('ab', 'ac', 'bc'))
df_quest_deviance <-
df_quest_avg %>%
  arrange(subject, morph_type) %>%
  merge(dpse_deviance)
```

```r
ggplot(aes(score, psescore, color=morph_type), data=df_quest_deviance) +
  geom_smooth(method='lm', aes(group=1), se=F, color='darkgray') +
  geom_point() +
  theme_Publication() +
  coord_fixed(ratio=10) +
  labs(x='Familiarity score', y=TeX('$\\Delta$PSE variance'), color='Morph type') +
  theme(legend.position=c(0.95, 0.6),
        legend.direction='vertical',
        legend.key.size=unit(.8, 'picas'),
        legend.title=element_text(size=10)) +
  scale_color_brewer(palette='Set2')
```

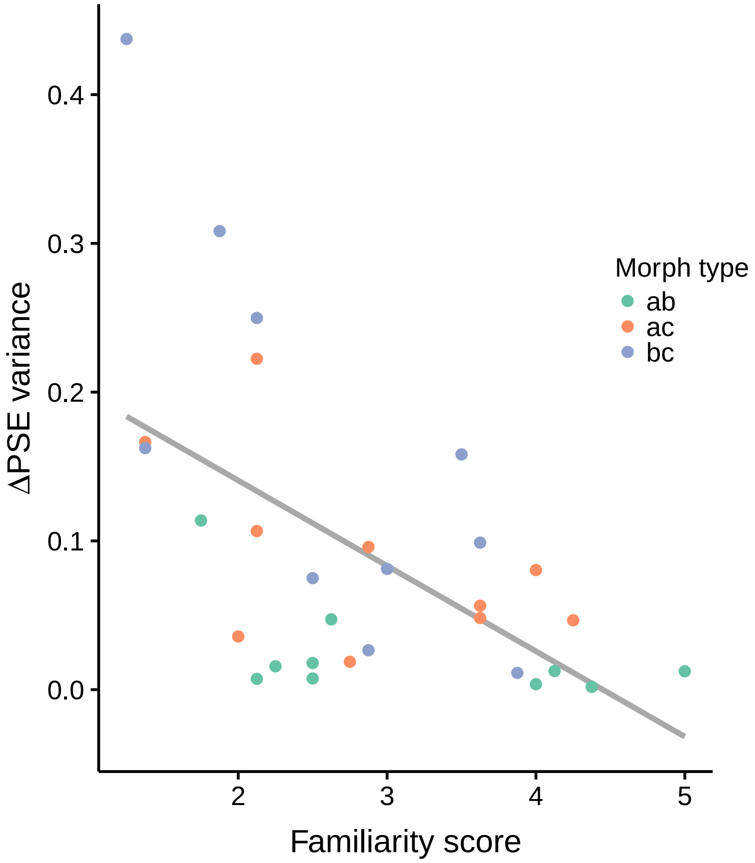

```r
ggsave('../img/psequest_scatter.png', width=5, height=5)
```

```r
cor.test(df_quest_deviance$score, df_quest_deviance$psescore)
```

```
##
##  Pearson's product-moment correlation
##
## data:  df_quest_deviance$score and df_quest_deviance$psescore
## t = -3.5895, df = 28, p-value = 0.001248
## alternative hypothesis: true correlation is not equal to 0
## 95 percent confidence interval:
##  -0.7666035 -0.2520935
## sample estimates:
##        cor
## -0.5613776
```

```r
set.seed(324)
bootES(df_quest_deviance[c('score', 'psescore')], R=10000)
```

```
##
## 95.00% bca Confidence Interval, 10000 replicates
## Stat        CI (Low)    CI (High)   bias        SE
## -0.561      -0.708      -0.298      0.006       0.100
```

Is it personal familiarity or is it contact?

```r
df_quest_contact_avg <-
df_quest %>%
  select(subject, stim, contact) %>%
  spread(stim, contact) %>%
  rowwise() %>%
  mutate(ab=mean(c(a, b)),
         bc=mean(c(b, c)),
         ac=mean(c(a, c))) %>%
  select(-a, -b, -c) %>%
  gather(morph_type, contact, -subject)

df_contact_deviance <-
df_quest_deviance %>%
  arrange(subject, morph_type) %>%
  merge(df_quest_contact_avg)
```

Are familiarity score and contact correlated?

```r
cor.test(df_contact_deviance$score, df_contact_deviance$contact)
```

```
##
##  Pearson's product-moment correlation
##
## data:  df_contact_deviance$score and df_contact_deviance$contact
## t = 2.6517, df = 28, p-value = 0.01304
## alternative hypothesis: true correlation is not equal to 0
## 95 percent confidence interval:
##  0.1046298 0.6959508
```

```
## sample estimates:
##       cor
## 0.4480113
```

```r
set.seed(324)
bootES(df_contact_deviance[c('score', 'contact')], R=10000)
```

```
##
## 95.00% bca Confidence Interval, 10000 replicates
## Stat        CI (Low)    CI (High)   bias        SE
## 0.448       0.168       0.675       -0.002      0.130
```

Run a model that predicts the variance of ΔPSE using only the familiarity score and then also the contact score.

```r
m1 <- lm(psescore ~ score, data=df_contact_deviance)
m2 <- update(m1, . ~ . + contact)

tidy(m1)
```

```
##          term    estimate  std.error statistic      p.value
## 1 (Intercept)  0.25543170 0.04848293  5.268487 1.331582e-05
## 2       score -0.05740843 0.01599341 -3.589506 1.248105e-03
```

```r
tidy(m2)
```

```
##          term    estimate  std.error statistic      p.value
## 1 (Intercept)  0.36532810 0.06236289  5.858101 3.077435e-06
## 2       score -0.03894801 0.01640093 -2.374744 2.492620e-02
## 3     contact -0.04522674 0.01800160 -2.512374 1.827090e-02
```

```r
anova(m1, m2)
```

```
## Analysis of Variance Table
##
## Model 1: psescore ~ score
## Model 2: psescore ~ score + contact
##   Res.Df     RSS Df Sum of Sq     F   Pr(>F)
## 1     28 0.20880
## 2     27 0.16924  1  0.039565 6.312 0.01827 *
## ---
## Signif. codes:  0 '***' 0.001 '**' 0.01 '*' 0.05 '.' 0.1 ' ' 1
```

```r
summary(m1)
```

```
##
## Call:
## lm(formula = psescore ~ score, data = df_contact_deviance)
##
## Residuals:
##       Min        1Q    Median        3Q       Max
## -0.126183 -0.053446 -0.008043  0.041767  0.253693
##
## Coefficients:
##             Estimate Std. Error t value Pr(>|t|)
## (Intercept)  0.25543    0.04848   5.268 1.33e-05 ***
## score       -0.05741    0.01599  -3.590  0.00125 **
## ---
```

```
## Signif. codes:  0 '***' 0.001 '**' 0.01 '*' 0.05 '.' 0.1 ' ' 1
##
## Residual standard error: 0.08636 on 28 degrees of freedom
## Multiple R-squared:  0.3151, Adjusted R-squared:  0.2907
## F-statistic: 12.88 on 1 and 28 DF,  p-value: 0.001248
```

```
summary(m2)
```

```
##
## Call:
## lm(formula = psescore ~ score + contact, data = df_contact_deviance)
##
## Residuals:
##       Min        1Q    Median        3Q       Max
## -0.139628 -0.057285 -0.005151  0.039722  0.211174
##
## Coefficients:
##             Estimate Std. Error t value Pr(>|t|)
## (Intercept)  0.36533    0.06236   5.858 3.08e-06 ***
## score       -0.03895    0.01640  -2.375   0.0249 *
## contact     -0.04523    0.01800  -2.512   0.0183 *
## ---
## Signif. codes:  0 '***' 0.001 '**' 0.01 '*' 0.05 '.' 0.1 ' ' 1
##
## Residual standard error: 0.07917 on 27 degrees of freedom
## Multiple R-squared:  0.4449, Adjusted R-squared:  0.4038
## F-statistic: 10.82 on 2 and 27 DF,  p-value: 0.0003539
```

```
require(heplots)
```

```
## Loading required package: heplots
```

```
## Warning: package 'heplots' was built under R version 3.2.5
```

```
## Loading required package: car
```

```
## Warning: package 'car' was built under R version 3.2.5
```

```
##
## Attaching package: 'car'
```

```
## The following object is masked from 'package:dplyr':
##
##     recode
```

```
## The following object is masked from 'package:purrr':
##
##     some
```

```
## The following object is masked from 'package:boot':
##
##     logit
```

```
round(etasq(m1), 2)
```

```
##           Partial eta^2
## score              0.32
## Residuals            NA
```

```r
round(etasq(m2), 2)
```

```
##           Partial eta^2
## score              0.17
## contact            0.19
## Residuals            NA
```

```r
# from http://www.statmethods.net/advstats/bootstrapping.html
bs <- function(formula, data, indices) {
  d <- data[indices,] # allows boot to select sample
  fit <- lm(formula, data=d)
  return(coef(fit))
}

set.seed(234)
results <- boot(data=df_contact_deviance, statistic=bs,
                R=10000, formula=psescore ~ score + contact)

t0 <- results$t0
conf <- c()
for (i in 1:length(t0)) {
  b <- boot.ci(results, type='bca', index=i)
  conf <- rbind(conf, b$bca[4:5])
}

df_ci <- data.frame(t0=t0, lci=conf[,1], rci=conf[,2])
```

```r
df_ci
```

```
##                      t0         lci         rci
## (Intercept)  0.36532810  0.21465489  0.55147559
## score       -0.03894801 -0.07431666 -0.01631785
## contact     -0.04522674 -0.08609399 -0.01694056
```

Now compute partial correlations

```r
partialout_variable <- function(df, x, y, z) {
  mx <- lm(df[[x]] ~ df[[z]])
  my <- lm(df[[y]] ~ df[[z]])
  df_out <- data.frame(mx$residuals, my$residuals)
  names(df_out) <- c(x, y)
  return(df_out)
}

# Make plots of partial correlation with residuals
partial_contact <- partialout_variable(df_contact_deviance, "psescore", "score", "contact")
partial_score <- partialout_variable(df_contact_deviance, "psescore", "contact", "score")
cor.test(partial_contact$psescore, partial_contact$score)
```

```
##
##  Pearson's product-moment correlation
##
## data:  partial_contact$psescore and partial_contact$score
## t = -2.4183, df = 28, p-value = 0.02235
## alternative hypothesis: true correlation is not equal to 0
## 95 percent confidence interval:
```

```
##  -0.67487275 -0.06515499
## sample estimates:
##        cor
## -0.4156671
```

```r
cor.test(partial_score$psescore, partial_score$contact)
```

```
##
##  Pearson's product-moment correlation
##
## data:  partial_score$psescore and partial_score$contact
## t = -2.5585, df = 28, p-value = 0.01621
## alternative hypothesis: true correlation is not equal to 0
## 95 percent confidence interval:
##  -0.68771457 -0.08898003
## sample estimates:
##        cor
## -0.4352951
```

```r
set.seed(3242)
bootES(partial_contact[c('psescore', 'score')], R=10000)
```

```
##
## 95.00% bca Confidence Interval, 10000 replicates
## Stat        CI (Low)    CI (High)   bias        SE
## -0.416      -0.610      -0.158      0.008       0.116
```

```r
bootES(partial_score[c('psescore', 'contact')], R=10000)
```

```
##
## 95.00% bca Confidence Interval, 10000 replicates
## Stat        CI (Low)    CI (High)   bias        SE
## -0.435      -0.619      -0.166      -0.001      0.113
```

```r
# add the morph types to partial_contact and partial_score
partial_contact$morph_type <- df_contact_deviance$morph_type
partial_score$morph_type <- df_contact_deviance$morph_type
```

```r
# just a theme to uniform scatters
theme_Publication_scatter <- function(base_size=12) {
  (theme_Publication(base_size=base_size) +
  theme(legend.position=c(0.95, 0.6),
        legend.direction='vertical',
        legend.key.size=unit(.8, 'picas'),
        legend.title=element_text(size=10),
        axis.title=element_text(size=10)))
}
```

```r
ggplot(aes(score, psescore, color=morph_type), data=df_quest_deviance) +
  geom_smooth(method='lm', aes(group=1), se=F, color='darkgray') +
  geom_point() +
  coord_fixed(ratio=10) +
  labs(x='Familiarity score', y=TeX('$\\Delta$PSE variance'), color='Morph type') +
  theme_Publication_scatter() +
  scale_color_brewer(palette='Set2')
```

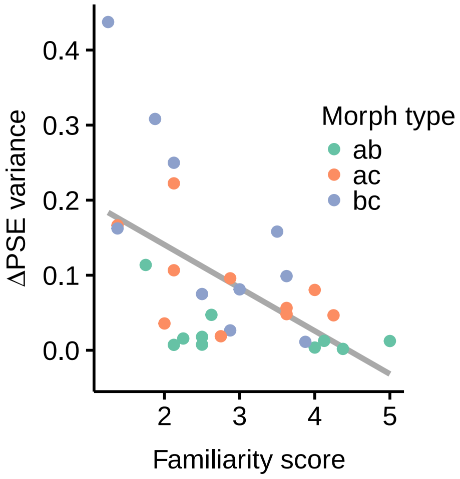


```
ggsave('../img/psequest_scatter_psefam.png', width=3, height=3)
```

```
ggplot(aes(score, psescore, color=morph_type), data=partial_contact) +
  geom_smooth(method='lm', aes(group=1), se=F, color='darkgray') +
  geom_point() +
  coord_fixed(ratio=10) +
  labs(x='Familiarity score | Contact', y=TeX('$\\Delta$PSE variance | Contact'), color='Morph type') +
  theme_Publication_scatter() +
  scale_color_brewer(palette='Set2')
```

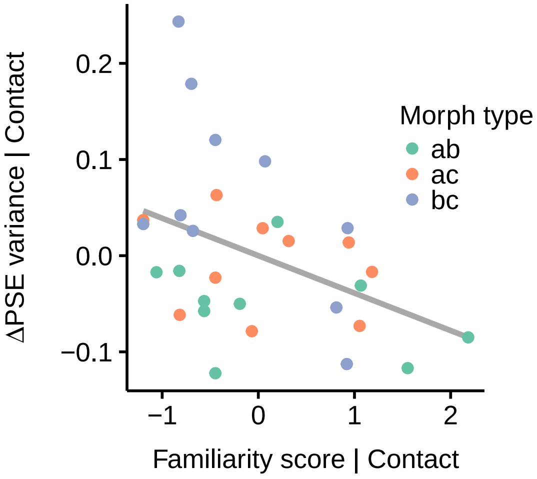


```
ggsave('../img/psequest_scatter_psefam_contact.png', width=3, height=3)
```

```
ggplot(aes(contact, psescore, color=morph_type), data=partial_score) +
  geom_smooth(method='lm', aes(group=1), se=F, color='darkgray') +
  geom_point() +
  coord_fixed(ratio=10, expand=T) +
  labs(x='Contact score | Familiarity', y=TeX('$\\Delta$PSE variance | Familiarity'), color='Morph type
  theme_Publication_scatter() +
  scale_color_brewer(palette='Set2')
```

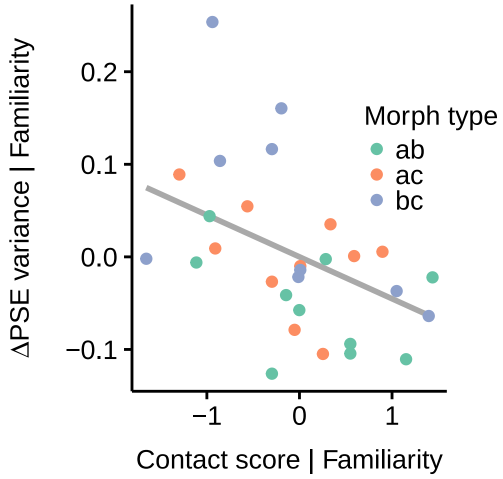


```
ggsave('../img/psequest_scatter_psecontact_fam.png', width=3, height=3)
```

PSE for individual identities

We are going to evaluate the bias towards specific identities, instead of pairs of morphs. To do so, we'll fit a linear model predicting the PSE, taking the data of each pair of morphs containing the same identity. For example, to evaluate the bias for identity `a`, we'll consider the PSE estimates for morphs `ab` and `ac` across the two sessions, and fit a linear model with participants, angular location, and their interaction as predictors.

```
require(tidyverse)
require(lme4)
require(car)
require(broom)
```

First we compute the PSE for each participant by adding PSEp and DPSE.

```
cols <- c('pos1', 'pos3', 'pos5', 'pos7')
pse_ses1 = list()
pse_ses2 = list()

# compute the PSE by adding PSEp to DPSE for each subject
for (morph in c('ac', 'ab', 'bc')) {
  psep <- psep_ses1[[morph]]
  dpse <- dpse_ses1[[morph]]
  pse <- (matrix(rep(psep[cols], 10), nrow=10, byrow=T) +
                     dpse[cols])*100 + 50
  pse$subject <- row.names(pse)
  pse_ses1[[morph]] <- pse

  psep <- psep_ses2[[morph]]
  dpse <- dpse_ses2[[morph]]
  pse <- (matrix(rep(psep[cols], 10), nrow=10, byrow=T) +
                     dpse[cols])*100 + 50
  pse$subject <- row.names(pse)
  pse_ses2[[morph]] <- pse
}

# now store it in long format
```

```
pse_ses1_long <-
  ldply(
    lapply(pse_ses1, function(x) mutate(x, subject=rownames(x)) %>% gather(pos, pse, -subject)),
    data.frame) %>%
  mutate(morph_type=.id, session='1')
pse_ses2_long <-
  ldply(
    lapply(pse_ses2, function(x) mutate(x, subject=rownames(x)) %>% gather(pos, pse, -subject)),
    data.frame) %>%
  mutate(morph_type=.id, session='2')

pse <-
  rbind(pse_ses1_long, pse_ses2_long) %>%
  mutate(pos_num=mapvalues(pos,
                           paste('pos', c(1, 3, 5, 7), sep=''),
                           c(1, 3, 5, 7)*45))
pse$pos_num <- factor(pse$pos_num, levels=c(1, 3, 5, 7)*45)
```

Let's save it for later use.

```
# save pse
pse_fn <- '../derivatives/pse.csv'

if (!file.exists(pse_fn)) {
  pse_ <- pse %>%
    select(subject, session, morph_type, pos_num, pse)
  write_csv(pse_, pse_fn)
}
```

Here we load the pre-estimated data

```
pse <- read_csv('../derivatives/pse.csv', col_types='ciccd')
pse$morph_type <- as.factor(pse$morph_type)
pse$pos_num <- factor(pse$pos_num,
                      levels=c('45', '135', '225', '315'))
```

We set up a function to filter the data for each individual identity. Moreover, we make sure that the PSE value indicates consistently the bias with respect to the same identity.

```
filter_dataset <- function(id, df) {
  identity2morphs <- list(a=c('ab', 'ac'),
                          b=c('ab', 'bc'),
                          c=c('bc', 'ac'))
  df_ <-
    df %>%
    filter(morph_type %in% identity2morphs[[id]]) %>%
    droplevels(.$morph_type)

  # put the PSE in the right order: > 50 indicates
  # more towards the identity of interest
  if (id == 'b') {
    df_ <-
    df_ %>%
      mutate(pse=ifelse(morph_type == 'ab', 100 - pse, pse))
  } else if (id == 'c') {
    df_ <-
```

```
    df_ %>%
      mutate(pse=100 - pse)
  }

  return(df_)
}
```

Finally we can fit the models

```
ids <- c('a', 'b', 'c')
# split the datasets
pse_ids <- map(ids, filter_dataset, pse)

fit_model <- function(df) {
  df$subject <- as.factor(df$subject)
  df$pos_num <- as.factor(df$pos_num)
  contrasts(df$subject) <- contr.sum(length(unique(df$subject)))
  contrasts(df$pos_num) <- contr.sum(length(unique(df$pos_num)))

  return(lm(pse ~ subject * pos_num, data=df))
}

model_ids <-
  map(pse_ids, fit_model) %>%
  set_names(ids)

anova_model <-
  model_ids %>%
  map(Anova, type=3)

tidy_anova <-
  anova_model %>%
  map_df(tidy, .id='id')
```

And here are the results

```
anova_model
```

```
## $a
## Anova Table (Type III tests)
##
## Response: pse
##                 Sum Sq  Df   F value    Pr(>F)
## (Intercept)     438129   1 1501.3371 < 2.2e-16 ***
## subject           1395   9    0.5311    0.8495
## pos_num          18777   3   21.4481  3.43e-11 ***
## subject:pos_num   9191  27    1.1664    0.2807
## Residuals        35019 120
## ---
## Signif. codes:  0 '***' 0.001 '**' 0.01 '*' 0.05 '.' 0.1 ' ' 1
##
## $b
## Anova Table (Type III tests)
##
## Response: pse
```

```
##                  Sum Sq  Df   F value    Pr(>F)
## (Intercept)     469993   1 1184.2655 < 2.2e-16 ***
## subject           5970   9    1.6713   0.10322
## pos_num          16226   3   13.6287 1.039e-07 ***
## subject:pos_num  18981  27    1.7714   0.01947 *
## Residuals        47624 120
## ---
## Signif. codes:  0 '***' 0.001 '**' 0.01 '*' 0.05 '.' 0.1 ' ' 1
##
## $c
## Anova Table (Type III tests)
##
## Response: pse
##                  Sum Sq  Df   F value    Pr(>F)
## (Intercept)     302383   1 1093.0426 < 2.2e-16 ***
## subject          10701   9    4.2981 7.363e-05 ***
## pos_num          62758   3   75.6183 < 2.2e-16 ***
## subject:pos_num  24919  27    3.3361 3.229e-06 ***
## Residuals        33197 120
## ---
## Signif. codes:  0 '***' 0.001 '**' 0.01 '*' 0.05 '.' 0.1 ' ' 1
```

We find a significant interaction between participants and angular location for identities `b` and `c`, but not for identity `a`, suggesting that for identity `a` the bias is consistently more homogeneous across participants.

Use the model to predict the biases

We can also use these models now to predict the biases at each angular location for each individual participant.

```r
df_pred <- expand.grid(
  subject=unique(pse$subject),
  pos_num=unique(pse$pos_num)
)

df_pred <-
map_df(model_ids, predict, df_pred) %>%
  gather(identity, pse) %>%
  cbind(df_pred, .)
```

```r
ggplot(aes(pos_num, pse-50, color=subject, group=subject), data=df_pred) +
  geom_point() +
  geom_line() +
  facet_wrap(~identity, nrow=1)
```

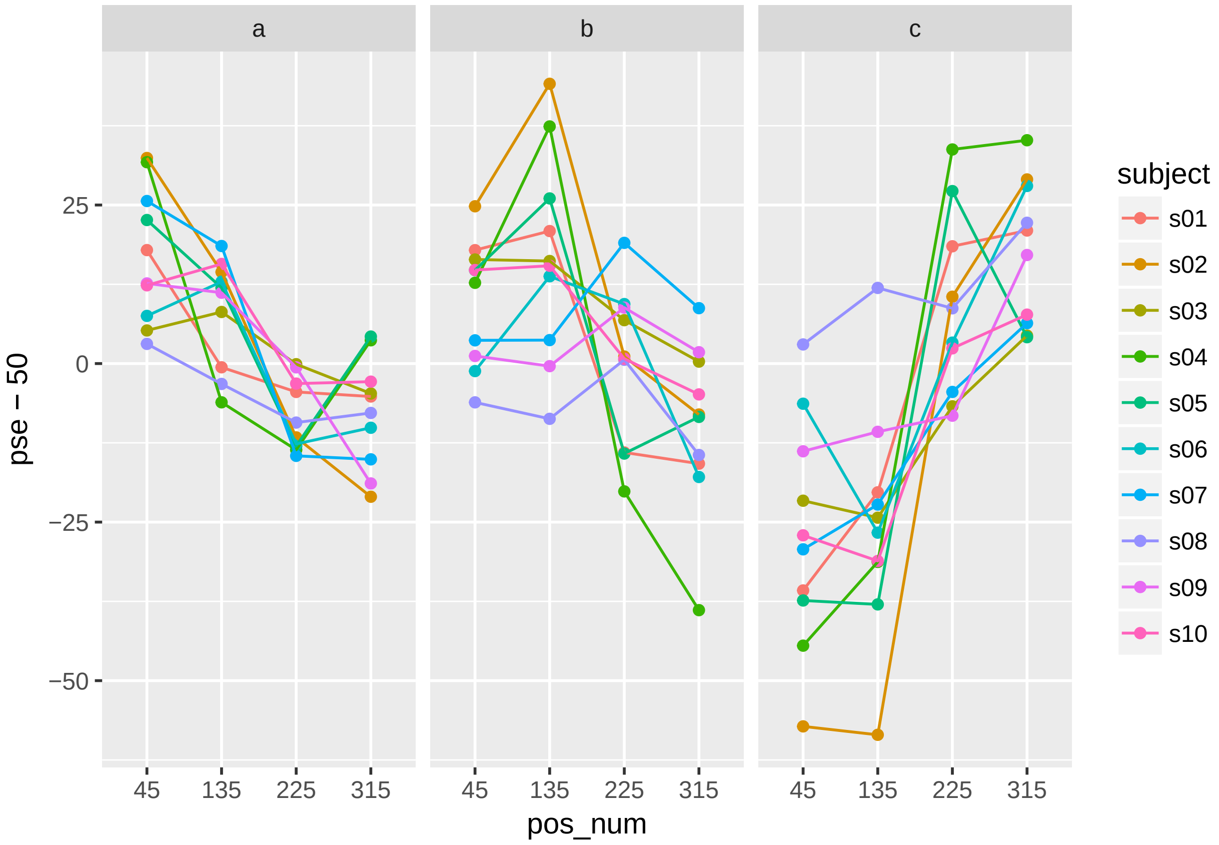
a
b
c
25
0
−25
−50
pse − 50
45
135
225
315
pos_num
subject
s01
s02
s03
s04
s05
s06
s07
s08
s09
s10
