# Supplementary material for: Idiosyncratic, Retinotopic Bias in Face Identification Modulated by Familiarity
Source: eNeuro. 2018 Oct 4;5(5):ENEURO.0054-18.2018. doi: 10.1523/ENEURO.0054-18.2018 (PMC6171739; doi:10.1523/ENEURO.0054-18.2018)
Supplement: Extended Data — The archive contains data from both experiments, as well as the analysis scripts. Download Extended Data 1, ZIP file. [file sup_enu-eN-NWR-0054-18-s02.zip › famretino2-3.0.0/model/plot_simdata.nb.html]

Plotting modeling results - increase in gain and units


Code 

- Show All Code
- Hide All Code
- Download Rmd

# Plotting modeling results - increase in gain and units


```
require(tidyverse)
```


```
# task <- 'estimation'  # or 'face'
task <- 'face'  # or 'face'
output_fn <- paste('outputs/sim-03_task-', task, '_cis.csv', sep='')
imgfn <- paste('img/sim-03_task-', task, sep='')
input_fn <-  paste('outputs/sim-03_task-', task, 
        '_increase_units_increase_gain_500sim.csv', sep='')
df <- read_csv(input_fn)
```


```
Missing column names filled in: 'X1' [1]Parsed with column specification:
cols(
  X1 = col_integer(),
  roi = col_character(),
  ratio = col_integer(),
  n_voxels = col_integer(),
  gain = col_double(),
  rf_increase = col_double(),
  value = col_double(),
  b_units = col_integer()
)
```


```
df$ratio <- as.factor(df$ratio)
df <- df %>% group_by(ratio, roi, gain) %>% select(-X1)
```


# generate bootstrapped values for median and cis


```
theme_Publication <- function(base_size=12) {
      library(ggthemes)
      (theme_foundation(base_size=base_size)
       + theme(plot.title = element_text(face = "bold",
                                         size = rel(1.2), hjust = 0.5),
               text = element_text(),
               panel.background = element_rect(colour = NA),
               plot.background = element_rect(colour = NA),
               panel.border = element_rect(colour = NA),
               axis.title = element_text(size = rel(1)),
               axis.title.y = element_text(angle=90,vjust =2),
               axis.title.x = element_text(vjust = -0.2),
               axis.text = element_text(), 
               axis.line = element_line(colour="black"),
               axis.ticks = element_line(),
               panel.grid.major = element_blank(), #element_line(colour="#f0f0f0"),
               panel.grid.minor = element_blank(),
               legend.key = element_rect(colour = NA),
               legend.position = "bottom",
               legend.direction = "horizontal",
               #legend.key.size= unit(0.2, "cm"),
               legend.spacing = unit(0, "cm"),
               legend.title = element_text(),
               plot.margin = unit(c(10,5,5,5),"mm"),
               strip.background = element_rect(colour="#f0f0f0",fill="#f0f0f0"),
               strip.text = element_text(face="bold"),
               strip.text.y = element_text(angle = 0)
          ))
      
}
```


```
df_t0_gain1 <- df_t0 %>%
  filter(gain == 1.0)
if (task == 'estimation') {
  y_lims <- c(0, 1.25)
} else {
  y_lims <- c(0, 0.8)
}
```


```
df_t0_gain1 %>%
  ggplot(aes(ratio, value, ymin=lo, ymax=hi, color=roi, group=roi)) +
  geom_linerange(show.legend=F) +
  geom_line(show.legend=F) +
  geom_point(size=1.5) +
  labs(x="Ratio of units selective to identity a",
       y="Simulated PSE variance",
       color="") +
  theme_Publication() +
  theme(legend.direction='vertical',
        legend.position=c(0.8, 0.8)) +
  scale_color_brewer(palette='Set1') +
  coord_cartesian(ylim=y_lims)
```


```
img <- paste(imgfn, '_data.png', sep='')
ggsave(img, dpi=300, height=3, width=4)
```


```
# make my own palettes to avoid too light colors
library(RColorBrewer)
red <- brewer.pal(n=9, "Reds")
red_palette <- colorRampPalette(c(red[3], red[6], red[9]), space = "Lab")
my_red <- red_palette(7)
blue <- brewer.pal(n=9, "Blues")
blue_palette <- colorRampPalette(c(blue[3], blue[6], blue[9]), space = "Lab")
my_blue <- blue_palette(7)
green <- brewer.pal(n=9, "Greens")
green_palette <- colorRampPalette(c(green[3], green[6], green[9]), space = "Lab")
my_green <- green_palette(7)
```


```
df_t0_iog <- filter(df_t0, roi == 'IOG')
df_t0_iog$gain <- as.factor(df_t0_iog$gain)
df_t0_iog %>%
  ggplot(aes(ratio, value, color=gain, group=gain)) +
  geom_line(show.legend=F) +
  geom_point(size=1.5) +
  labs(x="Ratio of units selective to identity a",
       y="Simulated PSE variance",
       color="Gain a/b") +
  theme_Publication(base_size=10) +
  theme(legend.direction='vertical',
        legend.position=c(0.9, 0.65),
        legend.title=element_text(size=10),
        legend.key.size=unit(10, 'pt'),
        legend.text=element_text(size=8)) + 
  scale_color_manual(values=my_red) +
  coord_cartesian(ylim=y_lims)
```


```
img <- paste(imgfn, '_increase-gain_iog.png', sep='')
ggsave(img, dpi=300, height=2.25, width=3.)
```


```
df_t0_pfus <- filter(df_t0, roi == 'pFus')
df_t0_pfus$gain <- as.factor(df_t0_pfus$gain)
df_t0_pfus %>%
  ggplot(aes(ratio, value, color=gain, group=gain)) +
  geom_line(show.legend=F) +
  geom_point(size=1.5) +
  labs(x="Ratio of units selective to identity a",
       y="Simulated PSE variance",
       color="Gain a/b") +
  theme_Publication(base_size=10) +
  theme(legend.direction='vertical',
        legend.position=c(0.9, 0.65),
        legend.title=element_text(size=10),
        legend.key.size=unit(10, 'pt'),
        legend.text=element_text(size=8)) + 
  scale_color_manual(values=my_blue) +
  coord_cartesian(ylim=y_lims)
```


```
img <- paste(imgfn, '_increase-gain_pfus.png', sep='')
ggsave(img, dpi=300, height=2.25, width=3.)
```


```
df_t0_mfus <- filter(df_t0, roi == 'mFus')
df_t0_mfus$gain <- as.factor(df_t0_mfus$gain)
df_t0_mfus %>%
  ggplot(aes(ratio, value, color=gain, group=gain)) +
  geom_line(show.legend=F) +
  geom_point(size=1.5) +
  labs(x="Ratio of units selective to identity a",
       y="Simulated PSE variance",
       color="Gain a/b") +
  theme_Publication(base_size=10) +
  theme(legend.direction='vertical',
        legend.position=c(0.9, 0.65),
        legend.title=element_text(size=10),
        legend.key.size=unit(10, 'pt'),
        legend.text=element_text(size=8)) + 
  scale_color_manual(values=my_green) +
  coord_cartesian(ylim=y_lims)
```


```
img <- paste(imgfn, '_increase-gain_mfus.png', sep='')
ggsave(img, dpi=300, height=2.25, width=3.)
```

LS0tCnRpdGxlOiAiUGxvdHRpbmcgbW9kZWxpbmcgcmVzdWx0cyAtIGluY3JlYXNlIGluIGdhaW4gYW5kIHVuaXRzIgpvdXRwdXQ6IGh0bWxfbm90ZWJvb2sKLS0tCmBgYHtyIG1lc3NhZ2U9RkFMU0UsIHdhcm5pbmc9RkFMU0V9CnJlcXVpcmUodGlkeXZlcnNlKQpgYGAKCmBgYHtyfQojIHRhc2sgPC0gJ2VzdGltYXRpb24nICAjIG9yICdmYWNlJwp0YXNrIDwtICdmYWNlJyAgIyBvciAnZmFjZScKb3V0cHV0X2ZuIDwtIHBhc3RlKCdvdXRwdXRzL3NpbS0wM190YXNrLScsIHRhc2ssICdfY2lzLmNzdicsIHNlcD0nJykKaW1nZm4gPC0gcGFzdGUoJ2ltZy9zaW0tMDNfdGFzay0nLCB0YXNrLCBzZXA9JycpCmlucHV0X2ZuIDwtICBwYXN0ZSgnb3V0cHV0cy9zaW0tMDNfdGFzay0nLCB0YXNrLCAKICAgICAgICAnX2luY3JlYXNlX3VuaXRzX2luY3JlYXNlX2dhaW5fNTAwc2ltLmNzdicsIHNlcD0nJykKZGYgPC0gcmVhZF9jc3YoaW5wdXRfZm4pCmRmJHJhdGlvIDwtIGFzLmZhY3RvcihkZiRyYXRpbykKZGYgPC0gZGYgJT4lIGdyb3VwX2J5KHJhdGlvLCByb2ksIGdhaW4pICU+JSBzZWxlY3QoLVgxKQpgYGAKCiMgZ2VuZXJhdGUgYm9vdHN0cmFwcGVkIHZhbHVlcyBmb3IgbWVkaWFuIGFuZCBjaXMKYGBge3IgbWVzc2FnZT1GQUxTRSwgd2FybmluZz1GQUxTRSwgaW5jbHVkZT1GQUxTRX0KIyBydW4gaW4gYmF0Y2hlcyB0byByZWR1Y2UgbWVtb3J5IGZvb3RwcmludAppZiAoIWZpbGUuZXhpc3RzKG91dHB1dF9mbikpIHsKICBiYXRjaGVzIDwtIDUKICBuYnMgPC0gMTAwMAogIG5ic19iYXRjaCA8LSBuYnMgLyBiYXRjaGVzCiAgc2V0LnNlZWQoMTU0MjU0KQogIGRmX2JzIDwtIGxpc3QoKQogIGZvciAoYmF0Y2ggaW4gMTpiYXRjaGVzKSB7CiAgICBkZl9iYXRjaCA8LSBkZiAlPiUgZG8ocnM9bW9kZWxyOjpib290c3RyYXAoLiwgbmJzX2JhdGNoKSkgJT4lCiAgICAgIGdyb3VwX2J5KHJhdGlvLCByb2ksIGdhaW4pICU+JQogICAgICB1bm5lc3QoKSAlPiUKICAgICAgZ3JvdXBfYnkocmF0aW8sIHJvaSwgZ2FpbiwgLmlkKSAlPiUKICAgICAgZG8oYXMuZGF0YS5mcmFtZSguJHN0cmFwKSkgJT4lCiAgICAgIHN1bW1hcmlzZSh2YWx1ZT1tZWRpYW4odmFsdWUpKQogICAgZGZfYmF0Y2gkLmlkIDwtIGFzLm51bWVyaWMoZGZfYmF0Y2gkLmlkKSArIG5ic19iYXRjaCAqIChiYXRjaCAtIDEpCiAgICBkZl9icyA8LSByYmluZChkZl9icywgZGZfYmF0Y2gpCiAgfQogIAogICMgY29tcHV0ZSBjaXMKICBjaSA8LSA5NQogIGxfY2kgPC0gKDEwMCAtIGNpKS8yMDAKICBoX2NpIDwtIDEgLSBsX2NpCiAgY2lzIDwtCiAgZGZfYnMgJT4lCiAgICB1bmdyb3VwKCkgJT4lCiAgICBncm91cF9ieShyYXRpbywgcm9pLCBnYWluKSAlPiUKICAgIHN1bW1hcmlzZShsbz1xdWFudGlsZSh2YWx1ZSwgbF9jaSksCiAgICAgICAgICAgICAgaGk9cXVhbnRpbGUodmFsdWUsIGhfY2kpKQogIAogIGRmX3QwIDwtCiAgICBkZiAlPiUKICAgIGdyb3VwX2J5KHJhdGlvLCByb2ksIGdhaW4pICU+JQogICAgc3VtbWFyaXNlKHZhbHVlPW1lZGlhbih2YWx1ZSkpCiAgCiAgZGZfdDAgPC0KICBkZl90MCAlPiUKICAgIG1lcmdlKGNpcykKICAKICBkZl90MCRyb2kgPC0gZmFjdG9yKGRmX3QwJHJvaSwgbGV2ZWxzPWMoJ0lPRycsICdwRnVzJywgJ21GdXMnKSkKICB3cml0ZV9jc3YoZGZfdDAsIG91dHB1dF9mbikKfSBlbHNlIHsKICBkZl90MCA8LSByZWFkX2NzdihvdXRwdXRfZm4pICAKfQpgYGAKCmBgYHtyfQp0aGVtZV9QdWJsaWNhdGlvbiA8LSBmdW5jdGlvbihiYXNlX3NpemU9MTIpIHsKICAgICAgbGlicmFyeShnZ3RoZW1lcykKICAgICAgKHRoZW1lX2ZvdW5kYXRpb24oYmFzZV9zaXplPWJhc2Vfc2l6ZSkKICAgICAgICsgdGhlbWUocGxvdC50aXRsZSA9IGVsZW1lbnRfdGV4dChmYWNlID0gImJvbGQiLAogICAgICAgICAgICAgICAgICAgICAgICAgICAgICAgICAgICAgICAgIHNpemUgPSByZWwoMS4yKSwgaGp1c3QgPSAwLjUpLAogICAgICAgICAgICAgICB0ZXh0ID0gZWxlbWVudF90ZXh0KCksCiAgICAgICAgICAgICAgIHBhbmVsLmJhY2tncm91bmQgPSBlbGVtZW50X3JlY3QoY29sb3VyID0gTkEpLAogICAgICAgICAgICAgICBwbG90LmJhY2tncm91bmQgPSBlbGVtZW50X3JlY3QoY29sb3VyID0gTkEpLAogICAgICAgICAgICAgICBwYW5lbC5ib3JkZXIgPSBlbGVtZW50X3JlY3QoY29sb3VyID0gTkEpLAogICAgICAgICAgICAgICBheGlzLnRpdGxlID0gZWxlbWVudF90ZXh0KHNpemUgPSByZWwoMSkpLAogICAgICAgICAgICAgICBheGlzLnRpdGxlLnkgPSBlbGVtZW50X3RleHQoYW5nbGU9OTAsdmp1c3QgPTIpLAogICAgICAgICAgICAgICBheGlzLnRpdGxlLnggPSBlbGVtZW50X3RleHQodmp1c3QgPSAtMC4yKSwKICAgICAgICAgICAgICAgYXhpcy50ZXh0ID0gZWxlbWVudF90ZXh0KCksIAogICAgICAgICAgICAgICBheGlzLmxpbmUgPSBlbGVtZW50X2xpbmUoY29sb3VyPSJibGFjayIpLAogICAgICAgICAgICAgICBheGlzLnRpY2tzID0gZWxlbWVudF9saW5lKCksCiAgICAgICAgICAgICAgIHBhbmVsLmdyaWQubWFqb3IgPSBlbGVtZW50X2JsYW5rKCksICNlbGVtZW50X2xpbmUoY29sb3VyPSIjZjBmMGYwIiksCiAgICAgICAgICAgICAgIHBhbmVsLmdyaWQubWlub3IgPSBlbGVtZW50X2JsYW5rKCksCiAgICAgICAgICAgICAgIGxlZ2VuZC5rZXkgPSBlbGVtZW50X3JlY3QoY29sb3VyID0gTkEpLAogICAgICAgICAgICAgICBsZWdlbmQucG9zaXRpb24gPSAiYm90dG9tIiwKICAgICAgICAgICAgICAgbGVnZW5kLmRpcmVjdGlvbiA9ICJob3Jpem9udGFsIiwKICAgICAgICAgICAgICAgI2xlZ2VuZC5rZXkuc2l6ZT0gdW5pdCgwLjIsICJjbSIpLAogICAgICAgICAgICAgICBsZWdlbmQuc3BhY2luZyA9IHVuaXQoMCwgImNtIiksCiAgICAgICAgICAgICAgIGxlZ2VuZC50aXRsZSA9IGVsZW1lbnRfdGV4dCgpLAogICAgICAgICAgICAgICBwbG90Lm1hcmdpbiA9IHVuaXQoYygxMCw1LDUsNSksIm1tIiksCiAgICAgICAgICAgICAgIHN0cmlwLmJhY2tncm91bmQgPSBlbGVtZW50X3JlY3QoY29sb3VyPSIjZjBmMGYwIixmaWxsPSIjZjBmMGYwIiksCiAgICAgICAgICAgICAgIHN0cmlwLnRleHQgPSBlbGVtZW50X3RleHQoZmFjZT0iYm9sZCIpLAogICAgICAgICAgICAgICBzdHJpcC50ZXh0LnkgPSBlbGVtZW50X3RleHQoYW5nbGUgPSAwKQogICAgICAgICAgKSkKICAgICAgCn0KCmBgYAoKYGBge3J9CmRmX3QwX2dhaW4xIDwtIGRmX3QwICU+JQogIGZpbHRlcihnYWluID09IDEuMCkKCmlmICh0YXNrID09ICdlc3RpbWF0aW9uJykgewogIHlfbGltcyA8LSBjKDAsIDEuMjUpCn0gZWxzZSB7CiAgeV9saW1zIDwtIGMoMCwgMC44KQp9CmBgYApgYGB7ciBmaWcuaGVpZ2h0PTMsIGZpZy53aWR0aD00fQpkZl90MF9nYWluMSAlPiUKICBnZ3Bsb3QoYWVzKHJhdGlvLCB2YWx1ZSwgeW1pbj1sbywgeW1heD1oaSwgY29sb3I9cm9pLCBncm91cD1yb2kpKSArCiAgZ2VvbV9saW5lcmFuZ2Uoc2hvdy5sZWdlbmQ9RikgKwogIGdlb21fbGluZShzaG93LmxlZ2VuZD1GKSArCiAgZ2VvbV9wb2ludChzaXplPTEuNSkgKwogIGxhYnMoeD0iUmF0aW8gb2YgdW5pdHMgc2VsZWN0aXZlIHRvIGlkZW50aXR5IGEiLAogICAgICAgeT0iU2ltdWxhdGVkIFBTRSB2YXJpYW5jZSIsCiAgICAgICBjb2xvcj0iIikgKwogIHRoZW1lX1B1YmxpY2F0aW9uKCkgKwogIHRoZW1lKGxlZ2VuZC5kaXJlY3Rpb249J3ZlcnRpY2FsJywKICAgICAgICBsZWdlbmQucG9zaXRpb249YygwLjgsIDAuOCkpICsKICBzY2FsZV9jb2xvcl9icmV3ZXIocGFsZXR0ZT0nU2V0MScpICsKICBjb29yZF9jYXJ0ZXNpYW4oeWxpbT15X2xpbXMpCgppbWcgPC0gcGFzdGUoaW1nZm4sICdfZGF0YS5wbmcnLCBzZXA9JycpCmdnc2F2ZShpbWcsIGRwaT0zMDAsIGhlaWdodD0zLCB3aWR0aD00KQpgYGAKYGBge3J9CiMgbWFrZSBteSBvd24gcGFsZXR0ZXMgdG8gYXZvaWQgdG9vIGxpZ2h0IGNvbG9ycwpsaWJyYXJ5KFJDb2xvckJyZXdlcikKCnJlZCA8LSBicmV3ZXIucGFsKG49OSwgIlJlZHMiKQpyZWRfcGFsZXR0ZSA8LSBjb2xvclJhbXBQYWxldHRlKGMocmVkWzNdLCByZWRbNl0sIHJlZFs5XSksIHNwYWNlID0gIkxhYiIpCm15X3JlZCA8LSByZWRfcGFsZXR0ZSg3KQpibHVlIDwtIGJyZXdlci5wYWwobj05LCAiQmx1ZXMiKQpibHVlX3BhbGV0dGUgPC0gY29sb3JSYW1wUGFsZXR0ZShjKGJsdWVbM10sIGJsdWVbNl0sIGJsdWVbOV0pLCBzcGFjZSA9ICJMYWIiKQpteV9ibHVlIDwtIGJsdWVfcGFsZXR0ZSg3KQpncmVlbiA8LSBicmV3ZXIucGFsKG49OSwgIkdyZWVucyIpCmdyZWVuX3BhbGV0dGUgPC0gY29sb3JSYW1wUGFsZXR0ZShjKGdyZWVuWzNdLCBncmVlbls2XSwgZ3JlZW5bOV0pLCBzcGFjZSA9ICJMYWIiKQpteV9ncmVlbiA8LSBncmVlbl9wYWxldHRlKDcpCmBgYApgYGB7ciBmaWcuaGVpZ2h0PTIuMjUsIGZpZy53aWR0aD0zfQpkZl90MF9pb2cgPC0gZmlsdGVyKGRmX3QwLCByb2kgPT0gJ0lPRycpCmRmX3QwX2lvZyRnYWluIDwtIGFzLmZhY3RvcihkZl90MF9pb2ckZ2FpbikKCmRmX3QwX2lvZyAlPiUKICBnZ3Bsb3QoYWVzKHJhdGlvLCB2YWx1ZSwgY29sb3I9Z2FpbiwgZ3JvdXA9Z2FpbikpICsKICBnZW9tX2xpbmUoc2hvdy5sZWdlbmQ9RikgKwogIGdlb21fcG9pbnQoc2l6ZT0xLjUpICsKICBsYWJzKHg9IlJhdGlvIG9mIHVuaXRzIHNlbGVjdGl2ZSB0byBpZGVudGl0eSBhIiwKICAgICAgIHk9IlNpbXVsYXRlZCBQU0UgdmFyaWFuY2UiLAogICAgICAgY29sb3I9IkdhaW4gYS9iIikgKwogIHRoZW1lX1B1YmxpY2F0aW9uKGJhc2Vfc2l6ZT0xMCkgKwogIHRoZW1lKGxlZ2VuZC5kaXJlY3Rpb249J3ZlcnRpY2FsJywKICAgICAgICBsZWdlbmQucG9zaXRpb249YygwLjksIDAuNjUpLAogICAgICAgIGxlZ2VuZC50aXRsZT1lbGVtZW50X3RleHQoc2l6ZT0xMCksCiAgICAgICAgbGVnZW5kLmtleS5zaXplPXVuaXQoMTAsICdwdCcpLAogICAgICAgIGxlZ2VuZC50ZXh0PWVsZW1lbnRfdGV4dChzaXplPTgpKSArIAogIHNjYWxlX2NvbG9yX21hbnVhbCh2YWx1ZXM9bXlfcmVkKSArCiAgY29vcmRfY2FydGVzaWFuKHlsaW09eV9saW1zKQoKaW1nIDwtIHBhc3RlKGltZ2ZuLCAnX2luY3JlYXNlLWdhaW5faW9nLnBuZycsIHNlcD0nJykKZ2dzYXZlKGltZywgZHBpPTMwMCwgaGVpZ2h0PTIuMjUsIHdpZHRoPTMuKQpgYGAKYGBge3IgZmlnLmhlaWdodD0yLjI1LCBmaWcud2lkdGg9M30KZGZfdDBfcGZ1cyA8LSBmaWx0ZXIoZGZfdDAsIHJvaSA9PSAncEZ1cycpCmRmX3QwX3BmdXMkZ2FpbiA8LSBhcy5mYWN0b3IoZGZfdDBfcGZ1cyRnYWluKQoKZGZfdDBfcGZ1cyAlPiUKICBnZ3Bsb3QoYWVzKHJhdGlvLCB2YWx1ZSwgY29sb3I9Z2FpbiwgZ3JvdXA9Z2FpbikpICsKICBnZW9tX2xpbmUoc2hvdy5sZWdlbmQ9RikgKwogIGdlb21fcG9pbnQoc2l6ZT0xLjUpICsKICBsYWJzKHg9IlJhdGlvIG9mIHVuaXRzIHNlbGVjdGl2ZSB0byBpZGVudGl0eSBhIiwKICAgICAgIHk9IlNpbXVsYXRlZCBQU0UgdmFyaWFuY2UiLAogICAgICAgY29sb3I9IkdhaW4gYS9iIikgKwogIHRoZW1lX1B1YmxpY2F0aW9uKGJhc2Vfc2l6ZT0xMCkgKwogIHRoZW1lKGxlZ2VuZC5kaXJlY3Rpb249J3ZlcnRpY2FsJywKICAgICAgICBsZWdlbmQucG9zaXRpb249YygwLjksIDAuNjUpLAogICAgICAgIGxlZ2VuZC50aXRsZT1lbGVtZW50X3RleHQoc2l6ZT0xMCksCiAgICAgICAgbGVnZW5kLmtleS5zaXplPXVuaXQoMTAsICdwdCcpLAogICAgICAgIGxlZ2VuZC50ZXh0PWVsZW1lbnRfdGV4dChzaXplPTgpKSArIAogIHNjYWxlX2NvbG9yX21hbnVhbCh2YWx1ZXM9bXlfYmx1ZSkgKwogIGNvb3JkX2NhcnRlc2lhbih5bGltPXlfbGltcykKCmltZyA8LSBwYXN0ZShpbWdmbiwgJ19pbmNyZWFzZS1nYWluX3BmdXMucG5nJywgc2VwPScnKQpnZ3NhdmUoaW1nLCBkcGk9MzAwLCBoZWlnaHQ9Mi4yNSwgd2lkdGg9My4pCmBgYAoKYGBge3IgZmlnLmhlaWdodD0yLjI1LCBmaWcud2lkdGg9M30KZGZfdDBfbWZ1cyA8LSBmaWx0ZXIoZGZfdDAsIHJvaSA9PSAnbUZ1cycpCmRmX3QwX21mdXMkZ2FpbiA8LSBhcy5mYWN0b3IoZGZfdDBfbWZ1cyRnYWluKQoKZGZfdDBfbWZ1cyAlPiUKICBnZ3Bsb3QoYWVzKHJhdGlvLCB2YWx1ZSwgY29sb3I9Z2FpbiwgZ3JvdXA9Z2FpbikpICsKICBnZW9tX2xpbmUoc2hvdy5sZWdlbmQ9RikgKwogIGdlb21fcG9pbnQoc2l6ZT0xLjUpICsKICBsYWJzKHg9IlJhdGlvIG9mIHVuaXRzIHNlbGVjdGl2ZSB0byBpZGVudGl0eSBhIiwKICAgICAgIHk9IlNpbXVsYXRlZCBQU0UgdmFyaWFuY2UiLAogICAgICAgY29sb3I9IkdhaW4gYS9iIikgKwogIHRoZW1lX1B1YmxpY2F0aW9uKGJhc2Vfc2l6ZT0xMCkgKwogIHRoZW1lKGxlZ2VuZC5kaXJlY3Rpb249J3ZlcnRpY2FsJywKICAgICAgICBsZWdlbmQucG9zaXRpb249YygwLjksIDAuNjUpLAogICAgICAgIGxlZ2VuZC50aXRsZT1lbGVtZW50X3RleHQoc2l6ZT0xMCksCiAgICAgICAgbGVnZW5kLmtleS5zaXplPXVuaXQoMTAsICdwdCcpLAogICAgICAgIGxlZ2VuZC50ZXh0PWVsZW1lbnRfdGV4dChzaXplPTgpKSArIAogIHNjYWxlX2NvbG9yX21hbnVhbCh2YWx1ZXM9bXlfZ3JlZW4pICsKICBjb29yZF9jYXJ0ZXNpYW4oeWxpbT15X2xpbXMpCgppbWcgPC0gcGFzdGUoaW1nZm4sICdfaW5jcmVhc2UtZ2Fpbl9tZnVzLnBuZycsIHNlcD0nJykKZ2dzYXZlKGltZywgZHBpPTMwMCwgaGVpZ2h0PTIuMjUsIHdpZHRoPTMuKQpgYGAK
