# Supplementary material for: Idiosyncratic, Retinotopic Bias in Face Identification Modulated by Familiarity
Source: eNeuro. 2018 Oct 4;5(5):ENEURO.0054-18.2018. doi: 10.1523/ENEURO.0054-18.2018 (PMC6171739; doi:10.1523/ENEURO.0054-18.2018)
Supplement: Extended Data — The archive contains data from both experiments, as well as the analysis scripts. Download Extended Data 1, ZIP file. [file sup_enu-eN-NWR-0054-18-s02.zip › famretino2-3.0.0/model/plot_simdata_moreunits.nb.html]

Plotting modeling results


Code 

- Show All Code
- Hide All Code
- Download Rmd

# Plotting modeling results


```
require(tidyverse)
```


```
# task <- 'estimation'  # or 'face'
task <- 'face'  # or 'face'
output_fn <- paste('outputs/sim-05_task-', task, '_cis.csv', sep='')
imgfn <- paste('img/sim-05_task-', task, sep='')
input_fn <-  paste('outputs/sim-05_task-', task, 
        '_increase_units_increase_voxels_500sim.csv', sep='')
df <- read_csv(input_fn)
```


```
Missing column names filled in: 'X1' [1]Parsed with column specification:
cols(
  X1 = col_integer(),
  roi = col_character(),
  ratio = col_integer(),
  n_voxels = col_integer(),
  gain = col_double(),
  rf_increase = col_double(),
  value = col_double(),
  b_units = col_integer()
)
```


```
df$ratio <- as.factor(df$ratio)
df$b_units <- as.factor(df$b_units)
df <- df %>% group_by(ratio, roi, b_units) %>% select(-X1)
```


# generate bootstrapped values for median and cis


```
theme_Publication <- function(base_size=12) {
      library(ggthemes)
      (theme_foundation(base_size=base_size)
       + theme(plot.title = element_text(face = "bold",
                                         size = rel(1.2), hjust = 0.5),
               text = element_text(),
               panel.background = element_rect(colour = NA),
               plot.background = element_rect(colour = NA),
               panel.border = element_rect(colour = NA),
               axis.title = element_text(size = rel(1)),
               axis.title.y = element_text(angle=90,vjust =2),
               axis.title.x = element_text(vjust = -0.2),
               axis.text = element_text(), 
               axis.line = element_line(colour="black"),
               axis.ticks = element_line(),
               panel.grid.major = element_blank(), #element_line(colour="#f0f0f0"),
               panel.grid.minor = element_blank(),
               legend.key = element_rect(colour = NA),
               legend.position = "bottom",
               legend.direction = "horizontal",
               #legend.key.size= unit(0.2, "cm"),
               legend.spacing = unit(0, "cm"),
               legend.title = element_text(),
               plot.margin = unit(c(10,5,5,5),"mm"),
               strip.background = element_rect(colour="#f0f0f0",fill="#f0f0f0"),
               strip.text = element_text(face="bold"),
               strip.text.y = element_text(angle = 0)
          ))
      
}
```


```
for (bu in 1:5) {
  if (bu == 1) {
    title <- "unit responsive to b"
  } else {
    title <- "units responsive to b"
  }
  
  df_t0 %>%
    filter(b_units == bu) %>%
    ggplot(aes(ratio, value, ymin=lo, ymax=hi, color=roi, group=roi)) +
    geom_linerange(show.legend=F) +
    geom_line(show.legend=F) +
    geom_point(size=1.5) +
    labs(x="Ratio of units selective to identity a",
         y="Simulated PSE variance",
         color="") +
    ggtitle(paste(bu, title)) +
    theme_Publication() +
    theme(legend.direction='vertical',
          legend.position=c(0.8, 0.8),
          plot.title=element_text(size=12, face='bold')) +
    scale_color_brewer(palette='Set1')
    # coord_cartesian(ylim=y_lims) 
  
  img <- paste(imgfn, '_data_', bu, 'units.png', sep='')
  ggsave(img, dpi=300, height=3.2, width=4)
}
```


```
package ‘ggthemes’ was built under R version 3.2.5
```

LS0tCnRpdGxlOiAiUGxvdHRpbmcgbW9kZWxpbmcgcmVzdWx0cyIKb3V0cHV0OiBodG1sX25vdGVib29rCi0tLQpgYGB7ciBtZXNzYWdlPUZBTFNFLCB3YXJuaW5nPUZBTFNFfQpyZXF1aXJlKHRpZHl2ZXJzZSkKYGBgCgpgYGB7cn0KIyB0YXNrIDwtICdlc3RpbWF0aW9uJyAgIyBvciAnZmFjZScKdGFzayA8LSAnZmFjZScgICMgb3IgJ2ZhY2UnCm91dHB1dF9mbiA8LSBwYXN0ZSgnb3V0cHV0cy9zaW0tMDVfdGFzay0nLCB0YXNrLCAnX2Npcy5jc3YnLCBzZXA9JycpCmltZ2ZuIDwtIHBhc3RlKCdpbWcvc2ltLTA1X3Rhc2stJywgdGFzaywgc2VwPScnKQppbnB1dF9mbiA8LSAgcGFzdGUoJ291dHB1dHMvc2ltLTA1X3Rhc2stJywgdGFzaywgCiAgICAgICAgJ19pbmNyZWFzZV91bml0c19pbmNyZWFzZV92b3hlbHNfNTAwc2ltLmNzdicsIHNlcD0nJykKZGYgPC0gcmVhZF9jc3YoaW5wdXRfZm4pCmRmJHJhdGlvIDwtIGFzLmZhY3RvcihkZiRyYXRpbykKZGYkYl91bml0cyA8LSBhcy5mYWN0b3IoZGYkYl91bml0cykKZGYgPC0gZGYgJT4lIGdyb3VwX2J5KHJhdGlvLCByb2ksIGJfdW5pdHMpICU+JSBzZWxlY3QoLVgxKQpgYGAKCiMgZ2VuZXJhdGUgYm9vdHN0cmFwcGVkIHZhbHVlcyBmb3IgbWVkaWFuIGFuZCBjaXMKYGBge3IgbWVzc2FnZT1GQUxTRSwgd2FybmluZz1GQUxTRSwgaW5jbHVkZT1GQUxTRX0KIyBydW4gaW4gYmF0Y2hlcyB0byByZWR1Y2UgbWVtb3J5IGZvb3RwcmludAppZiAoIWZpbGUuZXhpc3RzKG91dHB1dF9mbikpIHsKICBiYXRjaGVzIDwtIDUKICBuYnMgPC0gMTAwMAogIG5ic19iYXRjaCA8LSBuYnMgLyBiYXRjaGVzCiAgc2V0LnNlZWQoMTU0MjU0KQogIGRmX2JzIDwtIGxpc3QoKQogIGZvciAoYmF0Y2ggaW4gMTpiYXRjaGVzKSB7CiAgICBkZl9iYXRjaCA8LSBkZiAlPiUgZG8ocnM9bW9kZWxyOjpib290c3RyYXAoLiwgbmJzX2JhdGNoKSkgJT4lCiAgICAgIGdyb3VwX2J5KHJhdGlvLCByb2ksIGJfdW5pdHMpICU+JQogICAgICB1bm5lc3QoKSAlPiUKICAgICAgZ3JvdXBfYnkocmF0aW8sIHJvaSwgYl91bml0cywgLmlkKSAlPiUKICAgICAgZG8oYXMuZGF0YS5mcmFtZSguJHN0cmFwKSkgJT4lCiAgICAgIHN1bW1hcmlzZSh2YWx1ZT1tZWRpYW4odmFsdWUpKQogICAgZGZfYmF0Y2gkLmlkIDwtIGFzLm51bWVyaWMoZGZfYmF0Y2gkLmlkKSArIG5ic19iYXRjaCAqIChiYXRjaCAtIDEpCiAgICBkZl9icyA8LSByYmluZChkZl9icywgZGZfYmF0Y2gpCiAgfQogIAogICMgY29tcHV0ZSBjaXMKICBjaSA8LSA5NQogIGxfY2kgPC0gKDEwMCAtIGNpKS8yMDAKICBoX2NpIDwtIDEgLSBsX2NpCiAgY2lzIDwtCiAgZGZfYnMgJT4lCiAgICB1bmdyb3VwKCkgJT4lCiAgICBncm91cF9ieShyYXRpbywgcm9pLCBiX3VuaXRzKSAlPiUKICAgIHN1bW1hcmlzZShsbz1xdWFudGlsZSh2YWx1ZSwgbF9jaSksCiAgICAgICAgICAgICAgaGk9cXVhbnRpbGUodmFsdWUsIGhfY2kpKQogIAogIGRmX3QwIDwtCiAgICBkZiAlPiUKICAgIGdyb3VwX2J5KHJhdGlvLCByb2ksIGJfdW5pdHMpICU+JQogICAgc3VtbWFyaXNlKHZhbHVlPW1lZGlhbih2YWx1ZSkpCiAgCiAgZGZfdDAgPC0KICBkZl90MCAlPiUKICAgIG1lcmdlKGNpcykKICAKICBkZl90MCRyb2kgPC0gZmFjdG9yKGRmX3QwJHJvaSwgbGV2ZWxzPWMoJ0lPRycsICdwRnVzJywgJ21GdXMnKSkKICB3cml0ZV9jc3YoZGZfdDAsIG91dHB1dF9mbikKfSBlbHNlIHsKICBkZl90MCA8LSByZWFkX2NzdihvdXRwdXRfZm4pICAKfQpgYGAKCmBgYHtyfQp0aGVtZV9QdWJsaWNhdGlvbiA8LSBmdW5jdGlvbihiYXNlX3NpemU9MTIpIHsKICAgICAgbGlicmFyeShnZ3RoZW1lcykKICAgICAgKHRoZW1lX2ZvdW5kYXRpb24oYmFzZV9zaXplPWJhc2Vfc2l6ZSkKICAgICAgICsgdGhlbWUocGxvdC50aXRsZSA9IGVsZW1lbnRfdGV4dChmYWNlID0gImJvbGQiLAogICAgICAgICAgICAgICAgICAgICAgICAgICAgICAgICAgICAgICAgIHNpemUgPSByZWwoMS4yKSwgaGp1c3QgPSAwLjUpLAogICAgICAgICAgICAgICB0ZXh0ID0gZWxlbWVudF90ZXh0KCksCiAgICAgICAgICAgICAgIHBhbmVsLmJhY2tncm91bmQgPSBlbGVtZW50X3JlY3QoY29sb3VyID0gTkEpLAogICAgICAgICAgICAgICBwbG90LmJhY2tncm91bmQgPSBlbGVtZW50X3JlY3QoY29sb3VyID0gTkEpLAogICAgICAgICAgICAgICBwYW5lbC5ib3JkZXIgPSBlbGVtZW50X3JlY3QoY29sb3VyID0gTkEpLAogICAgICAgICAgICAgICBheGlzLnRpdGxlID0gZWxlbWVudF90ZXh0KHNpemUgPSByZWwoMSkpLAogICAgICAgICAgICAgICBheGlzLnRpdGxlLnkgPSBlbGVtZW50X3RleHQoYW5nbGU9OTAsdmp1c3QgPTIpLAogICAgICAgICAgICAgICBheGlzLnRpdGxlLnggPSBlbGVtZW50X3RleHQodmp1c3QgPSAtMC4yKSwKICAgICAgICAgICAgICAgYXhpcy50ZXh0ID0gZWxlbWVudF90ZXh0KCksIAogICAgICAgICAgICAgICBheGlzLmxpbmUgPSBlbGVtZW50X2xpbmUoY29sb3VyPSJibGFjayIpLAogICAgICAgICAgICAgICBheGlzLnRpY2tzID0gZWxlbWVudF9saW5lKCksCiAgICAgICAgICAgICAgIHBhbmVsLmdyaWQubWFqb3IgPSBlbGVtZW50X2JsYW5rKCksICNlbGVtZW50X2xpbmUoY29sb3VyPSIjZjBmMGYwIiksCiAgICAgICAgICAgICAgIHBhbmVsLmdyaWQubWlub3IgPSBlbGVtZW50X2JsYW5rKCksCiAgICAgICAgICAgICAgIGxlZ2VuZC5rZXkgPSBlbGVtZW50X3JlY3QoY29sb3VyID0gTkEpLAogICAgICAgICAgICAgICBsZWdlbmQucG9zaXRpb24gPSAiYm90dG9tIiwKICAgICAgICAgICAgICAgbGVnZW5kLmRpcmVjdGlvbiA9ICJob3Jpem9udGFsIiwKICAgICAgICAgICAgICAgI2xlZ2VuZC5rZXkuc2l6ZT0gdW5pdCgwLjIsICJjbSIpLAogICAgICAgICAgICAgICBsZWdlbmQuc3BhY2luZyA9IHVuaXQoMCwgImNtIiksCiAgICAgICAgICAgICAgIGxlZ2VuZC50aXRsZSA9IGVsZW1lbnRfdGV4dCgpLAogICAgICAgICAgICAgICBwbG90Lm1hcmdpbiA9IHVuaXQoYygxMCw1LDUsNSksIm1tIiksCiAgICAgICAgICAgICAgIHN0cmlwLmJhY2tncm91bmQgPSBlbGVtZW50X3JlY3QoY29sb3VyPSIjZjBmMGYwIixmaWxsPSIjZjBmMGYwIiksCiAgICAgICAgICAgICAgIHN0cmlwLnRleHQgPSBlbGVtZW50X3RleHQoZmFjZT0iYm9sZCIpLAogICAgICAgICAgICAgICBzdHJpcC50ZXh0LnkgPSBlbGVtZW50X3RleHQoYW5nbGUgPSAwKQogICAgICAgICAgKSkKICAgICAgCn0KCmBgYAoKYGBge3IgZmlnLmhlaWdodD0zLjIsIGZpZy53aWR0aD00fQpmb3IgKGJ1IGluIDE6NSkgewogIGlmIChidSA9PSAxKSB7CiAgICB0aXRsZSA8LSAidW5pdCByZXNwb25zaXZlIHRvIGIiCiAgfSBlbHNlIHsKICAgIHRpdGxlIDwtICJ1bml0cyByZXNwb25zaXZlIHRvIGIiCiAgfQogIAogIGRmX3QwICU+JQogICAgZmlsdGVyKGJfdW5pdHMgPT0gYnUpICU+JQogICAgZ2dwbG90KGFlcyhyYXRpbywgdmFsdWUsIHltaW49bG8sIHltYXg9aGksIGNvbG9yPXJvaSwgZ3JvdXA9cm9pKSkgKwogICAgZ2VvbV9saW5lcmFuZ2Uoc2hvdy5sZWdlbmQ9RikgKwogICAgZ2VvbV9saW5lKHNob3cubGVnZW5kPUYpICsKICAgIGdlb21fcG9pbnQoc2l6ZT0xLjUpICsKICAgIGxhYnMoeD0iUmF0aW8gb2YgdW5pdHMgc2VsZWN0aXZlIHRvIGlkZW50aXR5IGEiLAogICAgICAgICB5PSJTaW11bGF0ZWQgUFNFIHZhcmlhbmNlIiwKICAgICAgICAgY29sb3I9IiIpICsKICAgIGdndGl0bGUocGFzdGUoYnUsIHRpdGxlKSkgKwogICAgdGhlbWVfUHVibGljYXRpb24oKSArCiAgICB0aGVtZShsZWdlbmQuZGlyZWN0aW9uPSd2ZXJ0aWNhbCcsCiAgICAgICAgICBsZWdlbmQucG9zaXRpb249YygwLjgsIDAuOCksCiAgICAgICAgICBwbG90LnRpdGxlPWVsZW1lbnRfdGV4dChzaXplPTEyLCBmYWNlPSdib2xkJykpICsKICAgIHNjYWxlX2NvbG9yX2JyZXdlcihwYWxldHRlPSdTZXQxJykKICAgICMgY29vcmRfY2FydGVzaWFuKHlsaW09eV9saW1zKSAKICAKICBpbWcgPC0gcGFzdGUoaW1nZm4sICdfZGF0YV8nLCBidSwgJ3VuaXRzLnBuZycsIHNlcD0nJykKICBnZ3NhdmUoaW1nLCBkcGk9MzAwLCBoZWlnaHQ9My4yLCB3aWR0aD00KQp9CmBgYAo=
