# Supplementary material for: Idiosyncratic, Retinotopic Bias in Face Identification Modulated by Familiarity
Source: eNeuro. 2018 Oct 4;5(5):ENEURO.0054-18.2018. doi: 10.1523/ENEURO.0054-18.2018 (PMC6171739; doi:10.1523/ENEURO.0054-18.2018)
Supplement: Extended Data — The archive contains data from both experiments, as well as the analysis scripts. Download Extended Data 1, ZIP file. [file sup_enu-eN-NWR-0054-18-s02.zip › famretino2-3.0.0/model/plot_simdata_rfincrease.nb.html]

Plotting modeling results - increase in RF size


Code 

- Show All Code
- Hide All Code
- Download Rmd

# Plotting modeling results - increase in RF size


```
require(tidyverse)
```


```
# task <- 'estimation'  # or 'face'
task <- 'face' 
output_fn <- paste('outputs/sim-04_task-', task, '_cis.csv', sep='')
imgfn <- paste('img/sim-04_task-', task, sep='')
input_fn <-  paste('outputs/sim-04_task-', task, 
        '_increase_units_increase_rfsize_500sim.csv', sep='')
df <- read_csv(input_fn)
```


```
Missing column names filled in: 'X1' [1]Parsed with column specification:
cols(
  X1 = col_integer(),
  roi = col_character(),
  ratio = col_integer(),
  n_voxels = col_integer(),
  gain = col_double(),
  rf_increase = col_double(),
  value = col_double(),
  b_units = col_integer()
)
```


```
df$ratio <- as.factor(df$ratio)
df$rf_increase <- as.factor(df$rf_increase)
df <- df %>% group_by(ratio, roi, rf_increase) %>% select(-X1)
```


# generate bootstrapped values for median and cis


```
theme_Publication <- function(base_size=12) {
      library(ggthemes)
      (theme_foundation(base_size=base_size)
       + theme(plot.title = element_text(face = "bold",
                                         size = rel(1.2), hjust = 0.5),
               text = element_text(),
               panel.background = element_rect(colour = NA),
               plot.background = element_rect(colour = NA),
               panel.border = element_rect(colour = NA),
               axis.title = element_text(size = rel(1)),
               axis.title.y = element_text(angle=90,vjust =2),
               axis.title.x = element_text(vjust = -0.2),
               axis.text = element_text(), 
               axis.line = element_line(colour="black"),
               axis.ticks = element_line(),
               panel.grid.major = element_blank(), #element_line(colour="#f0f0f0"),
               panel.grid.minor = element_blank(),
               legend.key = element_rect(colour = NA),
               legend.position = "bottom",
               legend.direction = "horizontal",
               #legend.key.size= unit(0.2, "cm"),
               legend.spacing = unit(0, "cm"),
               legend.title = element_text(),
               plot.margin = unit(c(10,5,5,5),"mm"),
               strip.background = element_rect(colour="#f0f0f0",fill="#f0f0f0"),
               strip.text = element_text(face="bold"),
               strip.text.y = element_text(angle = 0)
          ))
      
}
```


```
df_t0_rfi0 <- df_t0 %>%
  filter(rf_increase == 0)
if (task == 'estimation') {
  y_lims <- c(0, 1.25)
} else {
  y_lims <- c(0, 0.8)
}
```


```
df_t0_rfi0 %>%
  ggplot(aes(ratio, value, ymin=lo, ymax=hi, color=roi, group=roi)) +
  geom_linerange(show.legend=F) +
  geom_line(show.legend=F) +
  geom_point(size=1.5) +
  labs(x="Ratio of units selective to identity a",
       y="Simulated PSE variance",
       color="") +
  theme_Publication() +
  theme(legend.direction='vertical',
        legend.position=c(0.8, 0.8)) +
  scale_color_brewer(palette='Set1') +
  coord_cartesian(ylim=y_lims)
```


```
#img <- paste(imgfn, '_data.png', sep='')
#ggsave(img, dpi=300, height=3, width=4)
```


```
# make my own palettes to avoid too light colors
library(RColorBrewer)
red <- brewer.pal(n=9, "Reds")
red_palette <- colorRampPalette(c(red[3], red[6], red[9]), space = "Lab")
my_red <- red_palette(7)
blue <- brewer.pal(n=9, "Blues")
blue_palette <- colorRampPalette(c(blue[3], blue[6], blue[9]), space = "Lab")
my_blue <- blue_palette(7)
green <- brewer.pal(n=9, "Greens")
green_palette <- colorRampPalette(c(green[3], green[6], green[9]), space = "Lab")
my_green <- green_palette(7)
```


```
df_t0_iog <- filter(df_t0, roi == 'IOG')
df_t0_iog$rf_increase <- as.numeric(as.character(df_t0_iog$rf_increase)) * 100
df_t0_iog$rf_increase <- as.factor(df_t0_iog$rf_increase)
df_t0_iog %>%
  ggplot(aes(ratio, value, color=rf_increase, group=rf_increase)) +
  geom_line(show.legend=F) +
  geom_point(size=1.5) +
  labs(x="Ratio of units selective to identity a",
       y="Simulated PSE variance",
       color="RF increase [%]") +
  theme_Publication(base_size=10) +
  theme(legend.direction='vertical',
        legend.position=c(0.8, 0.8),
        legend.title=element_text(size=10),
        legend.key.size=unit(10, 'pt'),
        legend.text=element_text(size=8)) + 
  scale_color_manual(values=my_red) +
  coord_cartesian(ylim=y_lims)
```


```
img <- paste(imgfn, '_increase-rfsize_iog.png', sep='')
ggsave(img, dpi=300, height=2.25, width=3.)
```


```
df_t0_pfus <- filter(df_t0, roi == 'pFus')
df_t0_pfus$rf_increase <- as.numeric(as.character(df_t0_pfus$rf_increase)) * 100
df_t0_pfus$rf_increase <- as.factor(df_t0_pfus$rf_increase)
df_t0_pfus %>%
  ggplot(aes(ratio, value, color=rf_increase, group=rf_increase)) +
  geom_line(show.legend=F) +
  geom_point(size=1.5) +
  labs(x="Ratio of units selective to identity a",
       y="Simulated PSE variance",
       color="RF increase [%]") +
  theme_Publication(base_size=10) +
  theme(legend.direction='vertical',
        legend.position=c(0.8, 0.8),
        legend.title=element_text(size=10),
        legend.key.size=unit(10, 'pt'),
        legend.text=element_text(size=8)) + 
  scale_color_manual(values=my_blue) +
  coord_cartesian(ylim=y_lims)
```


```
img <- paste(imgfn, '_increase-rfsize_pfus.png', sep='')
ggsave(img, dpi=300, height=2.25, width=3.)
```


```
df_t0_mfus <- filter(df_t0, roi == 'mFus')
df_t0_mfus$rf_increase <- as.numeric(as.character(df_t0_mfus$rf_increase)) * 100
df_t0_mfus$rf_increase <- as.factor(df_t0_mfus$rf_increase)
df_t0_mfus %>%
  ggplot(aes(ratio, value, color=rf_increase, group=rf_increase)) +
  geom_line(show.legend=F) +
  geom_point(size=1.5) +
  labs(x="Ratio of units selective to identity a",
       y="Simulated PSE variance",
       color="RF increase [%]") +
  theme_Publication(base_size=10) +
  theme(legend.direction='vertical',
        legend.position=c(0.8, 0.8),
        legend.title=element_text(size=10),
        legend.key.size=unit(10, 'pt'),
        legend.text=element_text(size=8)) + 
  scale_color_manual(values=my_green) +
  coord_cartesian(ylim=y_lims)
```


```
img <- paste(imgfn, '_increase-rfsize_mfus.png', sep='')
ggsave(img, dpi=300, height=2.25, width=3.)
```

LS0tCnRpdGxlOiAiUGxvdHRpbmcgbW9kZWxpbmcgcmVzdWx0cyAtIGluY3JlYXNlIGluIFJGIHNpemUiCm91dHB1dDogaHRtbF9ub3RlYm9vawotLS0KYGBge3IgbWVzc2FnZT1GQUxTRSwgd2FybmluZz1GQUxTRX0KcmVxdWlyZSh0aWR5dmVyc2UpCmBgYAoKYGBge3J9CiMgdGFzayA8LSAnZXN0aW1hdGlvbicgICMgb3IgJ2ZhY2UnCnRhc2sgPC0gJ2ZhY2UnIApvdXRwdXRfZm4gPC0gcGFzdGUoJ291dHB1dHMvc2ltLTA0X3Rhc2stJywgdGFzaywgJ19jaXMuY3N2Jywgc2VwPScnKQppbWdmbiA8LSBwYXN0ZSgnaW1nL3NpbS0wNF90YXNrLScsIHRhc2ssIHNlcD0nJykKaW5wdXRfZm4gPC0gIHBhc3RlKCdvdXRwdXRzL3NpbS0wNF90YXNrLScsIHRhc2ssIAogICAgICAgICdfaW5jcmVhc2VfdW5pdHNfaW5jcmVhc2VfcmZzaXplXzUwMHNpbS5jc3YnLCBzZXA9JycpCmRmIDwtIHJlYWRfY3N2KGlucHV0X2ZuKQpkZiRyYXRpbyA8LSBhcy5mYWN0b3IoZGYkcmF0aW8pCmRmJHJmX2luY3JlYXNlIDwtIGFzLmZhY3RvcihkZiRyZl9pbmNyZWFzZSkKZGYgPC0gZGYgJT4lIGdyb3VwX2J5KHJhdGlvLCByb2ksIHJmX2luY3JlYXNlKSAlPiUgc2VsZWN0KC1YMSkKYGBgCgojIGdlbmVyYXRlIGJvb3RzdHJhcHBlZCB2YWx1ZXMgZm9yIG1lZGlhbiBhbmQgY2lzCmBgYHtyIG1lc3NhZ2U9RkFMU0UsIHdhcm5pbmc9RkFMU0UsIGluY2x1ZGU9RkFMU0V9CiMgcnVuIGluIGJhdGNoZXMgdG8gcmVkdWNlIG1lbW9yeSBmb290cHJpbnQKaWYgKCFmaWxlLmV4aXN0cyhvdXRwdXRfZm4pKSB7CiAgYmF0Y2hlcyA8LSA1CiAgbmJzIDwtIDEwMDAKICBuYnNfYmF0Y2ggPC0gbmJzIC8gYmF0Y2hlcwogIHNldC5zZWVkKDE1NDI1NCkKICBkZl9icyA8LSBsaXN0KCkKICBmb3IgKGJhdGNoIGluIDE6YmF0Y2hlcykgewogICAgZGZfYmF0Y2ggPC0gZGYgJT4lIGRvKHJzPW1vZGVscjo6Ym9vdHN0cmFwKC4sIG5ic19iYXRjaCkpICU+JQogICAgICBncm91cF9ieShyYXRpbywgcm9pLCByZl9pbmNyZWFzZSkgJT4lCiAgICAgIHVubmVzdCgpICU+JQogICAgICBncm91cF9ieShyYXRpbywgcm9pLCByZl9pbmNyZWFzZSwgLmlkKSAlPiUKICAgICAgZG8oYXMuZGF0YS5mcmFtZSguJHN0cmFwKSkgJT4lCiAgICAgIHN1bW1hcmlzZSh2YWx1ZT1tZWRpYW4odmFsdWUpKQogICAgZGZfYmF0Y2gkLmlkIDwtIGFzLm51bWVyaWMoZGZfYmF0Y2gkLmlkKSArIG5ic19iYXRjaCAqIChiYXRjaCAtIDEpCiAgICBkZl9icyA8LSByYmluZChkZl9icywgZGZfYmF0Y2gpCiAgfQogIAogICMgY29tcHV0ZSBjaXMKICBjaSA8LSA5NQogIGxfY2kgPC0gKDEwMCAtIGNpKS8yMDAKICBoX2NpIDwtIDEgLSBsX2NpCiAgY2lzIDwtCiAgZGZfYnMgJT4lCiAgICB1bmdyb3VwKCkgJT4lCiAgICBncm91cF9ieShyYXRpbywgcm9pLCByZl9pbmNyZWFzZSkgJT4lCiAgICBzdW1tYXJpc2UobG89cXVhbnRpbGUodmFsdWUsIGxfY2kpLAogICAgICAgICAgICAgIGhpPXF1YW50aWxlKHZhbHVlLCBoX2NpKSkKICAKICBkZl90MCA8LQogICAgZGYgJT4lCiAgICBncm91cF9ieShyYXRpbywgcm9pLCByZl9pbmNyZWFzZSkgJT4lCiAgICBzdW1tYXJpc2UodmFsdWU9bWVkaWFuKHZhbHVlKSkKICAKICBkZl90MCA8LQogIGRmX3QwICU+JQogICAgbWVyZ2UoY2lzKQogIAogIGRmX3QwJHJvaSA8LSBmYWN0b3IoZGZfdDAkcm9pLCBsZXZlbHM9YygnSU9HJywgJ3BGdXMnLCAnbUZ1cycpKQogIHdyaXRlX2NzdihkZl90MCwgb3V0cHV0X2ZuKQp9IGVsc2UgewogIGRmX3QwIDwtIHJlYWRfY3N2KG91dHB1dF9mbikgIAp9CmBgYAoKYGBge3J9CnRoZW1lX1B1YmxpY2F0aW9uIDwtIGZ1bmN0aW9uKGJhc2Vfc2l6ZT0xMikgewogICAgICBsaWJyYXJ5KGdndGhlbWVzKQogICAgICAodGhlbWVfZm91bmRhdGlvbihiYXNlX3NpemU9YmFzZV9zaXplKQogICAgICAgKyB0aGVtZShwbG90LnRpdGxlID0gZWxlbWVudF90ZXh0KGZhY2UgPSAiYm9sZCIsCiAgICAgICAgICAgICAgICAgICAgICAgICAgICAgICAgICAgICAgICAgc2l6ZSA9IHJlbCgxLjIpLCBoanVzdCA9IDAuNSksCiAgICAgICAgICAgICAgIHRleHQgPSBlbGVtZW50X3RleHQoKSwKICAgICAgICAgICAgICAgcGFuZWwuYmFja2dyb3VuZCA9IGVsZW1lbnRfcmVjdChjb2xvdXIgPSBOQSksCiAgICAgICAgICAgICAgIHBsb3QuYmFja2dyb3VuZCA9IGVsZW1lbnRfcmVjdChjb2xvdXIgPSBOQSksCiAgICAgICAgICAgICAgIHBhbmVsLmJvcmRlciA9IGVsZW1lbnRfcmVjdChjb2xvdXIgPSBOQSksCiAgICAgICAgICAgICAgIGF4aXMudGl0bGUgPSBlbGVtZW50X3RleHQoc2l6ZSA9IHJlbCgxKSksCiAgICAgICAgICAgICAgIGF4aXMudGl0bGUueSA9IGVsZW1lbnRfdGV4dChhbmdsZT05MCx2anVzdCA9MiksCiAgICAgICAgICAgICAgIGF4aXMudGl0bGUueCA9IGVsZW1lbnRfdGV4dCh2anVzdCA9IC0wLjIpLAogICAgICAgICAgICAgICBheGlzLnRleHQgPSBlbGVtZW50X3RleHQoKSwgCiAgICAgICAgICAgICAgIGF4aXMubGluZSA9IGVsZW1lbnRfbGluZShjb2xvdXI9ImJsYWNrIiksCiAgICAgICAgICAgICAgIGF4aXMudGlja3MgPSBlbGVtZW50X2xpbmUoKSwKICAgICAgICAgICAgICAgcGFuZWwuZ3JpZC5tYWpvciA9IGVsZW1lbnRfYmxhbmsoKSwgI2VsZW1lbnRfbGluZShjb2xvdXI9IiNmMGYwZjAiKSwKICAgICAgICAgICAgICAgcGFuZWwuZ3JpZC5taW5vciA9IGVsZW1lbnRfYmxhbmsoKSwKICAgICAgICAgICAgICAgbGVnZW5kLmtleSA9IGVsZW1lbnRfcmVjdChjb2xvdXIgPSBOQSksCiAgICAgICAgICAgICAgIGxlZ2VuZC5wb3NpdGlvbiA9ICJib3R0b20iLAogICAgICAgICAgICAgICBsZWdlbmQuZGlyZWN0aW9uID0gImhvcml6b250YWwiLAogICAgICAgICAgICAgICAjbGVnZW5kLmtleS5zaXplPSB1bml0KDAuMiwgImNtIiksCiAgICAgICAgICAgICAgIGxlZ2VuZC5zcGFjaW5nID0gdW5pdCgwLCAiY20iKSwKICAgICAgICAgICAgICAgbGVnZW5kLnRpdGxlID0gZWxlbWVudF90ZXh0KCksCiAgICAgICAgICAgICAgIHBsb3QubWFyZ2luID0gdW5pdChjKDEwLDUsNSw1KSwibW0iKSwKICAgICAgICAgICAgICAgc3RyaXAuYmFja2dyb3VuZCA9IGVsZW1lbnRfcmVjdChjb2xvdXI9IiNmMGYwZjAiLGZpbGw9IiNmMGYwZjAiKSwKICAgICAgICAgICAgICAgc3RyaXAudGV4dCA9IGVsZW1lbnRfdGV4dChmYWNlPSJib2xkIiksCiAgICAgICAgICAgICAgIHN0cmlwLnRleHQueSA9IGVsZW1lbnRfdGV4dChhbmdsZSA9IDApCiAgICAgICAgICApKQogICAgICAKfQoKYGBgCgpgYGB7cn0KZGZfdDBfcmZpMCA8LSBkZl90MCAlPiUKICBmaWx0ZXIocmZfaW5jcmVhc2UgPT0gMCkKCmlmICh0YXNrID09ICdlc3RpbWF0aW9uJykgewogIHlfbGltcyA8LSBjKDAsIDEuMjUpCn0gZWxzZSB7CiAgeV9saW1zIDwtIGMoMCwgMC44KQp9CmBgYApgYGB7ciBmaWcuaGVpZ2h0PTMsIGZpZy53aWR0aD00fQpkZl90MF9yZmkwICU+JQogIGdncGxvdChhZXMocmF0aW8sIHZhbHVlLCB5bWluPWxvLCB5bWF4PWhpLCBjb2xvcj1yb2ksIGdyb3VwPXJvaSkpICsKICBnZW9tX2xpbmVyYW5nZShzaG93LmxlZ2VuZD1GKSArCiAgZ2VvbV9saW5lKHNob3cubGVnZW5kPUYpICsKICBnZW9tX3BvaW50KHNpemU9MS41KSArCiAgbGFicyh4PSJSYXRpbyBvZiB1bml0cyBzZWxlY3RpdmUgdG8gaWRlbnRpdHkgYSIsCiAgICAgICB5PSJTaW11bGF0ZWQgUFNFIHZhcmlhbmNlIiwKICAgICAgIGNvbG9yPSIiKSArCiAgdGhlbWVfUHVibGljYXRpb24oKSArCiAgdGhlbWUobGVnZW5kLmRpcmVjdGlvbj0ndmVydGljYWwnLAogICAgICAgIGxlZ2VuZC5wb3NpdGlvbj1jKDAuOCwgMC44KSkgKwogIHNjYWxlX2NvbG9yX2JyZXdlcihwYWxldHRlPSdTZXQxJykgKwogIGNvb3JkX2NhcnRlc2lhbih5bGltPXlfbGltcykKCiNpbWcgPC0gcGFzdGUoaW1nZm4sICdfZGF0YS5wbmcnLCBzZXA9JycpCiNnZ3NhdmUoaW1nLCBkcGk9MzAwLCBoZWlnaHQ9Mywgd2lkdGg9NCkKYGBgCmBgYHtyfQojIG1ha2UgbXkgb3duIHBhbGV0dGVzIHRvIGF2b2lkIHRvbyBsaWdodCBjb2xvcnMKbGlicmFyeShSQ29sb3JCcmV3ZXIpCgpyZWQgPC0gYnJld2VyLnBhbChuPTksICJSZWRzIikKcmVkX3BhbGV0dGUgPC0gY29sb3JSYW1wUGFsZXR0ZShjKHJlZFszXSwgcmVkWzZdLCByZWRbOV0pLCBzcGFjZSA9ICJMYWIiKQpteV9yZWQgPC0gcmVkX3BhbGV0dGUoNykKYmx1ZSA8LSBicmV3ZXIucGFsKG49OSwgIkJsdWVzIikKYmx1ZV9wYWxldHRlIDwtIGNvbG9yUmFtcFBhbGV0dGUoYyhibHVlWzNdLCBibHVlWzZdLCBibHVlWzldKSwgc3BhY2UgPSAiTGFiIikKbXlfYmx1ZSA8LSBibHVlX3BhbGV0dGUoNykKZ3JlZW4gPC0gYnJld2VyLnBhbChuPTksICJHcmVlbnMiKQpncmVlbl9wYWxldHRlIDwtIGNvbG9yUmFtcFBhbGV0dGUoYyhncmVlblszXSwgZ3JlZW5bNl0sIGdyZWVuWzldKSwgc3BhY2UgPSAiTGFiIikKbXlfZ3JlZW4gPC0gZ3JlZW5fcGFsZXR0ZSg3KQpgYGAKYGBge3IgZmlnLmhlaWdodD0yLjI1LCBmaWcud2lkdGg9M30KZGZfdDBfaW9nIDwtIGZpbHRlcihkZl90MCwgcm9pID09ICdJT0cnKQpkZl90MF9pb2ckcmZfaW5jcmVhc2UgPC0gYXMubnVtZXJpYyhhcy5jaGFyYWN0ZXIoZGZfdDBfaW9nJHJmX2luY3JlYXNlKSkgKiAxMDAKZGZfdDBfaW9nJHJmX2luY3JlYXNlIDwtIGFzLmZhY3RvcihkZl90MF9pb2ckcmZfaW5jcmVhc2UpCgpkZl90MF9pb2cgJT4lCiAgZ2dwbG90KGFlcyhyYXRpbywgdmFsdWUsIGNvbG9yPXJmX2luY3JlYXNlLCBncm91cD1yZl9pbmNyZWFzZSkpICsKICBnZW9tX2xpbmUoc2hvdy5sZWdlbmQ9RikgKwogIGdlb21fcG9pbnQoc2l6ZT0xLjUpICsKICBsYWJzKHg9IlJhdGlvIG9mIHVuaXRzIHNlbGVjdGl2ZSB0byBpZGVudGl0eSBhIiwKICAgICAgIHk9IlNpbXVsYXRlZCBQU0UgdmFyaWFuY2UiLAogICAgICAgY29sb3I9IlJGIGluY3JlYXNlIFslXSIpICsKICB0aGVtZV9QdWJsaWNhdGlvbihiYXNlX3NpemU9MTApICsKICB0aGVtZShsZWdlbmQuZGlyZWN0aW9uPSd2ZXJ0aWNhbCcsCiAgICAgICAgbGVnZW5kLnBvc2l0aW9uPWMoMC44LCAwLjgpLAogICAgICAgIGxlZ2VuZC50aXRsZT1lbGVtZW50X3RleHQoc2l6ZT0xMCksCiAgICAgICAgbGVnZW5kLmtleS5zaXplPXVuaXQoMTAsICdwdCcpLAogICAgICAgIGxlZ2VuZC50ZXh0PWVsZW1lbnRfdGV4dChzaXplPTgpKSArIAogIHNjYWxlX2NvbG9yX21hbnVhbCh2YWx1ZXM9bXlfcmVkKSArCiAgY29vcmRfY2FydGVzaWFuKHlsaW09eV9saW1zKQoKaW1nIDwtIHBhc3RlKGltZ2ZuLCAnX2luY3JlYXNlLXJmc2l6ZV9pb2cucG5nJywgc2VwPScnKQpnZ3NhdmUoaW1nLCBkcGk9MzAwLCBoZWlnaHQ9Mi4yNSwgd2lkdGg9My4pCmBgYApgYGB7ciBmaWcuaGVpZ2h0PTIuMjUsIGZpZy53aWR0aD0zfQpkZl90MF9wZnVzIDwtIGZpbHRlcihkZl90MCwgcm9pID09ICdwRnVzJykKZGZfdDBfcGZ1cyRyZl9pbmNyZWFzZSA8LSBhcy5udW1lcmljKGFzLmNoYXJhY3RlcihkZl90MF9wZnVzJHJmX2luY3JlYXNlKSkgKiAxMDAKZGZfdDBfcGZ1cyRyZl9pbmNyZWFzZSA8LSBhcy5mYWN0b3IoZGZfdDBfcGZ1cyRyZl9pbmNyZWFzZSkKCmRmX3QwX3BmdXMgJT4lCiAgZ2dwbG90KGFlcyhyYXRpbywgdmFsdWUsIGNvbG9yPXJmX2luY3JlYXNlLCBncm91cD1yZl9pbmNyZWFzZSkpICsKICBnZW9tX2xpbmUoc2hvdy5sZWdlbmQ9RikgKwogIGdlb21fcG9pbnQoc2l6ZT0xLjUpICsKICBsYWJzKHg9IlJhdGlvIG9mIHVuaXRzIHNlbGVjdGl2ZSB0byBpZGVudGl0eSBhIiwKICAgICAgIHk9IlNpbXVsYXRlZCBQU0UgdmFyaWFuY2UiLAogICAgICAgY29sb3I9IlJGIGluY3JlYXNlIFslXSIpICsKICB0aGVtZV9QdWJsaWNhdGlvbihiYXNlX3NpemU9MTApICsKICB0aGVtZShsZWdlbmQuZGlyZWN0aW9uPSd2ZXJ0aWNhbCcsCiAgICAgICAgbGVnZW5kLnBvc2l0aW9uPWMoMC44LCAwLjgpLAogICAgICAgIGxlZ2VuZC50aXRsZT1lbGVtZW50X3RleHQoc2l6ZT0xMCksCiAgICAgICAgbGVnZW5kLmtleS5zaXplPXVuaXQoMTAsICdwdCcpLAogICAgICAgIGxlZ2VuZC50ZXh0PWVsZW1lbnRfdGV4dChzaXplPTgpKSArIAogIHNjYWxlX2NvbG9yX21hbnVhbCh2YWx1ZXM9bXlfYmx1ZSkgKwogIGNvb3JkX2NhcnRlc2lhbih5bGltPXlfbGltcykKCmltZyA8LSBwYXN0ZShpbWdmbiwgJ19pbmNyZWFzZS1yZnNpemVfcGZ1cy5wbmcnLCBzZXA9JycpCmdnc2F2ZShpbWcsIGRwaT0zMDAsIGhlaWdodD0yLjI1LCB3aWR0aD0zLikKYGBgCgpgYGB7ciBmaWcuaGVpZ2h0PTIuMjUsIGZpZy53aWR0aD0zfQpkZl90MF9tZnVzIDwtIGZpbHRlcihkZl90MCwgcm9pID09ICdtRnVzJykKZGZfdDBfbWZ1cyRyZl9pbmNyZWFzZSA8LSBhcy5udW1lcmljKGFzLmNoYXJhY3RlcihkZl90MF9tZnVzJHJmX2luY3JlYXNlKSkgKiAxMDAKZGZfdDBfbWZ1cyRyZl9pbmNyZWFzZSA8LSBhcy5mYWN0b3IoZGZfdDBfbWZ1cyRyZl9pbmNyZWFzZSkKCmRmX3QwX21mdXMgJT4lCiAgZ2dwbG90KGFlcyhyYXRpbywgdmFsdWUsIGNvbG9yPXJmX2luY3JlYXNlLCBncm91cD1yZl9pbmNyZWFzZSkpICsKICBnZW9tX2xpbmUoc2hvdy5sZWdlbmQ9RikgKwogIGdlb21fcG9pbnQoc2l6ZT0xLjUpICsKICBsYWJzKHg9IlJhdGlvIG9mIHVuaXRzIHNlbGVjdGl2ZSB0byBpZGVudGl0eSBhIiwKICAgICAgIHk9IlNpbXVsYXRlZCBQU0UgdmFyaWFuY2UiLAogICAgICAgY29sb3I9IlJGIGluY3JlYXNlIFslXSIpICsKICB0aGVtZV9QdWJsaWNhdGlvbihiYXNlX3NpemU9MTApICsKICB0aGVtZShsZWdlbmQuZGlyZWN0aW9uPSd2ZXJ0aWNhbCcsCiAgICAgICAgbGVnZW5kLnBvc2l0aW9uPWMoMC44LCAwLjgpLAogICAgICAgIGxlZ2VuZC50aXRsZT1lbGVtZW50X3RleHQoc2l6ZT0xMCksCiAgICAgICAgbGVnZW5kLmtleS5zaXplPXVuaXQoMTAsICdwdCcpLAogICAgICAgIGxlZ2VuZC50ZXh0PWVsZW1lbnRfdGV4dChzaXplPTgpKSArIAogIHNjYWxlX2NvbG9yX21hbnVhbCh2YWx1ZXM9bXlfZ3JlZW4pICsKICBjb29yZF9jYXJ0ZXNpYW4oeWxpbT15X2xpbXMpCgppbWcgPC0gcGFzdGUoaW1nZm4sICdfaW5jcmVhc2UtcmZzaXplX21mdXMucG5nJywgc2VwPScnKQpnZ3NhdmUoaW1nLCBkcGk9MzAwLCBoZWlnaHQ9Mi4yNSwgd2lkdGg9My4pCmBgYAo=
